# Meal-induced changes in cardiometabolic peptides in the context of acute cardiovascular diseases and glucose metabolism

**DOI:** 10.1186/s12933-026-03424-5

**Published:** 2026-10-26

**Authors:** G. Lishkova, O. Schweizerhof, J. Großmann, K. von Rohr, U. Landmesser, F. Edelmann, K. U. Eckardt, M. Endres, J. E. Weber, U. Grittner, T. Pischon, J. Schulz-Menger, D. N. Müller, J. Spranger, L. Li, K. Mai

**Affiliations:** 1 Department of Endocrinology and Metabolism, Charité-Universitätsmedizin Berlin, Corporate Member of the Freie Universität Berlin and Humboldt-Universität Berlin, ENDO-ERN, Berlin, Germany; 2 German Centre for Cardiovascular Research (DZHK), Partner Site Berlin, Berlin, Germany; 3 German Center for Diabetes Research (DZD), München-Neuherberg, Germany; 4 Berlin Institute of Health at Charité - Universitätsmedizin Berlin, Berlin, Germany; 5 Institute of Biometry and Clinical Epidemiology, Charité-Universitätsmedizin Berlin, Corporate Member of the Freie Universität Berlin and Humboldt-Universität Berlin, Berlin, Germany; 6 Deutsches Herzzentrum der Charité (DHZC), Department of Cardiology, Angiology and Intensive Care Medicine, Campus Virchow Klinikum, Berlin, Germany; 7 Charité-Universitätsmedizin Berlin, Corporate Member of Freie Universität Berlin and Humboldt-Universität Berlin, Berlin, Germany; 8 Department of Nephrology and Medical Intensive Care, Charité-Universitätsmedizin Berlin, Corporate Member of the Freie Universität Berlin and Humboldt-Universität Berlin, Berlin, Germany; 9 Department of Neurology with Experimental Neurology, Charité-Universitätsmedizin Berlin, Corporate Member of the Freie Universität Berlin and Humboldt-Universität Berlin, Berlin, Germany; 10 Center for Stroke Research (CSB), Charité-Universitätsmedizin Berlin, Corporate Member of the Freie Universität Berlin and Humboldt-Universität Berlin, Berlin, Germany; 11 German Center for Neurodegenerative Diseases (DZNE), Partner Site Berlin, Berlin, Germany; 12 German Center for Mental Health (DZPG), Partner Site Berlin, Berlin, Germany; 13 Max-Delbrück-Center for Molecular Medicine in the Helmholtz Association (MDC), Berlin, Germany; 14 Biobank Technology Platform, Max Delbrück Center for Molecular Medicine in the Helmholtz Association (MDC), Berlin, Germany; 15 Experimental and Clinical Research Center, A Cooperation of Charité- Universitätsmedizin Berlin and Max Delbrück Center for Molecular Medicine, 13125 Berlin, Germany; 16 Department of Human Nutrition, German Institute of Human Nutrition Potsdam-Rehbrücke, Nuthetal, Germany; 17 Max Rubner Center for Cardiovascular Metabolic Renal Research, Charité- Universitätsmedizin Berlin, Corporate Member of the Freie Universität Berlin and Humboldt-Universität Berlin, Berlin, Germany

**Keywords:** Natriuretic peptides, MR-proANP, NT-proBNP, Copeptin, Mixed meal test, Insulin resistance, Type 2 diabetes, Cardiovascular disease, Postprandial metabolism

## Abstract

**Background:**

Natriuretic peptides (NPs) and copeptin are established cardiovascular biomarkers which can partly affect human metabolism. However, impact of human metabolism on their postprandial regulation in high-risk patients remains insufficiently understood. We aimed to characterize meal-induced changes in MR-proANP, NT-proBNP, and copeptin and to assess how these dynamics relate to acute cardiovascular (CV) events, type 2 diabetes (T2D) and insulin resistance (IR).

**Methods:**

Within the BeLOVE cohort, a subset of 373 participants with acute coronary syndrome, stroke or very high chronic CV risk underwent standardized study-specific mixed-meal testing approximately 3 months after the acute event or 1 month after enrollment for the chronic high-risk group. Fasting and 120-min postprandial concentrations of MR-proANP, NT-proBNP, copeptin, insulin and glucose were assessed. Structural equation models were used to evaluate direct and indirect associations of acute CV events, T2D, and IR (HOMA-IR) with fasting levels and postprandial changes, adjusted for age, sex and BMI.

**Results:**

MR-proANP showed the most pronounced meal-induced suppression. NT-proBNP demonstrated only minor and inconsistent postprandial changes. For all peptides, fasting concentrations were the main determinant of their postprandial changes. A recent acute CV event was associated with higher fasting NP levels (MR-proANP: standardized β = 0.22 [0.09; 0.34]; NT-proBNP: β = 0.28 [0.15; 0.41]), but not substantially with their postprandial changes after accounting for their baseline levels. T2D showed no consistent association with fasting or postprandial NP responses. In contrast, higher HOMA-IR was associated with lower fasting MR-proANP (β=−0.19 [−0.30; −0.07]) and a stronger postprandial decline (β=−0.18 [−0.33; −0.04]). Similarly, higher HOMA-IR was linked to lower fasting NT-proBNP (β=−0.15 [−0.28; −0.03]) but showed no clear association to its postprandial change. Copeptin dynamics were largely independent of acute CV disease and metabolic status.

**Conclusions:**

In patients after a recent ACS or stroke, postprandial MR-proANP dynamics are linked to insulin resistance, rather than diabetes status. In contrast, the limited postprandial variability of NT-proBNP and the largely fasting-related changes of copeptin make these markers less suited for capturing short-term metabolic responses. Together, these findings highlight the value of postprandial phenotyping for understanding cardiometabolic regulation and support further evaluation of MR-proANP as a potential biomarker in future prospective studies with cardiovascular outcomes across subjects with and without acute CV event.

**Trial registration:**

The study protocol was approved by the institutional ethics committee (EA1/066/17) and registered in the German Clinical Trials Register (DRKS00016852).

**Supplementary Information:**

The online version contains supplementary material available at https://doi.org/10.1186/s12933-026-03424-5.

## Research insights

**What is currently known about this topic?:**

Natriuretic peptides are well-established cardiometabolic hormones that both influence and are regulated by human metabolism. However, postprandial NP dynamics and the impact of metabolism in high-risk patients are poorly characterized.

**What is the key research question?:**

How do acute cardiovascular events, type 2 diabetes and insulin resistance shape postprandial MR-proANP, NT-proBNP and copeptin dynamics?

**What is new?:**

Insulin resistance, rather than diabetes status, is associated with postprandial MR-proANP suppression in a high-risk population. Acute CV events elevate fasting NP levels but are not independently linked to postprandial responses. Copeptin dynamics appear independent of metabolic status.

**How might this study influence clinical practice?:**

Postprandial MR-proANP phenotyping may improve cardiometabolic risk stratification in high-risk populations.

## Graphical Abstract

**Figure:**
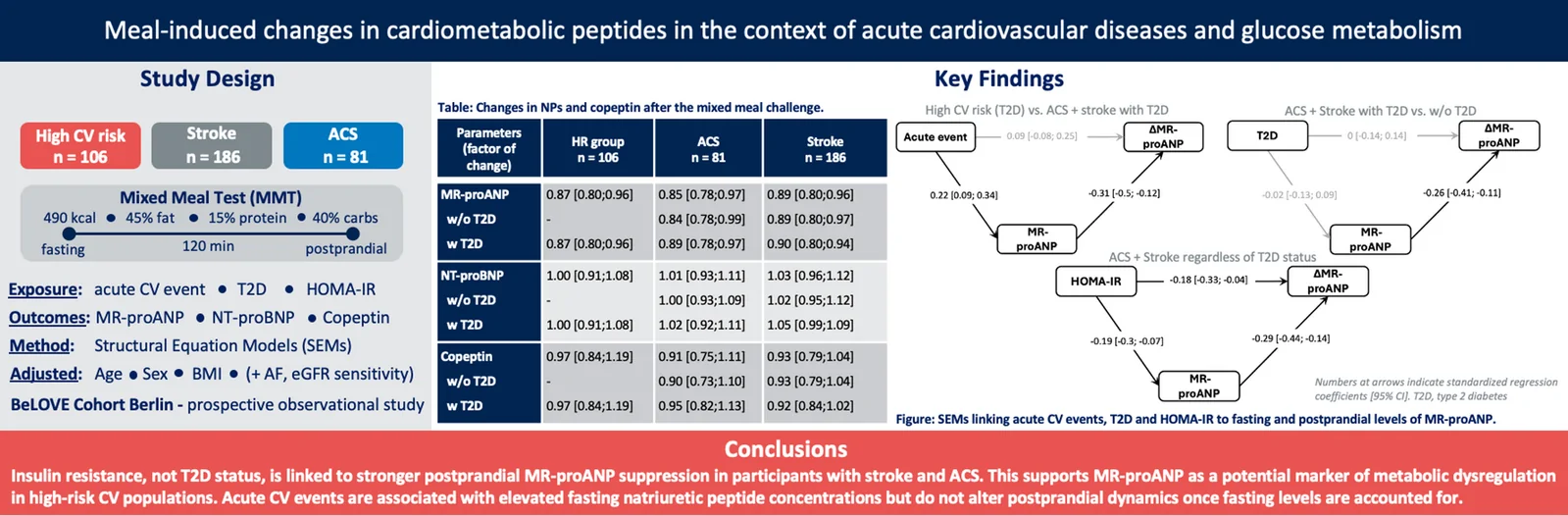

## Introduction

Natriuretic peptides (NPs) and copeptin are well-established biomarkers in cardiovascular disease (CVD). Atrial natriuretic peptide (ANP) and B-type natriuretic peptide (BNP), released by cardiomyocytes in response to volume and pressure load, provide robust diagnostic and prognostic information in heart failure and other cardiovascular conditions [1]. Elevated copeptin, a stable surrogate of arginine vasopressin secretion, has also been consistently associated with adverse outcomes and higher mortality risk in various cardiovascular conditions [2]. In patients with heart failure after acute myocardial infarction, copeptin was a strong predictor of mortality and recurrent events, even outperforming BNP/NT-proBNP in the OPTIMAAL study [2], underscoring its value as a marker of CV risk.

Beyond hemodynamic stress, metabolic factors have also been shown to modulate NP physiology [3]. In fact, dysregulation of NPs has been associated with obesity, insulin resistance (IR) and related conditions like hypertension and impaired glucose tolerance. Large cohort studies indicate that IR, more than obesity per se, is associated with 10–30% lower NT-proBNP levels [4–6]. Mechanistically, an upregulation of natriuretic clearance receptor (NPR-C) by hyperinsulinemia was demonstrated in human subcutaneous adipose tissue [7]. In obesity, this receptor is often overexpressed and the ratio between its active form (NPR-A) and clearance form (NPR-C) is shifted, leading to weaker natriuretic peptide signaling, a phenomenon referred to as the “natriuretic handicap” of obesity [8–10]. In parallel, copeptin is likewise associated with adverse metabolic profiles, showing links with IR, predicting incident diabetes and associating independently of insulin with metabolic syndrome components such as central obesity, hypertension and dysglycemia [11, 12]. Notably, visceral adiposity and obesity are linked to higher copeptin, underscoring its role as a marker of adverse cardiometabolic risk.

The dynamic regulation of NPs and copeptin in response to nutrient intake is an emerging, but still poorly characterized dimension of this system. This might be particularly relevant, as the postprandial state of other cardiometabolic markers such as glucose or triacylglycerol levels, is more predictive for future cardiovascular events than the respective fasting levels [13, 14]. Prior research indicates that glucose intake suppresses MR-proANP secretion, an effect observed in both lean and obese individuals [10, 15], suggesting a functional link between nutrient metabolism and cardiac endocrine function. Postprandial BNP responses are less reproducible and show rather mixed patterns following nutrient intake [16–19]. In the CoMEAL study, copeptin concentrations showed a modest postprandial decrease in healthy volunteers after both glucose and mixed meals, without differences between nutrient types [20]. This supports the view that higher copeptin levels may reflect chronic metabolic stress rather than an acute meal-induced response. Nonetheless, elevated copeptin is also linked to insulin resistance and abdominal obesity [21], making its regulation in cardiometabolic diseases interesting.

While the interplay between metabolism and cardiac peptides is increasingly recognized, key clinical modifiers of these dynamics remain unclear. Most prior studies of postprandial NPs or copeptin changes were done in healthy or obese individuals without acute CVD [20, 22–24]. It is unknown to what extent diabetes status or the degree of insulin resistance might alter meal-induced NP/copeptin responses in such high-risk populations - gaps that limit translation of peptide physiology into actionable cardiometabolic phenotyping.

The Berlin Long-term Observation of Vascular Events (BeLOVE) study offers a unique opportunity to address these knowledge gaps in a high-risk population. By analyzing changes in MR-proANP, NT-proBNP, and copeptin after a standardized mixed meal test, we provide, to our knowledge, the first characterization of postprandial cardiometabolic peptide changes in an acute, high-cardiovascular-risk population. By modeling their associations with acute cardiovascular event history, type 2 diabetes and insulin resistance we aim to provide novel insights into how metabolic state is linked to cardiometabolic peptide changes and whether the continuous degree of insulin resistance relates to peptide regulation differently from the categorical diagnosis of diabetes. Establishing these links could refine biomarker interpretation in CVD and motivate the use of postprandial peptide responses as candidate tools for cardiometabolic risk stratification.

## Methods

### Study design and population

BeLOVE (Berlin Long-term Observation of Vascular Events) is a prospective observational cohort study of adult participants with high-cardiovascular-risk conducted at the Charité-Universitätsmedizin Berlin, the Max-Delbrück Center for Molecular Medicine and the Berlin Institute of Health. Participants are recruited at the clinical campuses of Charité-Universitätsmedizin Berlin and include both participants with acute CV events and participants without recent events but categorized as very high cardiovascular risk [25]. The study protocol was approved by the institutional ethics committee (EA1/066/17) and registered in the German Clinical Trials Register (DRKS00016852).

#### Inclusion criteria

were age ≥ 18 years, ability to consent and willingness to participate and the presence of one of 5 conditions indicating high CV risk: acute coronary syndrome (ACS), acute cerebrovascular event, acute heart failure, acute kidney injury (AKI) or very-high-risk chronic CV condition (HR) without event in the preceding 12 months. For the acute ACS and stroke cohorts, participants were enrolled within 0–7 days after the index event. ACS was defined by typical chest pain, ECG changes consistent with myocardial infarction, and/or dynamic cardiac troponin. Stroke and TIA required an acute-onset neurological deficit with corresponding imaging findings or symptom duration for > 24 h or, for TIA, full recovery within 24 h. The very-high-risk chronic group included participants without a recent event but with established CV disease or equivalent risk. For the sample used in this study, these were patients with diabetes mellitus type 2 with or without additional risk factors (arterial hypertension, hypercholesterolemia) or target organ damage (see Weber et al. for more details) [25].

#### Exclusion criteria

included inability to give informed consent, pregnancy or breastfeeding, lack of health insurance, life expectancy < 6 months due to a non-cardiovascular condition, active malignancy or a history of solid organ transplantation.

Participants meeting all inclusion criteria and no exclusion criteria were eligible for enrollment. All participants provided written informed consent before the first study-related action was performed and could withdraw from study participation at any given time without the need for justification.

The present analysis represents a predefined sub-analysis of the BeLOVE cohort and was restricted to participants with ACS, stroke or very high chronic CV risk. Participants were excluded from the analysis if no meal test was performed at the deep phenotyping visit, if the meal test was not fully completed or if the predefined timing between meal ingestion and postprandial blood sampling was not met. In addition, participants with diabetes types other than type 2 diabetes (e.g. type 1 diabetes, MODY) were excluded.

### Recruitment

Participants of the acute cardiovascular event groups were enrolled during the acute hospitalization at the Department of Neurology or Cardiology of the Charité-Universitätsmedizin Berlin, respectively (within 0–7 days after the event). Participants of the HR group were primarily recruited through the outpatient cardiology and endocrinology care units at Charité.

### Phenotyping

#### Initial phenotyping (acute phase)

All participants underwent an initial assessment soon after enrollment. For acute event participants, this was performed within 7 days of the index event, for the chronic high-risk participants within 14 days of study inclusion. This initial phenotyping included key measurements and biosamples (fasting blood and spot urine) collection. Demographic data, medical history, weight, height and acute clinical course were recorded, including comorbidities and treatments.

#### Deep phenotyping

Participants were reinvited for an extensive in-person evaluation at one of the dedicated BeLOVE clinical research units. This visit was scheduled around 3 months after the acute index event for the ACS and stroke cohorts, and approximately 1 month after study inclusion for the HR cohort. The deep phenotyping protocol included data on socio-demographics and lifestyle, medical history, physical examination and vital signs, cardiovascular, neurological, and metabolic assessments as previously reported [25]. Participants were assigned to either a nutritional challenge with metabolic phenotyping or a physical challenge (ergometry). Randomization was performed when no contraindications for either of the two challenges were present. The present analysis focuses exclusively on participants who underwent the nutritional challenge, which included fasting blood samples as well as a mixed meal test.

### Mixed Meal Test

A study-specific standardized Mixed Meal Test (MMT) was conducted as part of the metabolic phenotyping during the deep phenotyping visit to assess postprandial metabolic responses. Participants arrived at the Clinical Research Unit after an overnight fast of at least 8 h, refraining from any caloric intake, caffeine, nicotine and alcohol. After collection of fasting blood samples for baseline glucose, insulin, NT-proBNP, MR-proANP, copeptin, lipids and other markers, participants consumed a liquid test meal specifically designed for the BeLOVE study.

**Fig. 1:**
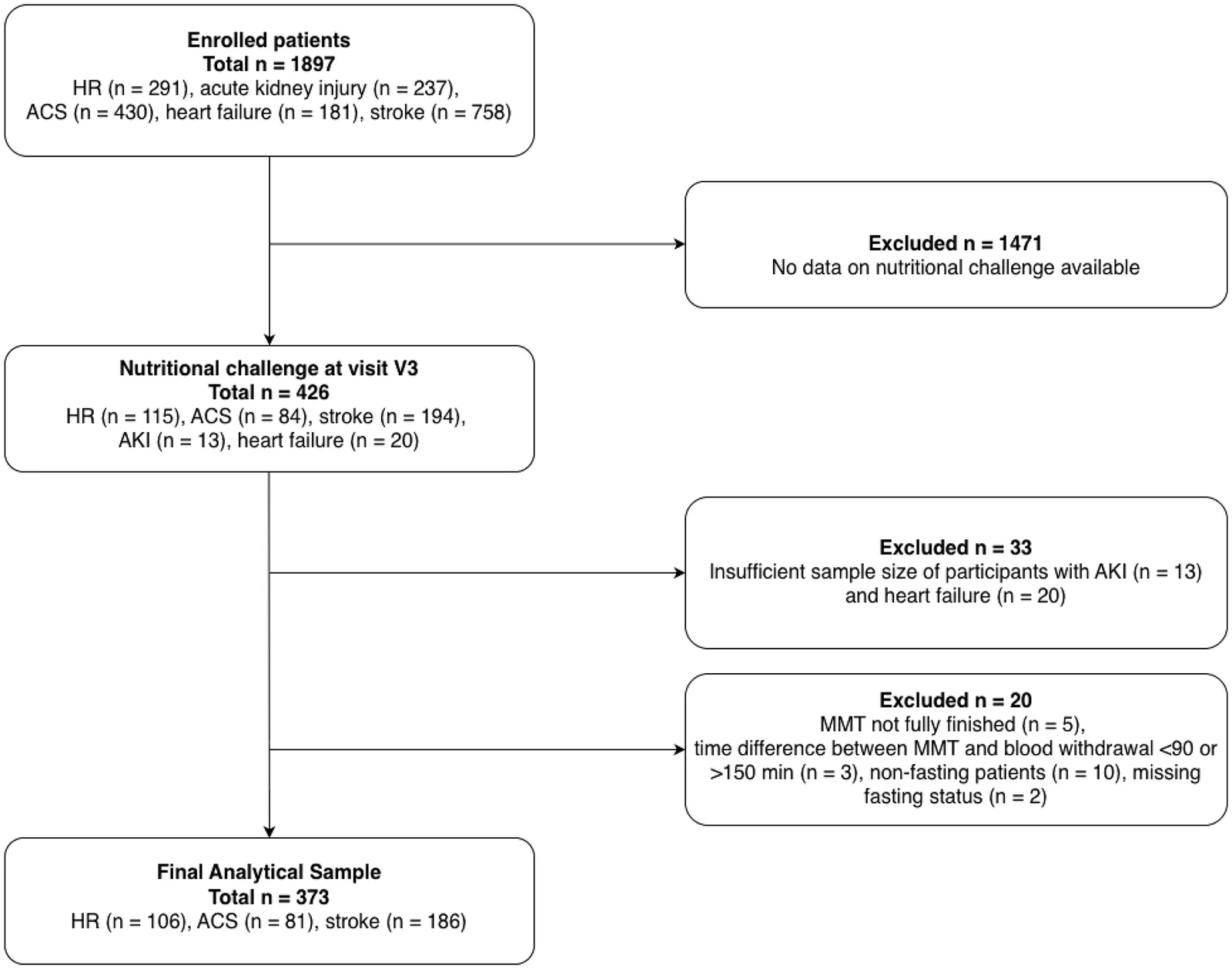
STROBE flow chart of the study. V3, deep phenotyping visit

In contrast to conventional mixed meal tests, which are typically carbohydrate-rich and lower in fat and protein [26–28], the BeLOVE meal challenge was deliberately designed as a fat-enriched liquid meal to better reflect the increasing dietary fat intake observed in the German population in recent decades [29, 30]. The test provided approximately 500 kcal of energy, with a macronutrient composition of 15% protein, 45% fat, and 40% carbohydrates (Table 1). Participants were instructed to consume the meal within 5 min under supervision and timing was started once the drink had been finished. A second blood sample was collected 120 min after meal completion. Sampling adhered closely to this schedule (median 120 min, IQR 118–120). A predefined acceptance window of 90 to 150 min was applied and participants with an interval outside this window were excluded (Fig. 1). This time point falls within the conventional 2-hour postprandial assessment window used in standardized glucose and mixed-meal tolerance testing [14, 28] and represents an interval over which glucose-induced hyperinsulinemia has been shown to suppress MR-proANP in controlled studies [23]. Moreover, previous data on MR-proANP trajectories further indicate a continuous postprandial decline with an almost maximal response after 120 min [10]. For NT-proBNP, repeated sampling over 6 h after a carbohydrate load showed no relevant change at any time point [16]. For copeptin, the lowest postprandial concentrations after a standardized liquid mixed meal were observed at 120 min [20]. A single postprandial sample after 120 min was therefore chosen as a pragmatic approach suitable for a large high-risk cohort while minimizing participant burden. The test meal was prepared as a liquid drink containing 300 mL of still water (Table 1), so all participants received a defined fluid load as part of the challenge. During the 120-minute postprandial period, small sips of still water were permitted if urgently needed. Individual intake was not separately documented. No other caloric intake, smoking or strenuous physical activity was allowed for the duration of the test.

**Table 1:** Composition and nutritional content of the BeLOVE test meal

Ingredient	Amount	Protein (g)	Fat (g)	Carbohydrates (g)	Energy (kcal)
Optifast Vanilla Drink	52 g	18.9	5.8	18.0	204.0
Rapeseed oil	20 g	0.0	18.3	0.0	163.8
Maltodextrin	32 g	0.0	0.0	30.7	122.9
Water	300 mL	-	-	-	-
**Total**:	-	**18.9 g**	**24.1 g**	**48.7 g**	**490.7 kcal**
**Energy distribution**:	-	**15%**	**45%**	**40%**	-

### Laboratory analyses

All laboratory measurements were performed in a central certified laboratory in accordance with the manufacturers’ protocols. Samples obtained at the inclusion as well as day 90 visits were processed immediately and analyzed or stored at -80 °C for batch analysis of research biomarkers. Routine clinical chemistry (e.g. electrolytes, renal and liver function, high-sensitivity troponin, HbA1c) and hematology were measured on the day of collection using automated analyzers as per hospital standards. Laboratory personnel were blinded to clinical outcomes when performing batched analyses of study-specific biomarkers. Laboratory analyses are described in detail in the supplements.

### Data storage

All baseline and follow-up data were captured in a pseudonymized form using a secure electronic case report form system (REDCap [31, 32], hosted at Charité) in accordance with the study’s data protection concept [25].

### Sample size and comparative groups

For the present analysis, participants who underwent an MMT at the deep phenotyping visit were selected from the BeLOVE cohort. Participants from the AKI and heart failure cohorts were excluded, as only 13 and 20 participants, respectively, completed the nutritional challenge during the visit. These sample sizes would have been insufficient to support the multivariable SEM approach used in the analysis. Pooling these participants with the ACS and stroke groups was not considered appropriate, as AKI does not represent the type of acute CV event addressed in this analysis and natriuretic peptide concentrations differ fundamentally in heart failure. The remaining participants were categorized into three predefined groups: a high-risk reference group (HR), participants with acute coronary syndrome (ACS), and participants with acute cerebrovascular disorder (stroke).

### Statistical analysis

Descriptive statistics for continuous variables are given as median with limits of the interquartile range (IQR, [25th; 75th percentile]). Categorical variables were summarized as counts and percentages. For descriptive comparisons of different inclusion entities, demographic characteristics, fasting values of metabolic parameters and absolute fasting and postprandial concentrations of the cardiometabolic peptides were reported (Table 2). The postprandial response was descriptively presented as the ratio of postprandial to fasting concentrations (Table 3).

#### Structural Equation Modeling

We used Structural Equation Models (SEM) to explore whether specific cardiovascular (acute CV events) or metabolic conditions (T2D and IR), hereafter referred to as exposures, are associated with postprandial peptide change independently of the fasting concentration (direct path), through their association with fasting concentration (indirect path), or both (Supplementary Fig. 1). This approach was chosen over conventional regression analyses because postprandial changes are inherently dependent on fasting concentrations, while fasting concentrations may already be associated with exposures. In observational studies, simple baseline adjustment can lead to biased estimates when baseline variables lie on the pathway between the exposure and outcome [33]. Postprandial change was modeled as a function of both fasting concentration and exposure, while fasting concentration itself was modeled as a function of exposure. In the SEMs, the fasting peptide concentration is treated as an intermediate variable. It is a possible consequence of the exposure (acute CV event, T2D or insulin resistance) and at the same time the starting point of the postprandial response. The indirect path runs from the exposure via the fasting concentration to the postprandial change. The fasting concentration thus takes a mediator-type role on this path. The direct path is the association of the exposure with the postprandial change at a given fasting concentration. Both paths are estimated simultaneously, without assuming a priori that one predominates. This is a statistical decomposition of the association between exposure and postprandial change into a direct and an indirect path. Given the observational, cross-sectional design, the estimates are interpreted as associations and not as causal effects.

Associations of exposures (acute CV events, T2D, IR) with fasting peptide concentrations and postprandial changes were additionally adjusted for age, sex and BMI as potential confounders in all models.

Due to lognormal distributions, cardiometabolic peptides were transformed using the natural log before using SEMs. Postprandial changes were expressed as log-transformed ratios of postprandial to fasting concentrations. Insulin resistance was represented by the homeostatic model assessment of Insulin Resistance Index (HOMA-IR) calculated from fasting glucose and fasting insulin concentrations as described previously by Matthews et al. [34]. As these were obtained immediately before the meal, HOMA-IR characterizes the metabolic state in which the meal was administered. As no standardized oral glucose tolerance test was performed, indices based on postprandial glucose or insulin levels could not be analyzed. Similar to cardiometabolic peptides, HOMA-IR was log-transformed, as recommended for non-normally distributed HOMA estimates [35].

To investigate the association of a recent acute CV event (exposure i) with postprandial changes of cardiometabolic peptides, the analyses were restricted to the T2D participants from the acute CV event groups (ACS or stroke) and the HR group. Correspondingly, we explored the association of T2D presence (exposure ii) with postprandial peptide changes in participants only from the acute event groups, seeking to reduce confounding from acute events. To assess the link between insulin resistance (exposure iii) and cardiometabolic peptides, we analyzed all participants from the acute event groups, irrespective of diabetes status. The high-risk reference group was not included here as it consists entirely of participants with T2D and would confound the association of HOMA-IR with group membership. The same path structure was applied in all three models, with the exposure variable and analytic sample differing as described above. For the analysis of acute CV events, ACS and stroke were pooled into a single acute-event group. Both conditions share some common neurohormonal and metabolic stress responses but differ in their pathophysiology and in the course of the acute response. Our exploratory event-type stratified analyses showed no major differences in associations with postprandial peptide changes between ACS and stroke. Given this consistency and as stratification substantially reduces the sample size within each subgroup, we pooled ACS and stroke for the main analysis to enable higher precision on estimates. To assess whether pooling these conditions may have obscured any clinically meaningful differences, we included fully stratified ACS- and stroke-specific models for each exposure and peptide in Supplementary Figs. 3–5 and Supplementary Tables 2–3, 5–6 and 8–9. To further quantify the contrast between the ACS and stroke groups for the acute CV event models, in which event type is the exposure itself, we estimated the contrast between ACS and stroke coefficients within the model. For the T2D and HOMA-IR models, which are restricted to the acute event groups, we added an exposure × event type interaction term. These contrast estimates are reported in Supplementary Table 13. Additionally, we performed sensitivity analyses including the presence of atrial fibrillation (AF) and renal function (eGFR) as further confounders, both recognized modulators of natriuretic peptides. As glucose-lowering medications with potential metabolic and natriuretic peptide effects were unevenly distributed across the groups, we examined the associations of GLP-1 receptor agonists (GLP-1 RA) and SGLT2 inhibitors (SGLT2i) use with fasting levels and postprandial changes of each peptide within the HR group, adjusted for age, sex and BMI (Supplementary Tables 11a-c). For the first research question, in which the HR group serves as reference, we additionally recalculated the main models with adjustment for SGLT2i use and, as a robustness analysis, for GLP-1 RA use. All sensitivity analyses are reported in the Supplementary Material (Tables 1d-l, 4d-f, 7d-l, 10 and 11a-c).

As recommended by the American Statistical Association [36], this exploratory study focused on effect size estimation with 95% confidence intervals, rather than on null-hypothesis significance testing. Reported p-values are provided for descriptive purposes only and were not adjusted for multiple testing. We note that the SEM approach does not eliminate the fundamental limitations of adjusting for a variable that may lie on the pathway between exposure and outcome. It makes the contribution of direct and indirect paths explicit instead of collapsing them into a single adjusted estimate. Because the temporal relationship prior to the index event or study inclusion is unknown, the observed associations warrant further investigation. Analyses were based on the BeLOVE data export of 4 July 2025 and were performed using R version 4.2.2. SEM analyses were done using the lavaan package [37, 38]. SEMs were estimated using maximum likelihood estimation with listwise deletion of cases with missing data (complete case analysis). The resulting analytic sample sizes for each model are provided in the figure legends. The number of eligible participants, complete cases and excluded participants for each model and group is given in Supplementary Table 14. As a sensitivity analysis, the main SEMs (all three exposures and all three peptides) were additionally estimated using full information maximum likelihood (FIML), which uses all available data of each participant, including those with partially missing values. The estimates of the exposure paths were nearly identical to those of the complete-case analysis (Supplementary Table 15), and the conclusions were unchanged. The reporting of this study adheres to the STROBE guidelines [39].

## Results

### Baseline characteristics

Between 18 July 2017 and 11 December 2020, a total of 1,897 individuals were enrolled in the BeLOVE study across five predefined clinical arms (high-risk diabetes, acute kidney injury, acute coronary syndrome, heart failure and stroke). Of these, 1,471 did not undergo the nutritional challenge at the deep phenotyping visit. A total of 426 participants completed the MMT. Of these, 33 were excluded due to limited sample size (see Section “Sample size and comparative groups”). Further exclusions were applied for incomplete MMT, deviations from the predefined postprandial sampling interval, non-fasting status and missing fasting information (Fig. 1). The final analytical sample included 373 participants from three study arms: 81 had experienced ACS, 186 had a history of acute cerebrovascular disorder and 106 belonged to the HR group. The baseline demographic and metabolic characteristics of these groups are presented in Table 2.

**Table 2:** Demographic and metabolic characteristics of the cohort under investigation

Baseline parameters [IQR]	HR group *n* = 106	ACS *n* = 81	Stroke *n* = 186
Age (y)	64 [58;69]	64 [55;74]	66 [56;77]
Male/ Female (%)	63%/37%	81%/19%	68%/32%
Days since index event	39 [31;46]*	105 [93;116]	102 [94;119]
T2D n (%)	106 (100)	26 (32)	32 (17)
Atrial fibrillation n (%)	19 (17.9)	14 (17.3)	46 (24.7)
w/o T2D	-	9 (16.4)	37 (24.2)
w T2D	19 (17.9)	5 (19.2)	9 (28.1)
Missing data	0	0	1
eGFR (mL/min/1.73 m²)	84.5 [69.2; 101.0]	81.0 [71.0; 95.0]	81.0 [69.0; 95.0]
w/o T2D	-	80.0 [71.0; 99.2]	82.0 [72.0; 96.0]
w T2D	84.5 [69.2; 101.0]	84.0 [68.0; 94.0]	71.5 [58.8; 88.8]
Missing data	6	4	5
Insulin treatment n (%)	51 (48.1)	4 (4.9)	7 (3.8)
w/o T2D	-	-	-
w T2D	51 (48.1)	4 (15.4)	7 (21.9)
Metformin treatment n (%)	68 (64.2)	6 (7.4)	18 (9.7)
w/o T2D	-	-	-
w T2D	68 (64.2)	6 (23.1)	18 (56.2)
SGLT2i treatment n (%)	29 (27.4)	1 (1.2)	6 (3.2)
w/o T2D	**-**	-	-
w T2D	29 (27.4)	1 (3.8)	6 (18.8)
GLP-1 RA treatment n (%)	23 (21.7)	0	0
w/o T2D	-	-	-
w T2D	23 (21.7)	0	0
BMI (kg/m²)	33.2 [28.7;37.3]	27.2 [25.1;31.0]	27.1 [23.8;29.8]
w/o T2D	-	27.0 [25.1;30.3]	26.4 [23.5;28.8]
w T2D	33.2 [28.7;37.3]	30.2 [26.0;32.9]	31.1 [28.2;35.0]
Waist circumference (cm)	113.5 [103.6;125.0]	102.3 [94.5; 111.3]	97.7 [90.3; 107.3]
w/o T2D	-	101.5 [93.5;106.0]	96.0 [88.0;104.0]
w T2D	113.5 [103.6;125.0]	106.0 [98.4;115.0]	110.2 [99.5;119.2]
Missing data	0	0	1
Fasting glucose (mg/dl)	137.0 [115.0;160.5]	98.0 [93.0;111.0]	93.0 [86.0;103.0]
w/o T2D	-	96.0 [91.0;100.0]	91.0 [85.0;98.0]
w T2D	137.0 [115.0;160.5]	125.0 [100.0;143.0]	117.5 [106.0;141.5]
Missing data	6	4	4
HbA1c (%)	6.8 [6.1;7.6]	5.8 [5.6;6.1]	5.6 [5.4;5.9]
w/o T2D	-	5.7 [5.5;5.9]	5.5 [5.3;5.7]
w T2D	6.8 [6.1;7.6]	6.3 [6.0;6.9]	6.5 [6.1;7.1]
Missing data	6	5	6
Fasting insulin (mU/l)	16.6 [9.1;24.2]	13.9 [9.9;19.6]	11.3 [7.7;16.3]
w/o T2D	-	13.3 [8.3;20.9]	10.7 [7.5;14.6]
w T2D	16.6 [9.1;24.2]	15.1 [12.2;18.3]	13.2 [10.7;30.1]
Missing data	7	4	5
HOMA-IR	5.5 [3.4;9.4]	3.4 [2.4;5.1]	2.7 [1.8;3.8]
w/o T2D	-	3.2 [2.0;4.5]	2.4 [1.7;3.4]
w T2D	5.5 [3.4;9.4]	4.4 [3.7;5.5]	4.8 [3.0;9.7]
Missing data	7	4	6
Fasting NT-proBNP (ng/l)	75 [30;130]	246 [83;422]	125 [51;317]
w/o T2D	-	242 [104;398]	112 [42;272]
w T2D	75 [30;130]	256 [62;892]	192 [122;490]
Missing data	7	4	4
Postprandial NT-proBNP (ng/l)	74 [30;128]	257 [86;455]	130 [51;329]
w/o T2D	-	257 [96;395]	113 [41;294]
w T2D	74 [30;128]	268 [66;741]	209 [144;511]
Missing data	7	4	9
Fasting MR-proANP (pmol/l)	67.0 [42.5;100.0]	126.0 [76.0;192.0]	109.5 [62.0;172.5]
w/o T2D	-	124.5 [70.2;177.8]	98.0 [59.5;169.8]
w T2D	67.0 [42.5;100.0]	126.0 [90.0;196.0]	136.5 [91.5;177.5]
Missing data	7	4	6
Postprandial MR-proANP (pmol/l)	62.0 [39.5;88.5]	107.0 [67.0;140.5]	92.0 [57.2;155.5]
w/o T2D	-	104.5 [64.5;138.8]	84.5 [53.8;157.8]
w T2D	62.0 [39.5;88.5]	112.0 [81.8;140.8]	113.0 [82.0;154.2]
Missing data	7	5	10
Fasting Copeptin (pmol/l)	7.6 [4.6;11.7]	8.2 [4.9;12.2]	6.4 [3.9;10.0]
w/o T2D	-	8.3 [5.0;12.4]	5.8 [3.6;9.1]
w T2D	7.6 [4.6;11.7]	7.2 [4.8;11.0]	9.4 [7.8;15.8]
Missing data	11	10	10
Postprandial Copeptin (pmol/l)	6.9 [4.4;13.2]	6.8 [4.9;10.4]	5.8 [3.8;8.3]
w/o T2D	-	6.8 [4.7;10.4]	5.1 [3.7;7.4]
w T2D	6.9 [4.4;13.2]	6.8 [5.2;10.1]	8.7 [6.1;13.5]
Missing data	7	5	10

Data are presented as median and interquartile range of selected parameters or absolute/relative frequencies

*Days since enrollment, as the HR group had no index event. ACS, acute coronary syndrome; HR, high risk; IQR, interquartile range; T2D, type 2 diabetes; w/o T2D, without type 2 diabetes; w T2D, with type 2 diabetes; eGFR, estimated glomerular filtration rate; BMI, body mass index; HbA1c, glycated hemoglobin A1c; HOMA-IR, homeostatic model assessment of insulin resistance; NT-proBNP, N-terminal pro-B-type natriuretic peptide; MR-proANP, mid-regional pro-atrial natriuretic peptide

All individuals in the HR group had type 2 diabetes, while the prevalence of diabetes was 32.1% in the ACS group and 17.2% in the stroke group. Not surprisingly, individuals with diabetes demonstrated numerically higher BMI and waist circumference compared to participants without diabetes. The median age was similar between all groups, while sex was not. Male participants were predominant in all three cohorts, particularly within the ACS group (81%).

Markers of glycemic control such as HbA1c and fasting glucose, and insulin resistance (fasting insulin levels, HOMA-IR) were elevated in participants with diabetes across disease entities, with numerically highest values in the HR cohort (Table 2). 48.1% of the HR group required insulin therapy, compared with 15.4% of the diabetes patients in the ACS group and 21.9% of the diabetes patients in the stroke group (Table 2).

Baseline MR-proANP and NT-proBNP were substantially higher in the acute cardiovascular event groups compared with the HR reference group. Fasting copeptin levels were highest in stroke patients with diabetes compared with both, stroke patients without diabetes and the HR group.

### Effects of meal challenge

All parameters related to glucose metabolism increased following consumption of the standardized test meal. An increase in blood glucose was observed in the HR group and in participants with diabetes in both event cohorts, whereas changes in the non-diabetic subgroups were smaller. The increase of glucose levels was accompanied by an increase in insulin postprandially in all groups. MR-proANP decreased consistently after the MMT across all groups. NT-proBNP showed little to no postprandial change, while copeptin showed a slight median decline with greater interindividual variability (Table 3). The corresponding individual-level distributions of the postprandial peptide changes are shown in Supplementary Fig. 6.

**Table 3:** Changes in metabolic parameters, natriuretic peptides and copeptin after the mixed meal challenge

Parameters (factor of change)	HR group *n* = 106	ACS *n* = 81	Stroke *n* = 186
Glucose	1.38 [1.21;1.59]	1.20 [1.03;1.39]	1.16 [1.00;1.42]
w/o T2D	-	1.14 [1.01;1.37]	1.14 [0.98;1.39]
w T2D	1.38 [1.21;1.59]	1.25 [1.09;1.43]	1.23 [1.08;1.48]
Insulin	3.12 [2.03;4.54]	5.46 [3.20;7.40]	4.80 [3.11;7.96]
w/o T2D	-	5.80 [3.61;7.29]	4.94 [3.15;8.46]
w T2D	3.12 [2.03;4.54]	4.57 [2.77;7.74]	3.85 [2.13;5.92]
MR-proANP	0.87 [0.80;0.96]	0.85 [0.78;0.97]	0.89 [0.80;0.96]
w/o T2D	-	0.84 [0.78;0.99]	0.89 [0.80;0.97]
w T2D	0.87 [0.80;0.96]	0.89 [0.78;0.97]	0.90 [0.80;0.94]
NT-proBNP	1.00 [0.91;1.08]	1.01 [0.93;1.11]	1.03 [0.96;1.12]
w/o T2D	-	1.00 [0.93;1.09]	1.02 [0.95;1.12]
w T2D	1.00 [0.91;1.08]	1.02 [0.92;1.11]	1.05 [0.99;1.09]
Copeptin	0.97 [0.84;1.19]	0.91 [0.75;1.11]	0.93 [0.79;1.04]
w/o T2D	-	0.90 [0.73;1.10]	0.93 [0.79;1.04]
w T2D	0.97 [0.84;1.19]	0.95 [0.82;1.13]	0.92 [0.84;1.02]

Data are presented as median and interquartile range of the postprandial response (postprandial/fasting ratio)

ACS, acute coronary syndrome; ANP, atrial natriuretic peptide; BNP, B-type natriuretic peptide; HR, high risk; IQR, interquartile range; MR-proANP, mid-regional pro-atrial natriuretic peptide; NT-proBNP, N-terminal pro-B-type natriuretic peptide; T2D, type 2 diabetes; w/o T2D, without type 2 diabetes; w T2D, with type 2 diabetes

### Cardiometabolic peptide responses in relation to CV disease and metabolic state

#### Link between acute CV events and cardiometabolic peptide response in participants with T2D

To investigate how disease entity and clinical-metabolic parameters are associated with fasting concentrations and postprandial responses of cardiometabolic peptides, we used the SEM framework in patients with T2D across all disease entities. Restricting this analysis to participants with T2D holds diabetes status constant across compared groups, so that the association of a recent acute CV event with fasting and postprandial peptide levels is not confounded by differences in diabetes prevalence.

**Fig. 2:**
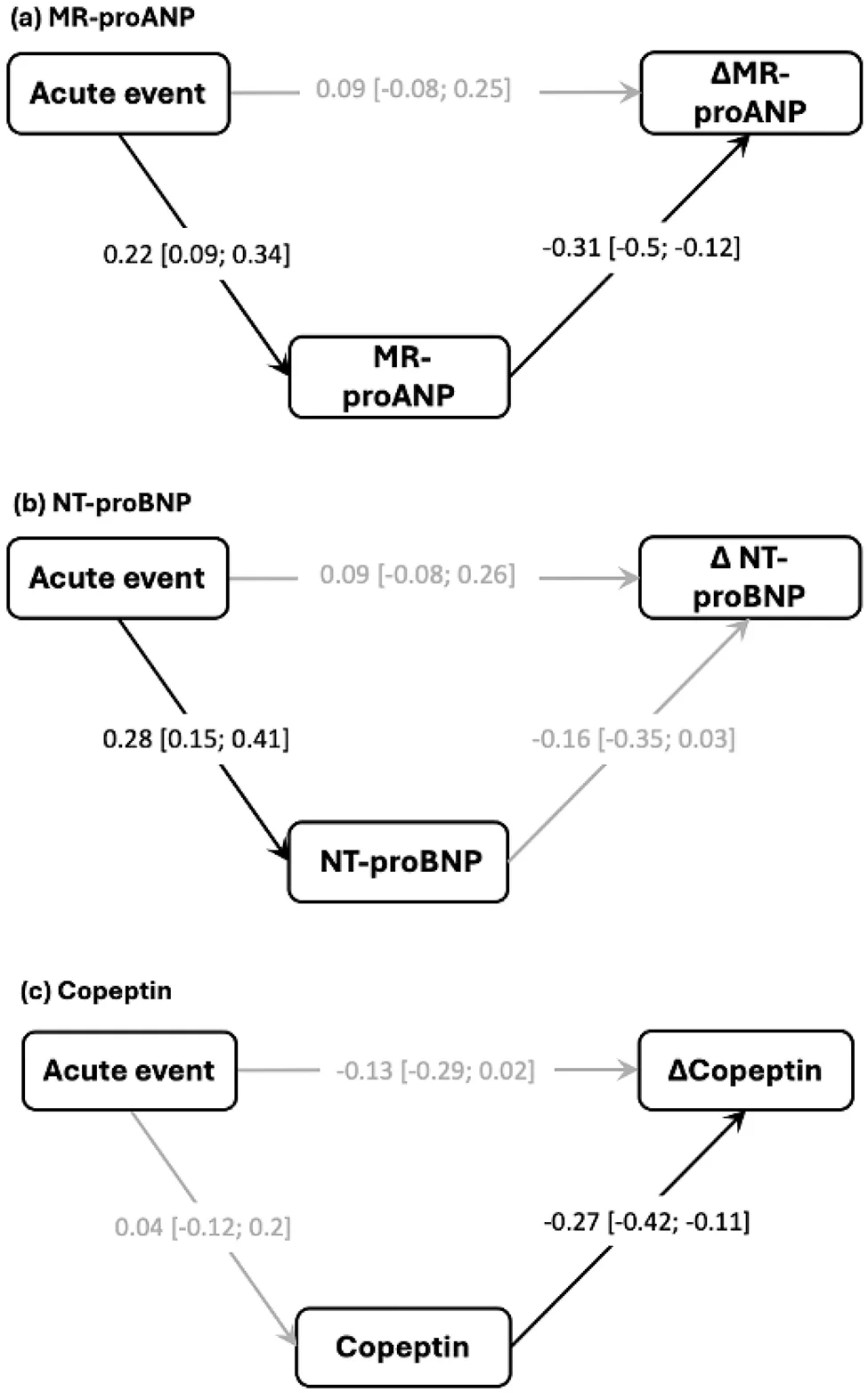
SEMs linking acute CV events to fasting and postprandial levels of (**a**) MR-proANP, (**b**) NT-proBNP and (**c**) copeptin. The reference category for the binary exposure variable was the high CV risk group without acute cardiovascular event. Group sizes per model: (**a**, **b**) *n* = 57 with acute CV event (stroke/ACS) and diabetes vs. *n* = 98 high CV risk group; (**c**) *n* = 56 vs. *n* = 94, respectively. Numbers at arrows indicate standardized regression coefficients with 95% CI. (**a**) R^2^ = 0.11 for ∆MR-proANP and R^2^ = 0.43 for fasting MR-proANP. (**b**) R^2^ = 0.07 for ∆NT-proBNP and R^2^ = 0.36 for NT-proBNP. (**c**) R^2^ = 0.16 for ∆copeptin and R^2^ = 0.15 for fasting copeptin. Fasting and postprandial levels were adjusted for age, sex and BMI. Full models, including unstandardized regression coefficients, are shown in the Supplements (Tables 1a-c)

A prior acute CV event was associated with higher fasting MR-proANP concentrations, and higher baseline levels were related to a more pronounced postprandial decline. However, there was no relevant direct link between prior CV event and the magnitude of postprandial MR-proANP decline (Fig. 2a).

Similarly, fasting NT-proBNP concentrations were elevated in participants with a history of acute CV event. However, the prior acute CV events showed no relevant association with postprandial changes. Fasting NT-proBNP levels indicated some negative association with ΔNT-proBNP (Fig. 2b).

For copeptin, we found no relevant associations between prior acute CV events and either fasting copeptin or its postprandial change. Only higher baseline levels were linked to a more pronounced decline of copeptin (Fig. 2c).

To examine whether pooling ACS and stroke masked entity-specific effects, we repeated the models separately in each subgroup (Supplementary Fig. 3, Supplementary Tables 2–3). For all three peptides, the associations were directionally similar to those in the pooled acute event group. The contrast estimates also provided no relevant indication of differences between the ACS and stroke groups (fasting MR-proANP: b=-0.002 [-0.276; 0.272], ΔMR-proANP: b=-0.016 [-0.083; 0.052], Supplementary Table 13). Including AF and eGFR as covariates in our SEMs also did not alter the main associations of interest (Supplementary Tables 1d-f). Additional adjustment for SGLT2i and GLP-1 RA use also did not materially change the associations of interest for any of the three peptides (Supplementary Tables 1 g-l).

#### Link between T2D and cardiometabolic peptide response in participants with acute CV event

All patients with acute CV events, both with and without T2D, were analyzed, so that the acute event was held constant across the compared groups. T2D was not associated with differences in fasting or postprandial changes of MR-proANP (Supplementary Fig. 2a) or NT-proBNP (Supplementary Fig. 2b). The only consistent relationship was found between fasting and postprandial peptide levels, where higher baseline concentrations were related to larger declines, particularly for MR-proANP.

Repeating the models separately for ACS and stroke (Supplementary Fig. 4, Supplementary Tables 5–6) showed similar patterns for both cohorts. For both natriuretic peptides T2D was not associated with either their fasting or postprandial levels.

For copeptin, the stratified analysis suggested a possible cohort-specific pattern. In the stroke subgroup, T2D was associated with a slightly higher fasting copeptin, which in turn was related to a stronger postprandial decline. There was no relevant direct association between T2D and the postprandial change. The corresponding fasting estimate in the ACS subgroup was small, in the opposite direction and more uncertain than in patients with stroke (Supplementary Fig. 4c). No relevant association between T2D and fasting or postprandial copeptin was found in the pooled cohort (Supplementary Fig. 2c). However, the subgroup CIs overlapped substantially, and the T2D subgroups were small, so this apparent difference between the ACS and stroke subgroups is uncertain.

Adjusting the models for AF and renal function as additional covariates did not materially change the observed associations (Supplementary Tables 4d-f).

#### Link between insulin resistance and cardiometabolic peptide response in participants with acute CV event

**Fig. 3:**
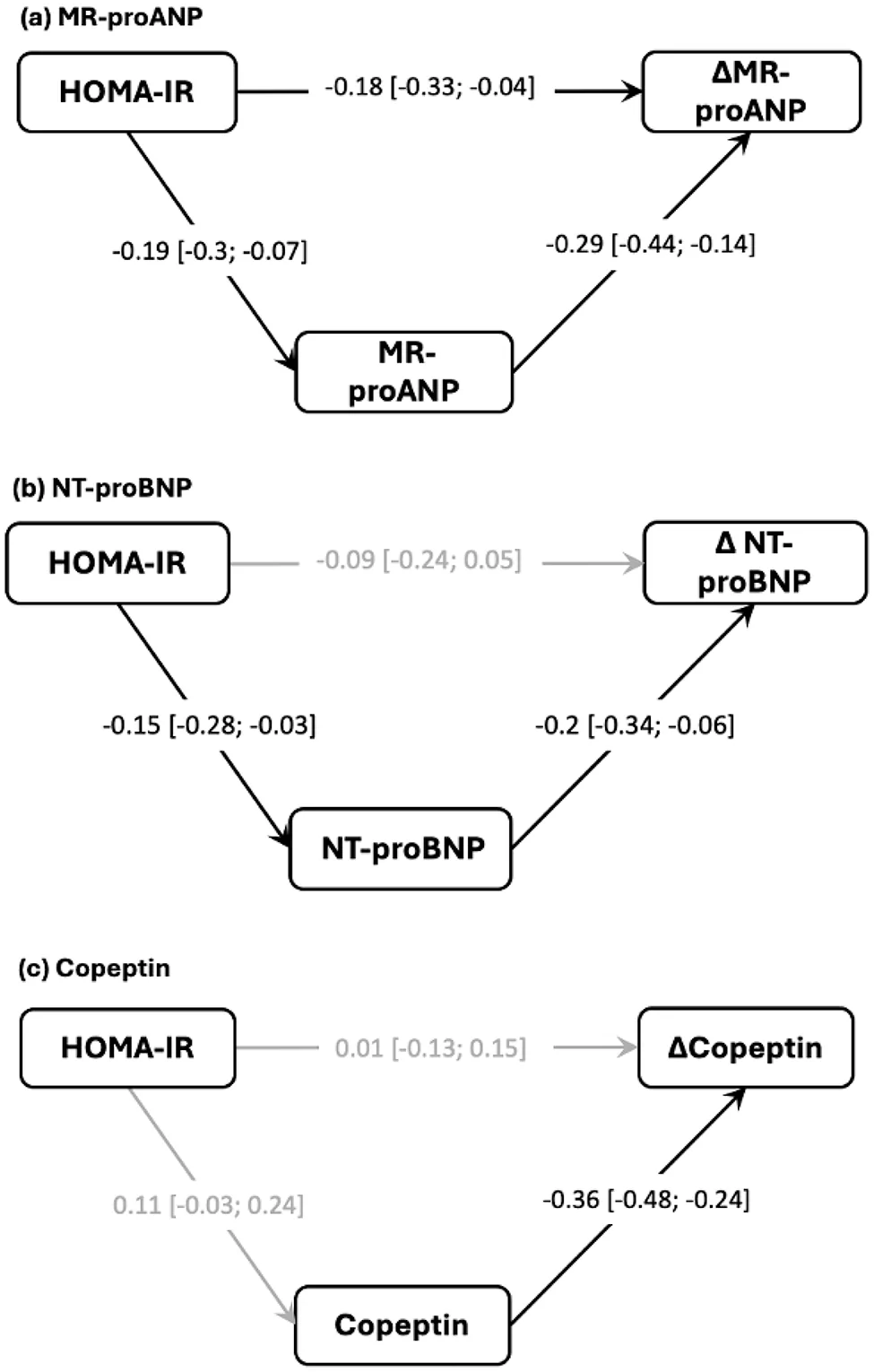
SEMs linking HOMA-IR to fasting and postprandial levels of (**a**) MR-proANP, (**b**) NT-proBNP and (**c**) copeptin. Numbers at arrows indicate standardized regression coefficients with 95% CI. (**a**) *n* = 245; R^2^ = 0.07 for ∆MR-proANP and R^2^ = 0.39 for fasting MR-proANP. (**b**) *n* = 248; R^2^ = 0.10 for ∆NT-proBNP and R^2^ = 0.27 for NT-proBNP. (**c**) *n* = 235; R^2^ = 0.13 for ∆copeptin and R^2^ = 0.16 for fasting copeptin. Fasting and postprandial levels were adjusted for age, sex and BMI. Full models, including unstandardized regression coefficients, are shown in the Supplements (Tables 7a-c)

Given the effect of acute events on changes of cardiometabolic peptides, we analyzed the relationship between insulin resistance and changes in cardiometabolic peptides in all participants with a recent acute CV event using SEMs. This was in line with previous analyses regarding the effect of T2D. We revealed that higher HOMA-IR was associated with lower fasting MR-proANP and a stronger postprandial decline of MR-proANP (Fig. 3a).

For NT-proBNP, a similar but weaker pattern was observed. Higher HOMA-IR values were associated with lower fasting concentrations of NT-proBNP, but the direct association between HOMA-IR and postprandial NT-proBNP change was minor (Fig. 3b).

In contrast, neither fasting nor postprandial copeptin levels showed any relevant direct association with HOMA-IR (Fig. 3c).

Repeating the SEMs separately for stroke and ACS (Supplementary Fig. 5, Supplementary Tables 8–9) showed the same overall pattern in both cohorts: higher HOMA-IR was associated with lower fasting MR-proANP and a stronger postprandial decline. Although the association appeared somewhat more pronounced on the fasting path in the stroke cohort and on the postprandial path in the ACS cohort (Supplementary Fig. 5a), the contrast estimates for the HOMA-IR × event type interaction did not indicate relevant differences between the two entities (fasting MR-proANP b = 0.085 [-0.106; 0.277], ΔMR-proANP b=-0.014 [-0.084; 0.056], Supplementary Table 13). The key inverse association between HOMA-IR and MR-proANP was therefore consistent across both subgroups.

The results remained similar after additional adjustment for AF and eGFR as potential confounders (Supplementary Tables 7d-f). Excluding the 11 participants receiving insulin therapy, in whom HOMA-IR may be distorted by exogenous insulin, left the associations essentially unchanged (fasting MR-proANP: β=-0.16 [-0.28; -0.05]; ΔMR-proANP: β=-0.17 [-0.31; -0.02]; fasting NT-proBNP: β=-0.12 [-0.24; 0.01]; ΔNT-proBNP: β=-0.10 [-0.24; 0.04]; fasting copeptin: β = 0.03 [-0.11; 0.17]; ΔCopeptin: β = 0.00 [-0.14; 0.14]; Supplementary Tables 7 g-i). Additional adjustment for SGLT2i use (*n* = 7 in this cohort) likewise left the associations essentially unchanged (Supplementary Tables 7j-l).

**Table 4:** Consolidated SEM estimates

Exposure	Peptide	Path	b [95% CI]	b* [95% CI]	*R*²
Acute CV event	MR-proANP	→ fasting	0.31 [0.13; 0.49]	0.22 [0.09; 0.34]	0.43
		→ Δ	0.02 [-0.02; 0.07]	0.09 [-0.08; 0.25]	0.11
		fasting → Δ	-0.06 [-0.10; -0.02]	-0.31 [-0.50; -0.12]	
	NT-proBNP	→ fasting	0.80 [0.42; 1.19]	0.28 [0.15; 0.41]	0.36
		→ Δ	0.03 [-0.02; 0.07]	0.09 [-0.08; 0.26]	0.07
		fasting → Δ	-0.02 [-0.03; 0.00]	-0.16 [-0.35; 0.03]	
	Copeptin	→ fasting	0.06 [-0.16; 0.28]	0.04 [-0.12; 0.20]	0.15
		→ Δ	-0.09 [-0.20; 0.02]	-0.13 [-0.29; 0.02]	0.16
		fasting → Δ	-0.13 [-0.21; -0.05]	-0.27 [-0.42; -0.11]	
T2D	MR-proANP	→ fasting	-0.03 [-0.20; 0.14]	-0.02 [-0.13; 0.09]	0.37
		→ Δ	-0.00 [-0.06; 0.06]	-0.00 [-0.14; 0.14]	0.05
		fasting → Δ	-0.07 [-0.12; -0.03]	-0.26 [-0.41; -0.11]	
	NT-proBNP	→ fasting	0.25 [-0.12; 0.63]	0.08 [-0.04; 0.20]	0.26
		→ Δ	-0.02 [-0.06; 0.03]	-0.05 [-0.18; 0.08]	0.1
		fasting → Δ	-0.02 [-0.03; -0.00]	-0.18 [-0.32; -0.05]	
	Copeptin	→ fasting	0.12 [-0.10; 0.33]	0.07 [-0.06; 0.20]	0.16
		→ Δ	0.04 [-0.06; 0.14]	0.06 [-0.08; 0.19]	0.13
		fasting → Δ	-0.16 [-0.22; -0.10]	-0.36 [-0.48; -0.24]	
HOMA-IR	MR-proANP	→ fasting	-0.17 [-0.28; -0.06]	-0.19 [-0.30; -0.07]	0.39
		→ Δ	-0.05 [-0.09; -0.01]	-0.18 [-0.33; -0.04]	0.07
		fasting → Δ	-0.09 [-0.13; -0.04]	-0.29 [-0.44; -0.14]	
	NT-proBNP	→ fasting	-0.29 [-0.53; -0.05]	-0.15 [-0.28; -0.03]	0.27
		→ Δ	-0.02 [-0.05; 0.01]	-0.09 [-0.24; 0.05]	0.1
		fasting → Δ	-0.02 [-0.03; -0.01]	-0.20 [-0.34; -0.06]	
	Copeptin	→ fasting	0.11 [-0.04; 0.25]	0.11 [-0.03; 0.24]	0.16
		→ Δ	0.00 [-0.06; 0.07]	0.01 [-0.13; 0.15]	0.13
		fasting → Δ	-0.16 [-0.22; -0.10]	-0.36 [-0.48; -0.24]	

Associations of acute CV event, T2D and insulin resistance with fasting concentrations and postprandial changes of cardiometabolic peptides. Unstandardized (b) and standardized (b*) coefficients with 95% CI from the pooled SEMs. Fasting refers to the natural log-transformed fasting concentration; ∆*Peptide* refers to the change in log-transformed concentrations (ln(postprandial) - ln(fasting)). Each outcome (fasting peptides, ∆ of peptides) was adjusted for age, sex and BMI. R² is given for the fasting and Δ outcome of each model

## Discussion

Our study provides new insights into the postprandial dynamics of cardiometabolic peptides in a deeply phenotyped cohort of participants with high cardiovascular risk. By assessing responses of MR-proANP, NT-proBNP, and copeptin to a standardized study-specific mixed meal test, we observed a postprandial suppression of MR-proANP and copeptin after a fat-enriched meal, while NT-proBNP seems not to be substantially affected by meal intake. Across all three peptides, postprandial concentrations were primarily associated with fasting peptide concentrations, with no relevant association observed for acute CV events or T2D. Notably, postprandial MR-proANP levels were additionally associated with the continuous degree of IR rather than with the categorical diagnosis of diabetes per se.

Based on experimental evidence that ANP can stimulate adipocyte triglyceride hydrolysis via the NPR-A/cGMP/PKG pathway, a metabolic effect largely absent for BNP [3, 40, 41], the postprandial decline of MR-proANP may potentially reflect reduced ANP-mediated lipolytic signaling after food intake. One possible explanation is that this pattern may be related to the well-described postprandial shift in substrate utilization [24]. However, ANP bioactivity, lipolysis and substrate oxidation were not assessed in the present study. Therefore, a mechanistic link between the observed MR-proANP changes and postprandial substrate utilization cannot be established from our data. Prior acute CV event was associated with higher fasting MR-proANP and NT-proBNP, but not with the magnitude of their postprandial change once fasting levels were accounted for. Within the acute event groups, neither fasting concentrations nor the postprandial responses of MR-proANP and NT-proBNP varied with the time interval between index event and the deep phenotyping visit (Supplementary Table 10), indicating that the preserved postprandial decline in MR-proANP was probably not driven by the timing of assessment. This suggests that nutritional state may override acute post-ischemic alterations in short-term NP regulation. These findings do not support a relevant impact of acute cardiovascular events on the postprandial regulation of these peptides.

Recent acute CV events were associated with higher fasting levels of both natriuretic peptides, but not substantially with their postprandial responses. Elevated fasting MR-proANP after ACS or stroke may reflect greater hemodynamic stress. Experimental studies suggest that natriuretic peptides can influence adipose tissue lipolysis and lipid availability, providing additional fatty acid substrates for myocardial energy metabolism [24, 42, 43], but our study did not assess whether the observed peptide concentrations were related to systemic lipid utilization. However, there is currently no direct evidence from human studies linking increased ANP to cardiac lipid utilization after ischemic injury. Animal data suggest that natriuretic peptide infusion can increase cardiac triglyceride content after ischemia-reperfusion, but this has not been confirmed in humans [44]. Additionally, elevations in peptides are accompanied by rises in plasma non-esterified fatty acids, which have been associated with impaired outcomes such as arrhythmias and oxidative injury [45–47]. Thus, whether this is potentially beneficial under emergency energy demand or harmful under conditions of lipid overload remains unclear and cannot be assessed in the present study.

In contrast to the minimal relationship between acute CV events and postprandial NP responses, higher HOMA-IR was associated not only with lower fasting MR-proANP but also with a stronger postprandial suppression, even in participants with well-controlled diabetes. This difference also makes biological sense. Insulin resistance is a continuous trait, while the presence of type 2 diabetes categorizes a wide range of metabolic states into a binary category. The continuous measure can capture the variation that the binary label might overlook. Prior studies have shown that hyperinsulinemia increases the NPR-C in adipose tissue [7], so circulating MR-proANP might reflect the level of insulin resistance, as shown by HOMA-IR, more closely than the diabetes label. In our cohort, this difference was even clearer because the diabetic participants were mostly well-controlled: their glycemic control was stable while the underlying insulin resistance remained, which might have reduced the metabolic information from the categorical diagnosis.

The lower fasting levels may reflect a reduced ANP-mediated lipolytic capacity, which was not assessed in the present study. While postprandial MR-proANP suppression has been linked to a shift toward carbohydrate oxidation [24], the combination of low fasting levels and a stronger postprandial drop is more consistent with metabolic dysregulation rather than with a preserved adaptive capacity. As this pattern was associated with the continuous degree of IR, impaired MR-proANP regulation may already show in earlier, potentially more modifiable stages of metabolic dysfunction, before overt diabetes develops. This may be consistent with evidence that ANP-system activity relates to changes in insulin sensitivity and fat mass during weight loss [10]. Whether improvements in insulin sensitivity are accompanied by recovery of postprandial MR-proANP regulation cannot be addressed by the present cross-sectional design and requires prospective interventional study. Further studies are as well required to address the mechanisms underlying the stronger postprandial suppression in participants with higher IR.

The limited postprandial responsiveness of NT-proBNP, in contrast to the pronounced suppression of MR-proANP, may be explained by two complementary factors. First, the metabolic actions of the natriuretic peptide system, including lipolysis, are mediated mainly by ANP acting on NPR-A receptors in adipose and skeletal muscle tissue [3, 40, 41]. The B-type pathway plays a much smaller role in this metabolic signaling, so the meal-induced change observed here likely reflects parts of the metabolic response. Second, the two peptide systems are released in response to different signals. ANP responds to acute atrial and metabolic stimuli, whereas BNP release is driven mainly by ventricular wall stress, which changes rather slowly and is not affected by a single meal [3, 15]. These properties are consistent with the minimal and inconsistent postprandial NT-proBNP changes observed in our cohort and in prior studies [16–18] and indicate that NT-proBNP primarily reflects chronic cardiac loading rather than acute nutrient-driven regulation.

In contrast to the NPs, neither IR, T2D, nor prior acute CV events were substantially associated with fasting copeptin or its postprandial change. The only consistent relationship was between fasting copeptin and its own postprandial response, where higher baseline concentrations were linked to a larger subsequent decline. Importantly, the postprandial reduction observed across our cohort cannot be attributed specifically to the macronutrient composition of the meal. The liquid test meal itself delivered 300 mL of water and small amounts of additional water were permitted during the test, so transient suppression of vasopressin release secondary to fluid ingestion and changes in plasma osmolality may have contributed. This is in line with other studies demonstrating that oral fluid intake, even in small amounts, can significantly decrease copeptin levels, while food intake without accompanying fluid does not show a substantial effect [20, 48]. Consistent with this non-specific regulation, the postprandial copeptin response was the only outcome weakly associated with the interval between index event and the deep phenotyping visit, which we do not regard as evidence of a time-dependent recovery of vasopressin regulation after the acute event (Supplementary Table 10). As additional water intake during the test was not explicitly documented, its contribution to inter-individual variability in the copeptin response cannot be quantified.

Methodologically, the explained variance in postprandial peptide changes was modest across the main models (R² ranging from 0.05 to 0.16), which is not unexpected given the complexity of postprandial regulation in clinically heterogeneous, high-risk cohorts. The explained variance was higher for the fasting concentrations (R² 0.15–0.43) than for the postprandial change, consistent with a within-person difference carrying more short-term variability than the fasting level itself. Accordingly, these models characterize average associations with metabolic status but have limited ability to explain individual postprandial responses.

## Strengths and limitations

In this study, we used a standardized, study-specific mixed-meal test, developed to better reflect typical Western dietary patterns with a higher proportion of fat compared to conventional carbohydrate-rich test meals. The integration of detailed clinical and metabolic phenotyping, together with the direct comparison of ACS and stroke subgroups as well as diabetic vs. non-diabetic individuals, provides an overview of cardiometabolic regulation across different contexts. While our study design points out limitations of static fasting assessments and demonstrates the feasibility of standardized postprandial phenotyping in high-risk patients, the value of dynamic peptide responses as potential risk markers requires further validation. The planned longitudinal follow-up within BeLOVE will be critical to determine whether postprandial peptide responses provide prognostic information for cardiovascular outcomes and can inform therapeutic strategies [49–52].

However, several limitations should also be considered. First, the study population is characterized by high cardiovascular risk and predominance of well-controlled diabetes (median HbA1c 6.3% − 6.8%), which may limit generalizability to broader or less well-managed populations. The relatively low glycemic variability within this cohort could weaken observed associations between diabetes status and peptide responses, potentially underestimating the impact of poorly controlled diabetes. Second, participants included in the present analysis were required to have completed the MMT after the index event. This may have introduced a selection bias toward clinically more stable individuals, as participants with more severe acute presentations or greater frailty may have been less likely to attend or complete the deep phenotyping visit. Consistent with this, the stroke cohort was characterized by predominantly mild neurological deficits (Supplementary Table 12). Consequently, our findings may not fully represent cardiometabolic peptide dynamics in more severely ill patients. Third, although the overall sample size is substantial, subgroup analyses (by acute event type and diabetes status) involved smaller sample sizes, resulting in less precise estimates, and these results should be interpreted with caution. Findings in the pooled acute event group should therefore not be assumed to apply equally to ACS and stroke. Insulin resistance was estimated using HOMA-IR, which reflects fasting rather than postprandial insulin sensitivity. Dynamic measures such as a clamp or oral glucose load were not part of the phenotyping protocol. Our findings accordingly apply to insulin-based estimation of fasting insulin resistance. Additionally, a minority of participants in the acute event groups were receiving insulin therapy at the time of the mixed meal test (11 out of 267 participants), which may affect HOMA-IR estimates due to exogenous insulin elevating fasting insulin concentrations. A sensitivity analysis indicated that excluding patients with insulin therapy did not materially change the observed associations (Supplementary Tables 7g-i). Two remaining glucose-lowering medications need some consideration, as SGLT2i and GLP-1 RA have both been associated with reductions in NT-proBNP [53–56], whereas evidence for a direct effect on ANP in humans is limited [57]. In our cohort, these medications were used almost exclusively by participants in the HR group, whereas their use in the acute event group was very limited or even absent (Table 2). Thus, potential confounding due to medication is relevant primarily for the first research question, in which the HR group serves as reference. Additional adjustment for SGLT2i use did not materially change the associations of interest, neither in the acute CV event models (Supplementary Tables 1g-i), nor in the HOMA-IR models (Supplementary Tables 7j-l). GLP-1 RA use was confined to the HR group, where GLP-1 RA use was associated with a more negative postprandial MR-proANP change (Supplementary Tables 11a-c). As GLP-1 RA use occurs exclusively within the HR reference group, it is collinear with group membership and cannot be treated as a conventional confounder. We nevertheless repeated the models with additional adjustment for GLP-1 RA use as a robustness check, which left the associations of interest essentially unchanged (Supplementary Tables 1j-l). A residual influence of GLP-1 RA use within the HR group can therefore not be excluded. Metformin, although used more frequently, was not modelled, as randomized trials found no effect on NT-proBNP [58, 59] and to our knowledge, no data are available for MR-proANP or copeptin. Additionally, residual confounding by the higher metabolic burden and more frequent insulin use in the HR group may have contributed in part to the differences in fasting natriuretic peptide levels between the acute event and HR groups for the first research question.

Fourth, postprandial peptide responses were measured at a single 120-minute time point. While this defines the net direction and magnitude of the response, it cannot resolve the full postprandial trajectory. However, previous data from our group on MR-proANP trajectories in obesity indicate a continuous postprandial decline with an almost maximal response after 120 min [10]. Denser, multi-point sampling will be required to characterize the complete postprandial dynamics and to establish whether such features carry additional prognostic information. Moreover, as a single standardized meal was used, our design cannot dissociate the contribution of individual macronutrients, in particular the fat-enriched composition of the BeLOVE test meal, from the response to a mixed meal in general. Prospective studies with comparator meals of differing macronutrient composition would be required to address this. The specific macronutrient composition of our protocol was used to address at least in part the increasing fat consumption observed in Germany [29, 30]. Fifth, our observational design does not allow for a causal inference, restricting the interpretation to hypothesis-generating associations. In addition, the pronounced imbalance in sex distribution in the acute event groups did not allow a reliable characterization of sex-specific associations. Sex-specific regulation of postprandial peptide responses will need to be addressed in dedicated, larger studies. Finally, the prognostic relevance of postprandial peptide changes remains to be established and requires longitudinal analyses with careful adjustment for baseline comorbidities.

In summary, this exploratory study identified distinct postprandial dynamics for MR-proANP, NT-proBNP, and copeptin in participants with high cardiovascular risk. MR-proANP emerged as the most metabolically responsive peptide, with insulin resistance rather than diabetes status as a key correlate of its regulation, whereas NT-proBNP remained largely insensitive to short-term metabolic stimuli. In contrast, copeptin dynamics appeared independent of metabolic or cardiovascular status and may reflect individual fluid- or osmoregulatory effects rather than nutrient-driven mechanisms. Together, these findings highlight the complex interplay between metabolic status and cardiac function and support the need for future longitudinal analyses to determine whether dynamic hormone responses can serve as prognostic markers and therapeutic targets in cardiovascular diseases.

## Supplementary Information

Below is the link to the electronic supplementary material.

Supplementary Material 1

## Data Availability

Data from the BeLOVE study are available upon reasonable request. Researchers may apply for access through the study’s Use and Access Committee, with the involvement of a collaborator from the BIH research community. Requests are evaluated for scientific merit and feasibility. Data sharing is conducted in accordance with General Data Protection Regulation (GDPR) requirements.

## Declarations

### Study registration

Approved WHO primary register: German Clinical Trials Register: https://drks.de/search/de/trial/DRKS00016852; WHO International Clinical Registry Platform http://apps.who.int/trialsearch/Trial2.aspx?TrialID=DRKS00016852.

## Abbreviations

ACS: Acute Coronary Syndrome
AF: Atrial Fibrillation
AKI: Acute Kidney Injury
ANP: Atrial Natriuretic Peptide
BMI: Body Mass Index
BNP: B-type Natriuretic Peptide
CI: Confidence Interval
CV: Cardiovascular
CVD: Cardiovascular Disease
eGFR: Estimated Glomerular Filtration Rate
HOMA-IR: Homeostatic Model Assessment of Insulin Resistance
HR: High Risk (reference group)
MMT: Mixed Meal Test
MR-proANP: Mid-Regional Pro-Atrial Natriuretic Peptide
NT-proBNP: N-Terminal pro-B-Type Natriuretic Peptide
NP: Natriuretic Peptide
NPR-A: Natriuretic Peptide Receptor A
NPR-C: Natriuretic Peptide Clearance Receptor
SEM: Structural Equation Model
T2D: Type 2 Diabetes
TIA: Transient Ischemic Attack
