# Supplementary material for: Meal-induced changes in cardiometabolic peptides in the context of acute cardiovascular diseases and glucose metabolism
Source: Cardiovasc Diabetol. 2026 Oct 26;25:314. doi: 10.1186/s12933-026-03424-5 (PMC13711810; doi:10.1186/s12933-026-03424-5)
Supplement: Supplementary file 1 [file 12933_2026_3424_MOESM1_ESM.docx]

**Supplementary material**

**Meal-induced changes in cardiometabolic peptides in the context of acute cardiovascular diseases and glucose metabolism**

Lishkova G^1,2,3^, Schweizerhof O^4,5^, Großmann J^1^, von Rohr K^1,3^, Landmesser U^2,4,6^, Edelmann F^2,6,7^, Eckardt KU^8^, Endres M^2,9,10,11,12^, Weber JE^2,4,9,10^, Grittner U^5^, Pischon T^13,14^, Schulz-Menger J^2,6,7^, Müller DN^2,13,15^, Spranger J^7^, Li L^1,3,16^, Mai K^1,2,3,16,17^*

on behalf of the BeLOVE Study Group

1. Department of Endocrinology and Metabolism, Charité-Universitätsmedizin Berlin, corporate member of the Freie Universität Berlin and Humboldt-Universität Berlin, ENDO-ERN, Berlin, Germany
2. German Centre for Cardiovascular Research (DZHK), Partner Site Berlin, Germany
3. German Center for Diabetes Research (DZD), München-Neuherberg, Germany
4. Berlin Institute of Health at Charité - Universitätsmedizin Berlin, Berlin, Germany
5. Institute of Biometry and Clinical Epidemiology, Charité-Universitätsmedizin Berlin, corporate member of the Freie Universität Berlin and Humboldt-Universität Berlin, Berlin, Germany
6. Deutsches Herzzentrum der Charité (DHZC), Department of Cardiology, Angiology and Intensive Care Medicine, Campus Virchow Klinikum, Berlin, Germany
7. Charité-Universitätsmedizin Berlin, corporate member of Freie Universität Berlin and

Humboldt-Universität Berlin, Berlin, Germany

8. Department of Nephrology and Medical Intensive Care, Charité-Universitätsmedizin Berlin, corporate member of the Freie Universität Berlin and Humboldt-Universität Berlin, Germany
9. Department of Neurology with experimental Neurology, Charité-Universitätsmedizin Berlin, corporate member of the Freie Universität Berlin and Humboldt-Universität Berlin, Berlin, Germany
10. Center for Stroke Research (CSB), Charité-Universitätsmedizin Berlin, corporate member of the Freie Universität Berlin and Humboldt-Universität Berlin, Berlin, Germany
11. German Center for Neurodegenerative Diseases (DZNE), partner site Berlin, Germany
12. German Center for Mental Health (DZPG), partner site Berlin, Germany
13. Max-Delbrück-Center for Molecular Medicine in the Helmholtz Association (MDC), Berlin, Germany
14. Biobank Technology Platform, Max Delbrück Center for Molecular Medicine in the Helmholtz Association (MDC), Berlin, Germany
15. Experimental and Clinical Research Center, a cooperation of Charité-Universitätsmedizin Berlin and Max Delbrück Center for Molecular Medicine, 13125 Berlin, Germany
16. Department of Human Nutrition, German Institute of Human Nutrition Potsdam-Rehbrücke, Nuthetal, Germany
17. Max Rubner Center for Cardiovascular Metabolic Renal Research, Charité-Universitätsmedizin Berlin, corporate member of the Freie Universität Berlin and Humboldt-Universität Berlin, Berlin, Germany

* Corresponding author: Prof. Knut Mai, MD

Charité – Universitätsmedizin Berlin

Department of Endocrinology and Metabolism,

Charitéplatz 1

10117 Berlin

Deutschland

E-Mail: knut.mai@charite.de

ORCID: 0000-0003-0126-3155

**Laboratory analysis - Measurement of insulin, MR-proANP, NT-proBNP, and Copeptin**

Serum insulin was measured using fluoroimmunometric assay (AutoDelfia; Perkin Elmer, Rodgau, Germany) (inter-assay coefficient of variation (CV) 2.3 - 3.5%, intra-assay CV 1.7 - 2.4%). Mid-region pro ANP was analyzed using a TRACE (time-resolved amplified cryptate emission) technology (BRAHMS MR-pro ANPKRYPTOR; BRAHMS ThermoFisher) (inter-assay CV ≤ 6.5%, intra-assay CV < 2.5%). NT-proBNP was analyzed by electrochemiluminescence immunoassay (ECLIA) on a Roche Cobas e601 platform (Roche Diagnostics) (inter-assay CV 1.7 - 3.1%, intra-assay CV 1.6 - 4.6%). Copeptin was measured with a TRACE technology (BRAHMS Copeptin proAVP KRYPTOR assay, Kryptor Compact Plus) (inter-assay CV < 18%, intra-assay CV < 15%). All assays were performed at Labor Berlin - Charité Vivantes GmbH according to manufacturer protocols. Measurements were done in a single batch on frozen samples (-80 °C) with internal quality controls within certified limits.

**Supplementary Figure legend**

**Supplementary Figure 1.** Conceptual SEM of direct and indirect associations between clinical exposure (acute CV event, T2D, HOMA-IR) and postprandial peptide change.

**Supplementary Figure 2.** SEMs linking T2D to fasting and postprandial levels of (a) MR-proANP, (b) NT-proBNP and (c) copeptin: combined model.

**Supplementary Figure 3.** SEMs linking acute CV events to fasting and postprandial levels of (a) MR-proANP, (b) NT-proBNP and (c) Copeptin: subanalysis of stroke and ACS subgroups.

**Supplementary Figure 4.** SEMs linking T2D to fasting and postprandial levels of (a) MR-proANP, (b) NT-proBNP and (c) Copeptin: subanalysis of stroke and ACS subgroups.

**Supplementary Figure 5:** SEMs linking HOMA-IR to fasting and postprandial levels of (a) MR-proANP, (b) NT-proBNP and (c) Copeptin: subanalysis of stroke and ACS subgroups.

**Supplementary Figure 6:** Individual-level distributions of postprandial peptide changes for (a) MR-proANP, (b) NT-proBNP and (c) Copeptin.

**Supplementary Figure 1**

**Supplementary Figure 1.** Conceptual SEM of direct and indirect associations between clinical exposure (acute CV event, T2D, HOMA-IR) and postprandial peptide change.

**Supplementary Figure 2**

**Supplementary Figure 2.** SEMs linking T2D to fasting and postprandial levels of (a) MR-proANP, (b) NT-proBNP and (c) copeptin: combined model. Numbers at arrows indicate standardized regression coefficients with 95% CI. (a) n=189 without T2D vs. n=57 with T2D; R^2^=0.05 for ∆MR-proANP and R^2^=0.37 for fasting MR-proANP. (b) n=192 vs. n=57; R^2^=0.10 for ∆NT-proBNP and R^2^=0.26 for NT-proBNP. (c) n=180 vs. n=56; R^2^=0.13 for ∆copeptin and R^2^=0.16 for fasting copeptin. Fasting and postprandial levels were adjusted for age, sex and BMI. Full models, including unstandardized regression coefficients, are shown in Supplementary Tables 4a-c.

**Supplementary Figure 3**

**Supplementary Figure 3.** SEMs linking acute CV events to fasting and postprandial levels of (a) MR-proANP, (b) NT-proBNP and (c) Copeptin: subanalysis of stroke and ACS subgroups. Numbers at arrows indicate standardized regression coefficients with 95% CI. (a) Stroke n=32/ACS n=25 vs. HR n=98. (b) Stroke n=32/ACS n=25 vs. HR n=98. (c) Stroke n=32/ACS n=24 vs. HR n=94.Fasting and postprandial levels were adjusted for age, sex and BMI. Full models, including unstandardized regression coefficients, are shown in Supplementary Table 2 (stroke) and Supplementary Table 3 (ACS).

**Supplementary Figure 4**

**Supplementary Figure 4.** SEMs linking T2D to fasting and postprandial levels of (a) MR-proANP, (b) NT-proBNP and (c) Copeptin: subanalysis of stroke and ACS subgroups. Numbers at arrows indicate standardized regression coefficients with 95% CI. (a) Stroke n=141 without T2D vs. n=32 with T2D, ACS n=48 without T2D vs. n=25 with T2D. (b) Stroke n=143 without T2D vs. n=32 with T2D, ACS n=49 without T2D vs. n=25 with T2D. (c) Stroke n=137 without T2D vs. n=32 with T2D, ACS n=43 without T2D vs. n=24 with T2D. Fasting and postprandial levels were adjusted for age, sex and BMI. Full models, including unstandardized regression coefficients, are shown in Supplementary Table 5 (stroke) and Supplementary Table 6 (ACS).

**Supplementary Figure 5**

**Supplementary Figure 5.** SEMs linking HOMA-IR to fasting and postprandial levels of (a) MR-proANP, (b) NT-proBNP and (c) Copeptin: subanalysis of stroke and ACS subgroups. Numbers at arrows indicate standardized regression coefficients with 95% CI. (a) n=172 Stroke vs. n=73 ACS. (b) n=174 Stroke vs. n=74 ACS. (c) n=168 Stroke vs. n=67 ACS. Fasting and postprandial levels were adjusted for age, sex and BMI. Full models, including unstandardized regression coefficients, are shown in Supplementary Table 8 (Stroke ) and Supplementary Table 9 (ACS).

**Supplementary Figure 6**

**Supplementary Figure 6.** Individual-level distributions of postprandial peptide changes for (a) MR-proANP, (b) NT-proBNP and (c) Copeptin, expressed as the difference of log-transformed concentrations (ln(postprandial) − ln(fasting)), stratified by study arm. Boxes show median and interquartile range and each point represents one participant. The dashed line indicates no change. (a) n=98 for HR group, n=73 for ACS, n=173 for stroke. (b) n=98 for HR group, n=74 for ACS, n=175 for stroke. (c) n=94 for HR group, n=67 for ACS, n=169 for stroke.

**Supplementary Table Legend**

**Supplementary Table 1a:** SEM results for CV events – Combined model for MR-proANP. Explained variance R^2^=0.11 for ∆MR-proANP and R^2^=0.43 for MR-proANP.

**Supplementary Table 1b:** SEM results for CV events – Combined model for NT-proBNP.

**Supplementary Table 1c:** SEM results for CV events – Combined model for Copeptin.

**Supplementary Table 1d:** SEM results for CV events (sensitivity analysis with adjustment for AF and GFR) – Combined model for MR-proANP.

**Supplementary Table 1e:** SEM results for CV events (sensitivity analysis with adjustment for AF and GFR) – Combined model for NT-proBNP.

**Supplementary Table 1f:** SEM results for CV events (sensitivity analysis with adjustment for AF and GFR) – Combined model for Copeptin.

**Supplementary Table 1g.** SEM results for CV events (sensitivity analysis with adjustment for SGLT2i) – Combined model for MR-proANP.

**Supplementary Table 1h.** SEM results for CV events (sensitivity analysis with adjustment for SGLT2i) – Combined model for NT-proBNP.

**Supplementary Table 1i.** SEM results for CV events (sensitivity analysis with adjustment for SGLT2i) – Combined model for Copeptin.

**Supplementary Table 1j.** SEM results for CV events (sensitivity analysis with adjustment for GLP-1 RA) – Combined model for MR-proANP.

**Supplementary Table 1k.** SEM results for CV events (sensitivity analysis with adjustment for GLP-1 RA) – Combined model for NT-proBNP.

**Supplementary Table 1l.** SEM results for CV events (sensitivity analysis with adjustment for GLP-1 RA) – Combined model for Copeptin.

**Supplementary Table 2a:** SEM results for CV events – Stroke Subanalysis for MR-proANP.

**Supplementary Table 2b:** SEM results for CV events – Stroke Subanalysis for NT-proBNP.

**Supplementary Table 2c:** SEM results for CV events – Stroke Subanalysis for Copeptin.

**Supplementary Table 3a:** SEM results for CV events – ACS Subanalysis for MR-proANP.

**Supplementary Table 3b:** SEM results for CV events – ACS Subanalysis for NT-proBNP.

**Supplementary Table 3c:** SEM results for CV events – ACS Subanalysis for Copeptin.

**Supplementary Table 4a:** SEM results for T2D – Combined model for MR-proANP.

**Supplementary Table 4b:** SEM results for T2D – Combined model for NT-proBNP.

**Supplementary Table 4c:** SEM results for T2D – Combined model for Copeptin.

**Supplementary Table 4d:** SEM results for T2D (sensitivity analysis with adjustment for AF and GFR) – Combined model for MR-proANP.

**Supplementary Table 4e:** SEM results for T2D (sensitivity analysis with adjustment for AF and GFR) – Combined model for NT-proBNP.

**Supplementary Table 4f:** SEM results for T2D (sensitivity analysis with adjustment for AF and GFR) – Combined model for Copeptin.

**Supplementary Table 5a:** SEM results for T2D – Stroke Subanalysis for MR-proANP.

**Supplementary Table 5b:** SEM results for T2D – Stroke Subanalysis for NT-proBNP.

**Supplementary Table 5c:** SEM results for T2D – Stroke Subanalysis for Copeptin.

**Supplementary Table 6a:** SEM results for T2D – ACS Subanalysis for MR-proANP.

**Supplementary Table 6b:** SEM results for T2D – ACS Subanalysis for NT-proBNP.

**Supplementary Table 6c:** SEM results for T2D – ACS Subanalysis for Copeptin.

**Supplementary Table 7a:** SEM results for HOMA-IR – Combined model for MR-proANP.

**Supplementary Table 7b:** SEM results for HOMA-IR – Combined model for NT-proBNP.

**Supplementary Table 7c:** SEM results for HOMA-IR – Combined model for Copeptin.

**Supplementary Table 7d:** SEM results for HOMA-IR (sensitivity analysis with adjustment for AF and GFR) – Combined model for MR-proANP.

**Supplementary Table 7e:** SEM results for HOMA-IR (sensitivity analysis with adjustment for AF and GFR) – Combined model for NT-proBNP.

**Supplementary Table 7f:** SEM results for HOMA-IR (sensitivity analysis with adjustment for AF and GFR) – Combined model for Copeptin.

**Supplementary Table 7g.** SEM results for HOMA-IR (after exclusion of participants receiving insulin therapy) – Combined model for MR-proANP

**Supplementary Table 7h.** SEM results for HOMA-IR (after exclusion of participants receiving insulin therapy) – Combined model for NT-proBNP

**Supplementary Table 7i.** SEM results for HOMA-IR (after exclusion of participants receiving insulin therapy) – Combined model for Copeptin

**Supplementary Table 7j:** SEM results for HOMA-IR (sensitivity analysis with adjustment for SGLT2i) – Combined model for MR-proANP.

**Supplementary Table 7k:** SEM results for HOMA-IR (sensitivity analysis with adjustment for SGLT2i) – Combined model for NT-proBNP.

**Supplementary Table 7l:** SEM results for HOMA-IR (sensitivity analysis with adjustment for SGLT2i) – Combined model for Copeptin.

**Supplementary Table 8a:** SEM results for HOMA-IR – Stroke Subanalysis for MR-proANP.

**Supplementary Table 8b:** SEM results for HOMA-IR – Stroke Subanalysis for NT-proBNP.

**Supplementary Table 8c:** SEM results for HOMA-IR – Stroke Subanalysis for Copeptin.

**Supplementary Table 9a:** SEM results for HOMA-IR – ACS Subanalysis for MR-proANP.

**Supplementary Table 9b:** SEM results for HOMA-IR – ACS Subanalysis for NT-proBNP.

**Supplementary Table 9c:** SEM results for HOMA-IR – ACS Subanalysis for Copeptin.

**Supplementary Table 10:** Index event-visit interval and MR-proANP, NT-proBNP and Copeptin

**Supplementary Table 11a.** SEM results for antidiabetic medication use within the HR group for MR-proANP

**Supplementary Table 11b.** SEM results for antidiabetic medication use within the HR group for NT-proBNP

**Supplementary Table 11c.** SEM results for antidiabetic medication use within the HR group for Copeptin

**Supplementary Table 12:** Clinical characteristics of the stroke cohort.

**Supplementary Table 13.** Contrast estimates comparing ACS and stroke.

**Supplementary Table 14.** Missing data per SEM.

**Supplementary Table 15.** Comparison of complete-case (CC) and full information maximum likelihood (FIML) estimates.

**Supplementary Table 1a**

**Supplementary Table 1a.** SEM results for CV events – Combined model for MR-proANP. Explained variance R^2^=0.11 for ∆MR-proANP and R^2^=0.43 for MR-proANP. MR-proANP refers to the natural log-transformed fasting peptide concentration. ∆MR-proANP refers to the change in log-transformed concentrations (ln(postprandial) - ln(fasting)).

| **Outcome** | **Predictor** | ***SE*** | ***Z*** | ***p*** | ***b*** | **95% CI (*b*)** | ***b**** | **95% CI (*b**)** |
| --- | --- | --- | --- | --- | --- | --- | --- | --- |
| ∆MR-proANP | MR-proANP | 0.02 | -3.11 | .002** | -0.06 | [-0.10, -0.02] | -0.31 | [-0.50, -0.12] |
| ∆MR-proANP | CV event | 0.02 | 1.04 | .299 | 0.02 | [-0.02, 0.07] | 0.09 | [-0.08, 0.25] |
| ∆MR-proANP | age | 0.01 | -0.03 | .977 | -0.00 | [-0.02, 0.02] | -0.00 | [-0.20, 0.19] |
| ∆MR-proANP | sex | 0.02 | 0.69 | .493 | 0.02 | [-0.03, 0.06] | 0.06 | [-0.10, 0.21] |
| ∆MR-proANP | BMI | 0.00 | -2.49 | .013* | -0.00 | [-0.01, -0.00] | -0.20 | [-0.36, -0.05] |
| MR-proANP | CV event | 0.09 | 3.31 | .001*** | 0.31 | [0.13, 0.49] | 0.22 | [0.09, 0.34] |
| MR-proANP | age | 0.04 | 8.20 | < .001*** | 0.34 | [0.26, 0.43] | 0.55 | [0.44, 0.66] |
| MR-proANP | sex | 0.10 | -1.93 | .054 | -0.18 | [-0.37, 0.00] | -0.12 | [-0.25, 0.00] |
| MR-proANP | BMI | 0.01 | 0.88 | .377 | 0.01 | [-0.01, 0.02] | 0.06 | [-0.07, 0.19] |

**Supplementary Table 1b**

**Supplementary Table 1b.** SEM results for CV events – Combined model for NT-proBNP. Explained variance R^2^=0.07 for ∆NT-proBNP and R^2^=0.36 for NT-proBNP. *NT-proBNP* refers to the natural log-transformed fasting peptide concentration. *∆NT-proBNP* refers to the change in log-transformed concentrations (ln(postprandial) - ln(fasting)).

| **Outcome** | **Predictor** | ***SE*** | ***Z*** | ***p*** | ***b*** | **95% CI (b)** | **b*** | **95% CI (b*)** |
| --- | --- | --- | --- | --- | --- | --- | --- | --- |
| ∆NT-proBNP | NT-proBNP | 0.01 | -1.63 | .102 | -0.02 | [-0.03, 0.00] | -0.16 | [-0.35, 0.03] |
| ∆NT-proBNP | CV event | 0.02 | 1.01 | .314 | 0.03 | [-0.02, 0.07] | 0.09 | [-0.08, 0.26] |
| ∆NT-proBNP | Age | 0.01 | 3.09 | .002** | 0.04 | [0.01, 0.06] | 0.29 | [0.12, 0.47] |
| ∆NT-proBNP | Sex | 0.02 | -0.93 | .355 | -0.02 | [-0.07, 0.03] | -0.08 | [-0.23, 0.08] |
| ∆NT-proBNP | BMI | 0.00 | 1.40 | .161 | 0.00 | [-0.00, 0.01] | 0.12 | [-0.04, 0.28] |
| NT-proBNP | CV event | 0.20 | 4.10 | < .001*** | 0.80 | [0.42, 1.19] | 0.28 | [0.15, 0.41] |
| NT-proBNP | Age | 0.09 | 6.23 | < .001*** | 0.55 | [0.38, 0.72] | 0.44 | [0.32, 0.56] |
| NT-proBNP | sex | 0.20 | -1.03 | .302 | -0.21 | [-0.60, 0.19] | -0.07 | [-0.20, 0.06] |
| NT-proBNP | BMI | 0.01 | 0.15 | .879 | 0.00 | [-0.03, 0.03] | 0.01 | [-0.12, 0.15] |

**Supplementary Table 1c**

**Supplementary Table 1c.** SEM results for CV events – Combined model for Copeptin. Explained variance R^2^=0.16 for ∆Copeptin and R^2^=0.15 for Copeptin. *Copeptin* refers to the natural log-transformed fasting peptide concentration. *∆Copeptin* refers to the change in log-transformed concentrations (ln(postprandial) - ln(fasting)).

| **Outcome** | **Predictor** | ***SE*** | ***Z*** | ***p*** | ***b*** | **95% CI (b)** | **b*** | **95% CI (b*)** |
| --- | --- | --- | --- | --- | --- | --- | --- | --- |
| ∆Copeptin | Copeptin | 0.04 | -3.26 | .001** | -0.13 | [-0.21, -0.05] | -0.27 | [-0.42, -0.11] |
| ∆Copeptin | CV event | 0.06 | -1.64 | .101 | -0.09 | [-0.20, 0.02] | -0.13 | [-0.29, 0.02] |
| ∆Copeptin | Age | 0.03 | 2.51 | .012* | 0.06 | [0.01, 0.11] | 0.21 | [0.05, 0.37] |
| ∆Copeptin | Sex | 0.06 | 1.22 | .222 | 0.07 | [-0.04, 0.19] | 0.10 | [-0.06, 0.27] |
| ∆Copeptin | BMI | 0.00 | 1.89 | .059 | 0.01 | [-0.00, 0.02] | 0.16 | [-0.00, 0.32] |
| Copeptin | CV event | 0.11 | 0.53 | .597 | 0.06 | [-0.16, 0.28] | 0.04 | [-0.12, 0.20] |
| Copeptin | age | 0.05 | 1.99 | .047* | 0.10 | [0.00, 0.20] | 0.16 | [0.00, 0.32] |
| Copeptin | sex | 0.12 | -4.40 | < .001*** | -0.52 | [-0.75, -0.29] | -0.35 | [-0.49, -0.21] |
| Copeptin | BMI | 0.01 | 2.44 | .015* | 0.02 | [0.00, 0.04] | 0.20 | [0.04, 0.35] |

**Supplementary Table 1d**

**Supplementary Table 1d.** SEM results for CV events (sensitivity analysis with adjustment for AF and GFR) – Combined model for MR-proANP. Explained variance R^2^=0.11 for ∆MR-proANP and R^2^=0.57 for MR-proANP. MR-proANP refers to the natural log-transformed fasting peptide concentration. ∆MR-proANP refers to the change in log-transformed concentrations (ln(postprandial) - ln(fasting)).

| **Outcome** | **Predictor** | ***SE*** | ***Z*** | ***p*** | ***b*** | **95% CI (b)** | ***b**** | **95% CI (b*)** |
| --- | --- | --- | --- | --- | --- | --- | --- | --- |
| ∆MR-proANP | MR-proANP | 0.02 | -2.69 | .007** | -0.06 | [-0.11, -0.02] | -0.31 | [-0.53, -0.09] |
| ∆MR-proANP | CV event | 0.02 | 1.06 | .291 | 0.03 | [-0.02, 0.07] | 0.09 | [-0.08, 0.26] |
| ∆MR-proANP | age | 0.01 | 0.18 | .855 | 0.00 | [-0.02, 0.03] | 0.02 | [-0.19, 0.23] |
| ∆MR-proANP | sex | 0.02 | 0.82 | .410 | 0.02 | [-0.03, 0.07] | 0.07 | [-0.09, 0.23] |
| ∆MR-proANP | BMI | 0.00 | -2.47 | .013* | -0.00 | [-0.01, -0.00] | -0.20 | [-0.36, -0.05] |
| ∆MR-proANP | GFR | 0.00 | 0.59 | .558 | 0.00 | [-0.00, 0.00] | 0.06 | [-0.14, 0.25] |
| ∆MR-proANP | AF | 0.03 | -0.52 | .605 | -0.01 | [-0.07, 0.04] | -0.04 | [-0.21, 0.12] |
| MR-proANP | CV event | 0.08 | 3.83 | < .001*** | 0.31 | [0.15, 0.47] | 0.22 | [0.11, 0.33] |
| MR-proANP | age | 0.04 | 4.54 | < .001*** | 0.20 | [0.11, 0.28] | 0.31 | [0.18, 0.44] |
| MR-proANP | sex | 0.08 | -1.74 | .081 | -0.15 | [-0.31, 0.02] | -0.10 | [-0.21, 0.01] |
| MR-proANP | BMI | 0.01 | 0.34 | .734 | 0.00 | [-0.01, 0.01] | 0.02 | [-0.09, 0.13] |
| MR-proANP | GFR | 0.00 | -4.59 | < .001*** | -0.01 | [-0.01, -0.01] | -0.30 | [-0.42, -0.17] |
| MR-proANP | AF | 0.09 | -5.03 | < .001*** | -0.47 | [-0.65, -0.29] | -0.28 | [-0.38, -0.17] |

**Supplementary Table 1e**

**Supplementary Table 1e.** SEM results for CV events (sensitivity analysis with adjustment for AF and GFR) – Combined model for NT-proBNP. Explained variance R^2^=0.08 for ∆NT-proBNP and R^2^=0.49 for NT-proBNP. *NT-proBNP* refers to the natural log-transformed fasting peptide concentration. *∆NT-proBNP* refers to the change in log-transformed concentrations (ln(postprandial) - ln(fasting)).

| **Outcome** | **Predictor** | ***SE*** | ***Z*** | ***p*** | ***b*** | **95% CI (b)** | ***b**** | **95% CI (b*)** |
| --- | --- | --- | --- | --- | --- | --- | --- | --- |
| ∆NT-proBNP | NT-proBNP | 0.01 | -0.95 | .344 | -0.01 | [-0.03, 0.01] | -0.10 | [-0.31, 0.11] |
| ∆NT-proBNP | CV event | 0.03 | 0.80 | .424 | 0.02 | [-0.03, 0.07] | 0.07 | [-0.10, 0.24] |
| ∆NT-proBNP | age | 0.01 | 3.01 | .003** | 0.04 | [0.01, 0.07] | 0.32 | [0.12, 0.52] |
| ∆NT-proBNP | sex | 0.02 | -1.03 | .305 | -0.03 | [-0.07, 0.02] | -0.08 | [-0.24, 0.08] |
| ∆NT-proBNP | BMI | 0.00 | 1.50 | .133 | 0.00 | [-0.00, 0.01] | 0.12 | [-0.04, 0.29] |
| ∆NT-proBNP | GFR | 0.00 | 0.46 | .649 | 0.00 | [-0.00, 0.00] | 0.04 | [-0.14, 0.23] |
| ∆NT-proBNP | AF | 0.03 | 1.09 | .277 | 0.03 | [-0.03, 0.09] | 0.10 | [-0.08, 0.27] |
| NT-proBNP | CV event | 0.18 | 4.65 | < .001*** | 0.82 | [0.47, 1.16] | 0.29 | [0.17, 0.41] |
| NT-proBNP | age | 0.09 | 3.68 | < .001*** | 0.35 | [0.16, 0.53] | 0.28 | [0.13, 0.42] |
| NT-proBNP | sex | 0.18 | -0.38 | .701 | -0.07 | [-0.43, 0.29] | -0.02 | [-0.14, 0.10] |
| NT-proBNP | BMI | 0.01 | -0.32 | .749 | -0.00 | [-0.03, 0.02] | -0.02 | [-0.14, 0.10] |
| NT-proBNP | GFR | 0.00 | -2.06 | .040* | -0.01 | [-0.02, -0.00] | -0.15 | [-0.28, -0.01] |
| NT-proBNP | AF | 0.20 | -5.49 | < .001*** | -1.11 | [-1.51, -0.72] | -0.33 | [-0.44, -0.22] |

**Supplementary Table 1f**

**Supplementary Table 1f.** SEM results for CV events (sensitivity analysis with adjustment for AF and GFR) – Combined model for Copeptin. Explained variance R^2^=0.16 for ∆Copeptin and R^2^=0.25 for Copeptin. *Copeptin* refers to the natural log-transformed fasting peptide concentration. *∆Copeptin* refers to the change in log-transformed concentrations (ln(postprandial) - ln(fasting)).

| **Outcome** | **Predictor** | **SE** | **Z** | **p** | **b** | **95% CI (b)** | **b*** | **95% CI (b*)** |
| --- | --- | --- | --- | --- | --- | --- | --- | --- |
| ∆Copeptin | Copeptin | 0.04 | -3.35 | .001*** | -0.14 | [-0.22, -0.06] | -0.29 | [-0.46, -0.13] |
| ∆Copeptin | CV event | 0.06 | -1.66 | .098 | -0.09 | [-0.20, 0.02] | -0.13 | [-0.29, 0.02] |
| ∆Copeptin | age | 0.03 | 1.69 | .092 | 0.05 | [-0.01, 0.11] | 0.17 | [-0.02, 0.36] |
| ∆Copeptin | sex | 0.06 | 1.00 | .320 | 0.06 | [-0.06, 0.18] | 0.09 | [-0.08, 0.25] |
| ∆Copeptin | BMI | 0.00 | 1.88 | .060 | 0.01 | [-0.00, 0.02] | 0.15 | [-0.00, 0.31] |
| ∆Copeptin | GFR | 0.00 | -0.90 | .368 | -0.00 | [-0.00, 0.00] | -0.09 | [-0.27, 0.10] |
| ∆Copeptin | AF | 0.06 | 0.04 | .972 | 0.00 | [-0.12, 0.13] | 0.00 | [-0.15, 0.16] |
| Copeptin | CV event | 0.11 | 0.51 | .608 | 0.06 | [-0.16, 0.27] | 0.04 | [-0.11, 0.19] |
| Copeptin | age | 0.06 | -0.67 | .502 | -0.04 | [-0.15, 0.07] | -0.06 | [-0.24, 0.12] |
| Copeptin | sex | 0.11 | -4.71 | < .001*** | -0.53 | [-0.75, -0.31] | -0.36 | [-0.49, -0.22] |
| Copeptin | BMI | 0.01 | 2.20 | .028* | 0.02 | [0.00, 0.03] | 0.17 | [0.02, 0.32] |
| Copeptin | GFR | 0.00 | -4.04 | < .001*** | -0.01 | [-0.02, -0.01] | -0.35 | [-0.51, -0.19] |
| Copeptin | AF | 0.12 | -1.65 | .100 | -0.20 | [-0.44, 0.04] | -0.12 | [-0.27, 0.02] |

**Supplementary Table 1g**

**Supplementary Table 1g.** SEM results for CV events (sensitivity analysis with adjustment for SGLT2i) – Combined model for MR-proANP. Explained variance R^2^=0.12 for ∆MR-proANP and R^2^=0.44 for MR-proANP. *MR-proANP* refers to the natural log-transformed fasting peptide concentration. *∆MR-proANP* refers to the change in log-transformed concentrations (ln(postprandial) - ln(fasting)).

| **Outcome** | **Predictor** | **SE** | **Z** | **p** | **b** | **95% CI (b)** | **b*** | **95% CI (b*)** |
| --- | --- | --- | --- | --- | --- | --- | --- | --- |
| ∆MR-proANP | MR-proANP | 0.02 | -2.9 | .004** | -0.06 | [-0.10, -0.02] | -0.29 | [-0.49, -0.10] |
| ∆MR-proANP | CV event | 0.02 | 1.18 | 0.238 | 0.03 | [-0.02, 0.08] | 0.1 | [-0.07, 0.27] |
| ∆MR-proANP | age | 0.01 | -0.06 | 0.951 | 0 | [-0.03, 0.02] | -0.01 | [-0.20, 0.19] |
| ∆MR-proANP | sex | 0.02 | 0.85 | 0.396 | 0.02 | [-0.03, 0.07] | 0.07 | [-0.09, 0.23] |
| ∆MR-proANP | BMI | 0 | -2.44 | .015* | 0 | [-0.01, -0.00] | -0.2 | [-0.35, -0.04] |
| ∆MR-proANP | SGLT2i | 0.03 | 1.04 | 0.299 | 0.03 | [-0.02, 0.08] | 0.08 | [-0.07, 0.24] |
| MR-proANP | CV event | 0.09 | 2.89 | .004** | 0.27 | [0.09, 0.45] | 0.19 | [0.06, 0.32] |
| MR-proANP | age | 0.04 | 8.13 | < .001*** | 0.34 | [0.26, 0.42] | 0.54 | [0.43, 0.65] |
| MR-proANP | sex | 0.09 | -2.24 | .025* | -0.21 | [-0.40, -0.03] | -0.14 | [-0.26, -0.02] |
| MR-proANP | BMI | 0.01 | 0.75 | 0.455 | 0.01 | [-0.01, 0.02] | 0.05 | [-0.08, 0.17] |
| MR-proANP | SGLT2i | 0.1 | -2.18 | .029* | -0.22 | [-0.42, -0.02] | -0.14 | [-0.26, -0.01] |
|  |  |  |  |  |  |  |  |  |
|  |  |  |  |  |  |  |  |  |

**Supplementary Table 1h**

**Supplementary Table 1h.** SEM results for CV events (sensitivity analysis with adjustment for SGLT2i) – Combined model for NT-proBNP. Explained variance R^2^=0.13 for ∆NT-proBNP and R^2^=0.38 for NT-proBNP. *NT-proBNP*  refers to the natural log-transformed fasting peptide concentration. *∆NT-proBNP* refers to the change in log-transformed concentrations (ln(postprandial) - ln(fasting)).

| **Outcome** | **Predictor** | **SE** | **Z** | **p** | **b** | **95% CI (b)** | **b*** | **95% CI (b*)** |
| --- | --- | --- | --- | --- | --- | --- | --- | --- |
| ∆NT-proBNP | NT-proBNP | 0.01 | -1.2 | 0.23 | -0.01 | [-0.03, 0.01] | -0.11 | [-0.30, 0.07] |
| ∆NT-proBNP | CV event | 0.02 | 1.49 | 0.137 | 0.04 | [-0.01, 0.08] | 0.13 | [-0.04, 0.29] |
| ∆NT-proBNP | age | 0.01 | 3.21 | .001** | 0.04 | [0.01, 0.06] | 0.3 | [0.12, 0.47] |
| ∆NT-proBNP | sex | 0.02 | -0.45 | 0.651 | -0.01 | [-0.06, 0.04] | -0.04 | [-0.19, 0.12] |
| ∆NT-proBNP | BMI | 0 | 1.66 | 0.096 | 0 | [-0.00, 0.01] | 0.13 | [-0.02, 0.29] |
| ∆NT-proBNP | SGLT2i | 0.03 | 3.27 | .001** | 0.08 | [0.03, 0.13] | 0.26 | [0.11, 0.40] |
| NT-proBNP | CV event | 0.2 | 3.73 | < .001*** | 0.73 | [0.35, 1.12] | 0.26 | [0.13, 0.39] |
| NT-proBNP | age | 0.09 | 6.14 | < .001*** | 0.54 | [0.37, 0.71] | 0.43 | [0.31, 0.55] |
| NT-proBNP | sex | 0.2 | -1.28 | 0.199 | -0.26 | [-0.65, 0.14] | -0.09 | [-0.22, 0.04] |
| NT-proBNP | BMI | 0.01 | 0.03 | 0.975 | 0 | [-0.03, 0.03] | 0 | [-0.13, 0.14] |
| NT-proBNP | SGLT2i | 0.21 | -1.82 | 0.069 | -0.39 | [-0.81, 0.03] | -0.12 | [-0.25, 0.01] |
|  |  |  |  |  |  |  |  |  |
|  |  |  |  |  |  |  |  |  |

**Supplementary Table 1i**

**Supplementary Table 1i.** SEM results for CV events (sensitivity analysis with adjustment for SGLT2i) – Combined model for Copeptin. Explained variance R^2^=0.16 for ∆Copeptin and R^2^=0.17 for Copeptin. *Copeptin* refers to the natural log-transformed fasting peptide concentration. *∆Copeptin* refers to the change in log-transformed concentrations (ln(postprandial) - ln(fasting)).

| **Outcome** | **Predictor** | **SE** | **Z** | **p** | **b** | **95% CI (b)** | **b*** | **95% CI (b*)** |
| --- | --- | --- | --- | --- | --- | --- | --- | --- |
| ∆Copeptin | Copeptin | 0.04 | -3.31 | .001*** | -0.13 | [-0.21, -0.05] | -0.27 | [-0.43, -0.12] |
| ∆Copeptin | CV event | 0.06 | -1.5 | 0.134 | -0.08 | [-0.20, 0.03] | -0.12 | [-0.28, 0.04] |
| ∆Copeptin | age | 0.03 | 2.55 | .011* | 0.06 | [0.01, 0.11] | 0.21 | [0.05, 0.37] |
| ∆Copeptin | sex | 0.06 | 1.26 | 0.207 | 0.08 | [-0.04, 0.19] | 0.11 | [-0.06, 0.27] |
| ∆Copeptin | BMI | 0 | 1.93 | 0.053 | 0.01 | [-0.00, 0.02] | 0.16 | [0.00, 0.32] |
| ∆Copeptin | SGLT2i | 0.06 | 0.54 | 0.591 | 0.03 | [-0.09, 0.15] | 0.04 | [-0.11, 0.20] |
| Copeptin | CV event | 0.12 | 0.9 | 0.369 | 0.1 | [-0.12, 0.33] | 0.07 | [-0.09, 0.23] |
| Copeptin | age | 0.05 | 2.12 | .034* | 0.11 | [0.01, 0.21] | 0.17 | [0.02, 0.33] |
| Copeptin | sex | 0.12 | -4.15 | < .001*** | -0.49 | [-0.71, -0.26] | -0.33 | [-0.47, -0.18] |
| Copeptin | BMI | 0.01 | 2.59 | .009** | 0.02 | [0.01, 0.04] | 0.21 | [0.06, 0.36] |
| Copeptin | SGLT2i | 0.12 | 1.92 | 0.055 | 0.24 | [-0.01, 0.48] | 0.15 | [-0.00, 0.30] |

**Supplementary Table 1j**

**Supplementary Table 1j.** SEM results for CV events (sensitivity analysis with adjustment for GLP1-1 RA) – Combined model for MR-proANP. Explained variance R^2^=0.14 for ∆MR-proANP and R^2^=0.43 for MR-proANP. *MR-proANP* refers to the natural log-transformed fasting peptide concentration. *∆MR-proANP* refers to the change in log-transformed concentrations (ln(postprandial) - ln(fasting)).

| **Outcome** | **Predictor** | **SE** | **Z** | **p** | **b** | **95% CI (b)** | **b*** | **95% CI (b*)** |
| --- | --- | --- | --- | --- | --- | --- | --- | --- |
| ∆MR-proANP | MR-proANP | 0.02 | -3.14 | .002** | -0.06 | [-0.10, -0.02] | -0.31 | [-0.50, -0.12] |
| ∆MR-proANP | CV event | 0.02 | 0.48 | 0.632 | 0.01 | [-0.04, 0.06] | 0.04 | [-0.13, 0.21] |
| ∆MR-proANP | age | 0.01 | -0.06 | 0.952 | 0 | [-0.02, 0.02] | -0.01 | [-0.20, 0.19] |
| ∆MR-proANP | sex | 0.02 | 0.76 | 0.450 | 0.02 | [-0.03, 0.06] | 0.06 | [-0.09, 0.21] |
| ∆MR-proANP | BMI | 0 | -2.17 | .030* | 0 | [-0.01, -0.00] | -0.18 | [-0.33, -0.02] |
| ∆MR-proANP | GLP-1 RA | 0.03 | -2.15 | .031* | -0.07 | [-0.13, -0.01] | -0.17 | [-0.32, -0.02] |
| MR-proANP | CV event | 0.1 | 3.23 | .001** | 0.31 | [0.12, 0.50] | 0.22 | [0.09, 0.35] |
| MR-proANP | age | 0.04 | 8.2 | < .001*** | 0.34 | [0.26, 0.43] | 0.55 | [0.44, 0.66] |
| MR-proANP | sex | 0.1 | -1.93 | 0.054 | -0.18 | [-0.37, 0.00] | -0.12 | [-0.25, 0.00] |
| MR-proANP | BMI | 0.01 | 0.85 | 0.393 | 0.01 | [-0.01, 0.02] | 0.06 | [-0.07, 0.19] |
| MR-proANP | GLP-1 RA | 0.13 | 0.12 | 0.904 | 0.02 | [-0.24, 0.27] | 0.01 | [-0.12, 0.13] |

**Supplementary Table 1k**

**Supplementary Table 1k.** SEM results for CV events (sensitivity analysis with adjustment for GLP-1 RA) – Combined model for NT-proBNP. Explained variance R^2^=0.11 for ∆NT-proBNP and R^2^=0.36 for NT-proBNP. *NT-proBNP*  refers to the natural log-transformed fasting peptide concentration. *∆NT-proBNP* refers to the change in log-transformed concentrations (ln(postprandial) - ln(fasting)).

| **Outcome** | **Predictor** | **SE** | **Z** | **p** | **b** | **95% CI (b)** | **b*** | **95% CI (b*)** |
| --- | --- | --- | --- | --- | --- | --- | --- | --- |
| ∆NT-proBNP | NT-proBNP | 0.01 | -1.6 | 0.110 | -0.02 | [-0.03, 0.00] | -0.15 | [-0.34, 0.03] |
| ∆NT-proBNP | CV event | 0.03 | 1.58 | 0.114 | 0.04 | [-0.01, 0.09] | 0.14 | [-0.03, 0.31] |
| ∆NT-proBNP | age | 0.01 | 3.15 | .002** | 0.04 | [0.01, 0.06] | 0.29 | [0.12, 0.47] |
| ∆NT-proBNP | sex | 0.02 | -1 | 0.316 | -0.02 | [-0.07, 0.02] | -0.08 | [-0.24, 0.08] |
| ∆NT-proBNP | BMI | 0 | 1.03 | 0.302 | 0 | [-0.00, 0.01] | 0.09 | [-0.08, 0.25] |
| ∆NT-proBNP | GLP-1 RA | 0.03 | 2.46 | .014* | 0.08 | [0.02, 0.14] | 0.2 | [0.04, 0.35] |
| NT-proBNP | CV event | 0.2 | 3.88 | < .001*** | 0.78 | [0.39, 1.18] | 0.28 | [0.14, 0.41] |
| NT-proBNP | age | 0.09 | 6.23 | < .001*** | 0.55 | [0.38, 0.72] | 0.44 | [0.32, 0.56] |
| NT-proBNP | sex | 0.2 | -1.02 | 0.306 | -0.21 | [-0.60, 0.19] | -0.07 | [-0.20, 0.06] |
| NT-proBNP | BMI | 0.01 | 0.2 | 0.840 | 0 | [-0.03, 0.03] | 0.01 | [-0.12, 0.15] |
| NT-proBNP | GLP-1 RA | 0.27 | -0.33 | 0.739 | -0.09 | [-0.62, 0.44] | -0.02 | [-0.16, 0.11] |

**Supplementary Table 1l**

**Supplementary Table 1l.** SEM results for CV events (sensitivity analysis with adjustment for GLP-1 RA) – Combined model for Copeptin. Explained variance R^2^=0.16 for ∆Copeptin and R^2^=0.15 for Copeptin. *Copeptin* refers to the natural log-transformed fasting peptide concentration. *∆Copeptin* refers to the change in log-transformed concentrations (ln(postprandial) - ln(fasting)).

| **Outcome** | **Predictor** | **SE** | **Z** | **p** | **b** | **95% CI (b)** | **b*** | **95% CI (b*)** |
| --- | --- | --- | --- | --- | --- | --- | --- | --- |
| ∆Copeptin | Copeptin | 0.04 | -3.26 | .001** | -0.13 | [-0.21, -0.05] | -0.26 | [-0.42, -0.11] |
| ∆Copeptin | CV event | 0.06 | -1.83 | 0.067 | -0.1 | [-0.22, 0.01] | -0.15 | [-0.32, 0.01] |
| ∆Copeptin | age | 0.03 | 2.51 | .012* | 0.06 | [0.01, 0.11] | 0.21 | [0.05, 0.37] |
| ∆Copeptin | sex | 0.06 | 1.25 | 0.212 | 0.07 | [-0.04, 0.19] | 0.1 | [-0.06, 0.27] |
| ∆Copeptin | BMI | 0 | 2.01 | .045* | 0.01 | [0.00, 0.02] | 0.17 | [0.01, 0.33] |
| ∆Copeptin | GLP-1 RA | 0.08 | -0.92 | 0.356 | -0.07 | [-0.22, 0.08] | -0.07 | [-0.23, 0.08] |
| Copeptin | CV event | 0.12 | 0.55 | 0.584 | 0.06 | [-0.17, 0.30] | 0.05 | [-0.12, 0.21] |
| Copeptin | age | 0.05 | 1.99 | .047* | 0.1 | [0.00, 0.20] | 0.17 | [0.01, 0.33] |
| Copeptin | sex | 0.12 | -4.4 | < .001*** | -0.52 | [-0.75, -0.29] | -0.35 | [-0.49, -0.21] |
| Copeptin | BMI | 0.01 | 2.39 | .017* | 0.02 | [0.00, 0.04] | 0.2 | [0.04, 0.35] |
| Copeptin | GLP-1 RA | 0.16 | 0.14 | 0.888 | 0.02 | [-0.29, 0.33] | 0.01 | [-0.15, 0.17] |

**Supplementary Table 2a**

**Supplementary Table 2a.** SEM results for CV events – Stroke Subanalysis for MR-proANP. Explained variance R^2^=0.12 for ∆MR-proANP and R^2^=0.40 for MR-proANP. *MR-proANP* refers to the natural log-transformed fasting peptide concentration. *∆MR-proANP* refers to the change in log-transformed concentrations (ln(postprandial) - ln(fasting)).

| **Outcome** | **Predictor** | ***SE*** | ***Z*** | ***p*** | ***b*** | **95% CI (b)** | **b*** | **95% CI (b*)** |
| --- | --- | --- | --- | --- | --- | --- | --- | --- |
| ∆MR-proANP | MR-proANP | 0.02 | -2.88 | .004** | -0.06 | [-0.10, -0.02] | -0.31 | [-0.51, -0.10] |
| ∆MR-proANP | CV event | 0.03 | 1.18 | .240 | 0.03 | [-0.02, 0.09] | 0.11 | [-0.07, 0.28] |
| ∆MR-proANP | age | 0.01 | -0.43 | .669 | -0.01 | [-0.03, 0.02] | -0.05 | [-0.26, 0.16] |
| ∆MR-proANP | sex | 0.02 | 0.40 | .690 | 0.01 | [-0.04, 0.06] | 0.03 | [-0.13, 0.20] |
| ∆MR-proANP | BMI | 0.00 | -2.54 | .011* | -0.00 | [-0.01, -0.00] | -0.22 | [-0.39, -0.06] |
| MR-proANP | CV event | 0.12 | 2.58 | .010** | 0.30 | [0.07, 0.53] | 0.19 | [0.05, 0.33] |
| MR-proANP | age | 0.05 | 7.29 | < .001*** | 0.35 | [0.26, 0.45] | 0.55 | [0.42, 0.67] |
| MR-proANP | sex | 0.10 | -1.51 | .131 | -0.16 | [-0.36, 0.05] | -0.11 | [-0.24, 0.03] |
| MR-proANP | BMI | 0.01 | 0.42 | .677 | 0.00 | [-0.01, 0.02] | 0.03 | [-0.11, 0.17] |

**Supplementary Table 2b**

**Supplementary Table 2b.** SEM results for CV events – Stroke Subanalysis for NT-proBNP. Explained variance R^2^=0.07 for ∆NT-proBNP and R^2^=0.34 for NT-proBNP. *NT-proBNP* refers to the natural log-transformed fasting peptide concentration. *∆NT-proBNP* refers to the change in log-transformed concentrations (ln(postprandial) - ln(fasting)).

| **Outcome** | **Predictor** | ***SE*** | ***Z*** | ***p*** | ***b*** | **95% CI (b)** | **b*** | **95% CI (b*)** |
| --- | --- | --- | --- | --- | --- | --- | --- | --- |
| ∆NT-proBNP | NT-proBNP | 0.01 | -1.22 | .221 | -0.01 | [-0.03, 0.01] | -0.13 | [-0.33, 0.08] |
| ∆NT-proBNP | CV event | 0.03 | 1.72 | .086 | 0.05 | [-0.01, 0.11] | 0.16 | [-0.02, 0.34] |
| ∆NT-proBNP | age | 0.01 | 2.05 | .040* | 0.03 | [0.00, 0.05] | 0.21 | [0.01, 0.41] |
| ∆NT-proBNP | sex | 0.03 | -0.81 | .416 | -0.02 | [-0.07, 0.03] | -0.07 | [-0.24, 0.10] |
| ∆NT-proBNP | BMI | 0.00 | 1.26 | .206 | 0.00 | [-0.00, 0.01] | 0.11 | [-0.06, 0.29] |
| NT-proBNP | CV event | 0.23 | 3.41 | .001*** | 0.80 | [0.34, 1.26] | 0.26 | [0.12, 0.40] |
| NT-proBNP | age | 0.10 | 5.51 | < .001*** | 0.53 | [0.34, 0.72] | 0.43 | [0.30, 0.57] |
| NT-proBNP | sex | 0.21 | -0.52 | .601 | -0.11 | [-0.51, 0.30] | -0.04 | [-0.18, 0.11] |
| NT-proBNP | BMI | 0.02 | -0.28 | .777 | -0.00 | [-0.04, 0.03] | -0.02 | [-0.17, 0.13] |

**Supplementary Table 2c**

**Supplementary Table 2c.** SEM results for CV events – Stroke Subanalysis for Copeptin. Explained variance R^2^=0.13 for ∆Copeptin and R^2^=0.18 for Copeptin. *Copeptin* refers to the natural log-transformed fasting peptide concentration. *∆Copeptin* refers to the change in log-transformed concentrations (ln(postprandial) - ln(fasting)).

| **Outcome** | **Predictor** | ***SE*** | ***Z*** | ***p*** | ***b*** | **95% CI (b)** | **b*** | **95% CI (b*)** |
| --- | --- | --- | --- | --- | --- | --- | --- | --- |
| ∆Copeptin | Copeptin | 0.05 | -2.43 | .015* | -0.11 | [-0.20, -0.02] | -0.22 | [-0.40, -0.05] |
| ∆Copeptin | CV event | 0.07 | -1.46 | .144 | -0.10 | [-0.24, 0.03] | -0.13 | [-0.30, 0.04] |
| ∆Copeptin | Age | 0.03 | 2.12 | .034* | 0.06 | [0.00, 0.12] | 0.20 | [0.02, 0.38] |
| ∆Copeptin | Sex | 0.07 | 1.08 | .282 | 0.07 | [-0.06, 0.20] | 0.10 | [-0.08, 0.28] |
| ∆Copeptin | BMI | 0.00 | 1.43 | .152 | 0.01 | [-0.00, 0.02] | 0.13 | [-0.05, 0.30] |
| Copeptin | CV event | 0.13 | 1.25 | .213 | 0.17 | [-0.10, 0.43] | 0.11 | [-0.06, 0.28] |
| Copeptin | age | 0.06 | 2.03 | .043* | 0.11 | [0.00, 0.22] | 0.18 | [0.01, 0.35] |
| Copeptin | sex | 0.12 | -4.36 | < .001*** | -0.52 | [-0.76, -0.29] | -0.37 | [-0.52, -0.22] |
| Copeptin | BMI | 0.01 | 1.92 | .055 | 0.02 | [-0.00, 0.04] | 0.17 | [-0.00, 0.33] |

**Supplementary Table 3a**

**Supplementary Table 3a.** SEM results for CV events – ACS Subanalysis for MR-proANP. Explained variance R^2^=0.16 for ∆MR-proANP and R^2^=0.41 for MR-proANP. *MR-proANP* refers to the natural log-transformed fasting peptide concentration. *∆MR-proANP* refers to the change in log-transformed concentrations (ln(postprandial) - ln(fasting)).

| **Outcome** | **Predictor** | ***SE*** | ***Z*** | ***p*** | ***b*** | **95% CI (b)** | **b*** | **95% CI (b*)** |
| --- | --- | --- | --- | --- | --- | --- | --- | --- |
| ∆MR-proANP | MR-proANP | 0.02 | -3.51 | < .001*** | -0.08 | [-0.12, -0.03] | -0.38 | [-0.58, -0.18] |
| ∆MR-proANP | CV event | 0.03 | 0.66 | .511 | 0.02 | [-0.04, 0.08] | 0.06 | [-0.12, 0.24] |
| ∆MR-proANP | age | 0.01 | 0.37 | .714 | 0.01 | [-0.02, 0.03] | 0.04 | [-0.17, 0.25] |
| ∆MR-proANP | sex | 0.03 | 0.76 | .447 | 0.02 | [-0.03, 0.07] | 0.07 | [-0.11, 0.24] |
| ∆MR-proANP | BMI | 0.00 | -2.73 | .006** | -0.01 | [-0.01, -0.00] | -0.24 | [-0.41, -0.08] |
| MR-proANP | CV event | 0.12 | 2.50 | .012* | 0.31 | [0.07, 0.55] | 0.18 | [0.04, 0.33] |
| MR-proANP | age | 0.05 | 7.82 | < .001*** | 0.36 | [0.27, 0.45] | 0.58 | [0.46, 0.69] |
| MR-proANP | sex | 0.11 | -1.13 | .260 | -0.12 | [-0.34, 0.09] | -0.08 | [-0.23, 0.06] |
| MR-proANP | BMI | 0.01 | 0.53 | .593 | 0.00 | [-0.01, 0.02] | 0.04 | [-0.11, 0.19] |

**Supplementary Table 3b**

**Supplementary Table 3b.** SEM results for CV events – ACS Subanalysis for NT-proBNP. Explained variance R^2^=0.05 for ∆NT-proBNP and R^2^=0.34 for NT-proBNP. *NT-proBNP* refers to the natural log-transformed fasting peptide concentration. *∆NT-proBNP* refers to the change in log-transformed concentrations (ln(postprandial) - ln(fasting)).

| **Outcome** | **Predictor** | ***SE*** | ***Z*** | ***p*** | ***b*** | **95% CI (b)** | **b*** | **95% CI (b*)** |
| --- | --- | --- | --- | --- | --- | --- | --- | --- |
| ∆NT-proBNP | NT-proBNP | 0.01 | -1.46 | .143 | -0.02 | [-0.04, 0.01] | -0.16 | [-0.37, 0.05] |
| ∆NT-proBNP | CV event | 0.03 | 0.02 | .987 | 0.00 | [-0.06, 0.07] | 0.00 | [-0.19, 0.19] |
| ∆NT-proBNP | Age | 0.01 | 2.50 | .012* | 0.03 | [0.01, 0.06] | 0.27 | [0.07, 0.47] |
| ∆NT-proBNP | Sex | 0.03 | -0.19 | .846 | -0.01 | [-0.06, 0.05] | -0.02 | [-0.20, 0.17] |
| ∆NT-proBNP | BMI | 0.00 | 0.62 | .536 | 0.00 | [-0.00, 0.01] | 0.06 | [-0.13, 0.24] |
| NT-proBNP | CV event | 0.26 | 3.26 | .001** | 0.85 | [0.34, 1.36] | 0.25 | [0.11, 0.40] |
| NT-proBNP | age | 0.10 | 6.20 | < .001*** | 0.59 | [0.41, 0.78] | 0.48 | [0.35, 0.61] |
| NT-proBNP | sex | 0.23 | -0.32 | .750 | -0.07 | [-0.52, 0.37] | -0.02 | [-0.18, 0.13] |
| NT-proBNP | BMI | 0.02 | 0.19 | .851 | 0.00 | [-0.03, 0.03] | 0.01 | [-0.14, 0.17] |

**Supplementary Table 3c**

**Supplementary Table 3c.** SEM results for CV events – ACS Subanalysis for Copeptin. Explained variance R^2^=0.17 for ∆Copeptin and R^2^=0.14 for Copeptin. *Copeptin* refers to the natural log-transformed fasting peptide concentration. *∆Copeptin* refers to the change in log-transformed concentrations (ln(postprandial) - ln(fasting)).

| **Outcome** | **Predictor** | ***SE*** | ***Z*** | ***p*** | ***b*** | **95% CI (b)** | ***b**** | **95% CI (b*)** |
| --- | --- | --- | --- | --- | --- | --- | --- | --- |
| ∆Copeptin | Copeptin | 0.05 | -3.05 | .002** | -0.14 | [-0.24, -0.05] | -0.27 | [-0.45, -0.10] |
| ∆Copeptin | CV event | 0.08 | -1.10 | .272 | -0.09 | [-0.24, 0.07] | -0.10 | [-0.27, 0.08] |
| ∆Copeptin | Age | 0.03 | 2.53 | .011* | 0.07 | [0.02, 0.13] | 0.23 | [0.06, 0.40] |
| ∆Copeptin | Sex | 0.07 | 1.32 | .186 | 0.10 | [-0.05, 0.24] | 0.13 | [-0.06, 0.31] |
| ∆Copeptin | BMI | 0.00 | 1.39 | .164 | 0.01 | [-0.00, 0.02] | 0.13 | [-0.05, 0.31] |
| Copeptin | CV event | 0.15 | -0.71 | .477 | -0.11 | [-0.41, 0.19] | -0.06 | [-0.24, 0.11] |
| Copeptin | Age | 0.06 | 1.36 | .173 | 0.08 | [-0.03, 0.18] | 0.12 | [-0.05, 0.30] |
| Copeptin | sex | 0.13 | -4.17 | < .001*** | -0.55 | [-0.81, -0.29] | -0.38 | [-0.54, -0.22] |
| Copeptin | BMI | 0.01 | 1.82 | .069 | 0.02 | [-0.00, 0.04] | 0.17 | [-0.01, 0.35] |

**Supplementary Table 4a**

**Supplementary Table 4a.** SEM results for T2D – Combined model for MR-proANP. Explained variance R^2^=0.05 for ∆MR-proANP and R^2^=0.37 for MR-proANP. *MR-proANP* refers to the natural log-transformed fasting peptide concentration. *∆MR-proANP* refers to the change in log-transformed concentrations (ln(postprandial) - ln(fasting)).

| **Outcome** | **Predictor** | **SE** | **Z** | **p** | **b** | **95% CI (b)** | **b*** | **95% CI (b*)** |
| --- | --- | --- | --- | --- | --- | --- | --- | --- |
| ∆MR-proANP | MR-proANP | 0.02 | -3.28 | .001** | -0.07 | [-0.12, -0.03] | -0.26 | [-0.41, -0.11] |
| ∆MR-proANP | diabetes | 0.03 | -0.00 | 1.00 | -0.00 | [-0.06, 0.06] | -0.00 | [-0.14, 0.14] |
| ∆MR-proANP | age | 0.01 | 1.34 | .181 | 0.02 | [-0.01, 0.04] | 0.11 | [-0.05, 0.27] |
| ∆MR-proANP | sex | 0.03 | 1.22 | .223 | 0.03 | [-0.02, 0.08] | 0.08 | [-0.05, 0.20] |
| ∆MR-proANP | BMI | 0.00 | -0.95 | .344 | -0.00 | [-0.01, 0.00] | -0.07 | [-0.20, 0.07] |
| MR-proANP | diabetes | 0.09 | -0.32 | .745 | -0.03 | [-0.20, 0.14] | -0.02 | [-0.13, 0.09] |
| MR-proANP | age | 0.03 | 11.64 | < .001*** | 0.32 | [0.26, 0.37] | 0.61 | [0.54, 0.69] |
| MR-proANP | sex | 0.07 | 0.69 | .490 | 0.05 | [-0.09, 0.19] | 0.04 | [-0.06, 0.14] |
| MR-proANP | BMI | 0.01 | 0.64 | .520 | 0.00 | [-0.01, 0.02] | 0.04 | [-0.07, 0.15] |

**Supplementary Table 4b**

**Supplementary Table 4b.** SEM results for T2D – Combined model for NT-proBNP. Explained variance R^2^=0.10 for ∆NT-proBNP and R^2^=0.26 for NT-proBNP. *NT-proBNP* refers to the natural log-transformed fasting peptide concentration. *∆NT-proBNP* refers to the change in log-transformed concentrations (ln(postprandial) - ln(fasting)).

| **Outcome** | **Predictor** | **SE** | **Z** | **p** | **b** | **95% CI (b)** | **b*** | **95% CI (b*)** |
| --- | --- | --- | --- | --- | --- | --- | --- | --- |
| ∆NT-proBNP | NT-proBNP | 0.01 | -2.58 | .010** | -0.02 | [-0.03, -0.00] | -0.18 | [-0.32, -0.05] |
| ∆NT-proBNP | diabetes | 0.02 | -0.73 | .464 | -0.02 | [-0.06, 0.03] | -0.05 | [-0.18, 0.08] |
| ∆NT-proBNP | age | 0.01 | 4.97 | < .001*** | 0.04 | [0.02, 0.05] | 0.36 | [0.22, 0.49] |
| ∆NT-proBNP | sex | 0.02 | -1.17 | .242 | -0.02 | [-0.06, 0.01] | -0.07 | [-0.19, 0.05] |
| ∆NT-proBNP | BMI | 0.00 | 0.49 | .627 | 0.00 | [-0.00, 0.00] | 0.03 | [-0.10, 0.16] |
| NT-proBNP | diabetes | 0.19 | 1.33 | .183 | 0.25 | [-0.12, 0.63] | 0.08 | [-0.04, 0.20] |
| NT-proBNP | age | 0.06 | 8.43 | < .001*** | 0.51 | [0.39, 0.63] | 0.48 | [0.39, 0.57] |
| NT-proBNP | sex | 0.16 | 1.86 | .063 | 0.30 | [-0.02, 0.61] | 0.10 | [-0.00, 0.21] |
| NT-proBNP | BMI | 0.02 | 0.67 | .503 | 0.01 | [-0.02, 0.04] | 0.04 | [-0.08, 0.16] |

**Supplementary Table 4c**

**Supplementary Table 4c.** SEM results for T2D – Combined model for Copeptin. Explained variance R^2^=0.13 for ∆Copeptin and R^2^=0.16 for Copeptin. *Copeptin* refers to the natural log-transformed fasting peptide concentration. *∆Copeptin* refers to the change in log-transformed concentrations (ln(postprandial) - ln(fasting)).

| **Outcome** | **Predictor** | **SE** | **Z** | **p** | **b** | **95% CI (b)** | **b*** | **95% CI (b*)** |
| --- | --- | --- | --- | --- | --- | --- | --- | --- |
| ∆Copeptin | Copeptin | 0.03 | -5.45 | < .001*** | -0.16 | [-0.22, -0.10] | -0.36 | [-0.48, -0.24] |
| ∆Copeptin | diabetes | 0.05 | 0.81 | .419 | 0.04 | [-0.06, 0.14] | 0.06 | [-0.08, 0.19] |
| ∆Copeptin | age | 0.02 | 0.71 | .476 | 0.01 | [-0.02, 0.04] | 0.05 | [-0.08, 0.17] |
| ∆Copeptin | sex | 0.04 | 0.43 | .671 | 0.02 | [-0.07, 0.10] | 0.03 | [-0.10, 0.15] |
| ∆Copeptin | BMI | 0.00 | 2.08 | .037* | 0.01 | [0.00, 0.02] | 0.14 | [0.01, 0.27] |
| Copeptin | diabetes | 0.11 | 1.04 | .296 | 0.12 | [-0.10, 0.33] | 0.07 | [-0.06, 0.20] |
| Copeptin | age | 0.04 | 2.65 | .008** | 0.09 | [0.02, 0.16] | 0.16 | [0.04, 0.28] |
| Copeptin | sex | 0.09 | -4.47 | < .001*** | -0.42 | [-0.60, -0.23] | -0.27 | [-0.38, -0.16] |
| Copeptin | BMI | 0.01 | 2.97 | .003** | 0.03 | [0.01, 0.05] | 0.20 | [0.07, 0.32] |

**Supplementary Table 4d**

**Supplementary Table 4d.** SEM results for T2D (sensitivity analysis with adjustment for AF and GFR) – Combined model for MR-proANP. Explained variance R^2^=0.04 for ∆MR-proANP and R^2^=0.53 for MR-proANP. *MR-proANP* refers to the natural log-transformed fasting peptide concentration. *∆MR-proANP* refers to the change in log-transformed concentrations (ln(postprandial) - ln(fasting)).

| **Outcome** | **Predictor** | **SE** | **Z** | **p** | **b** | **95% CI (b)** | **b*** | **95% CI (b*)** |
| --- | --- | --- | --- | --- | --- | --- | --- | --- |
| ∆MR-proANP | MR-proANP | 0.03 | -0.91 | .361 | -0.02 | [-0.07, 0.03] | -0.10 | [-0.31, 0.11] |
| ∆MR-proANP | diabetes | 0.03 | 0.02 | .983 | 0.00 | [-0.07, 0.07] | 0.00 | [-0.17, 0.17] |
| ∆MR-proANP | age | 0.01 | -1.34 | .179 | -0.02 | [-0.04, 0.01] | -0.14 | [-0.34, 0.06] |
| ∆MR-proANP | sex | 0.02 | 1.20 | .229 | 0.03 | [-0.02, 0.08] | 0.09 | [-0.06, 0.24] |
| ∆MR-proANP | BMI | 0.00 | -0.39 | .698 | -0.00 | [-0.01, 0.00] | -0.03 | [-0.20, 0.13] |
| ∆MR-proANP | GFR | 0.00 | -1.35 | .176 | -0.00 | [-0.00, 0.00] | -0.13 | [-0.32, 0.06] |
| ∆MR-proANP | AF | 0.03 | -0.44 | .661 | -0.01 | [-0.07, 0.05] | -0.04 | [-0.20, 0.13] |
| MR-proANP | diabetes | 0.10 | -0.08 | .939 | -0.01 | [-0.21, 0.19] | -0.00 | [-0.12, 0.11] |
| MR-proANP | age | 0.03 | 6.38 | < .001*** | 0.21 | [0.15, 0.28] | 0.42 | [0.30, 0.54] |
| MR-proANP | sex | 0.07 | 0.19 | .848 | 0.01 | [-0.13, 0.16] | 0.01 | [-0.09, 0.11] |
| MR-proANP | BMI | 0.01 | -0.57 | .569 | -0.00 | [-0.02, 0.01] | -0.03 | [-0.15, 0.08] |
| MR-proANP | GFR | 0.00 | -4.08 | < .001*** | -0.01 | [-0.01, -0.00] | -0.26 | [-0.39, -0.14] |
| MR-proANP | AF | 0.08 | -4.86 | < .001*** | -0.41 | [-0.57, -0.24] | -0.26 | [-0.37, -0.16] |

**Supplementary Table 4e**

**Supplementary Table 4e.** SEM results for T2D (sensitivity analysis with adjustment for AF and GFR) – Combined model for NT-proBNP. Explained variance R^2^=0.12 for ∆NT-proBNP and R^2^=0.55 for NT-proBNP. *NT-proBNP* refers to the natural log-transformed fasting peptide concentration. *∆NT-proBNP* refers to the change in log-transformed concentrations (ln(postprandial) - ln(fasting)).

| **Outcome** | **Predictor** | ***SE*** | ***Z*** | ***p*** | ***b*** | **95% CI (b)** | ***b**** | **95% CI (b*)** |
| --- | --- | --- | --- | --- | --- | --- | --- | --- |
| ∆NT-proBNP | NT-proBNP | 0.01 | -0.33 | .740 | -0.00 | [-0.02, 0.02] | -0.04 | [-0.24, 0.17] |
| ∆NT-proBNP | diabetes | 0.03 | 0.14 | .886 | 0.00 | [-0.05, 0.06] | 0.01 | [-0.15, 0.17] |
| ∆NT-proBNP | age | 0.01 | 3.88 | < .001*** | 0.04 | [0.02, 0.06] | 0.36 | [0.19, 0.54] |
| ∆NT-proBNP | sex | 0.02 | -1.44 | .149 | -0.03 | [-0.07, 0.01] | -0.11 | [-0.25, 0.04] |
| ∆NT-proBNP | BMI | 0.00 | 0.96 | .337 | 0.00 | [-0.00, 0.01] | 0.08 | [-0.08, 0.23] |
| ∆NT-proBNP | GFR | 0.00 | 1.85 | .064 | 0.00 | [-0.00, 0.00] | 0.17 | [-0.01, 0.35] |
| ∆NT-proBNP | AF | 0.03 | 1.90 | .058 | 0.05 | [-0.00, 0.10] | 0.17 | [-0.00, 0.34] |
| NT-proBNP | diabetes | 0.20 | 1.99 | .047* | 0.41 | [0.01, 0.81] | 0.12 | [0.00, 0.23] |
| NT-proBNP | age | 0.07 | 4.18 | < .001*** | 0.28 | [0.15, 0.42] | 0.27 | [0.14, 0.39] |
| NT-proBNP | sex | 0.15 | 2.43 | .015* | 0.36 | [0.07, 0.66] | 0.13 | [0.03, 0.23] |
| NT-proBNP | BMI | 0.02 | -0.97 | .330 | -0.02 | [-0.05, 0.02] | -0.06 | [-0.17, 0.06] |
| NT-proBNP | GFR | 0.00 | -3.41 | .001*** | -0.01 | [-0.02, -0.01] | -0.21 | [-0.34, -0.09] |
| NT-proBNP | AF | 0.17 | -8.23 | < .001*** | -1.40 | [-1.74, -1.07] | -0.44 | [-0.53, -0.34] |

**Supplementary Table 4f**

**Supplementary Table 4f.** SEM results for T2D (sensitivity analysis with adjustment for AF and GFR) – Combined model for Copeptin. Explained variance R^2^=0.21 for ∆Copeptin and R^2^=0.34 for Copeptin. *Copeptin* refers to the natural log-transformed fasting peptide concentration. *∆Copeptin* refers to the change in log-transformed concentrations (ln(postprandial) - ln(fasting)).

| **Outcome** | **Predictor** | **SE** | **Z** | **p** | **b** | **95% CI (b)** | **b*** | **95% CI (b*)** |
| --- | --- | --- | --- | --- | --- | --- | --- | --- |
| ∆Copeptin | Copeptin | 0.04 | -6.27 | < .001*** | -0.23 | [-0.30, -0.15] | -0.53 | [-0.68, -0.38] |
| ∆Copeptin | diabetes | 0.06 | 1.13 | .259 | 0.07 | [-0.05, 0.18] | 0.09 | [-0.07, 0.25] |
| ∆Copeptin | age | 0.02 | 0.65 | .515 | 0.01 | [-0.03, 0.05] | 0.06 | [-0.11, 0.23] |
| ∆Copeptin | sex | 0.05 | -1.10 | .273 | -0.05 | [-0.14, 0.04] | -0.08 | [-0.23, 0.07] |
| ∆Copeptin | BMI | 0.00 | 1.52 | .129 | 0.01 | [-0.00, 0.02] | 0.12 | [-0.03, 0.27] |
| ∆Copeptin | GFR | 0.00 | -0.98 | .329 | -0.00 | [-0.00, 0.00] | -0.09 | [-0.27, 0.09] |
| ∆Copeptin | AF | 0.05 | -0.51 | .609 | -0.02 | [-0.12, 0.07] | -0.04 | [-0.18, 0.10] |
| Copeptin | diabetes | 0.12 | 1.99 | .047* | 0.25 | [0.00, 0.49] | 0.14 | [0.00, 0.28] |
| Copeptin | age | 0.04 | -0.76 | .450 | -0.03 | [-0.11, 0.05] | -0.06 | [-0.22, 0.10] |
| Copeptin | sex | 0.09 | -5.67 | < .001*** | -0.52 | [-0.70, -0.34] | -0.36 | [-0.48, -0.25] |
| Copeptin | BMI | 0.01 | 1.66 | .096 | 0.02 | [-0.00, 0.04] | 0.12 | [-0.02, 0.25] |
| Copeptin | GFR | 0.00 | -5.50 | < .001*** | -0.01 | [-0.02, -0.01] | -0.43 | [-0.57, -0.29] |
| Copeptin | AF | 0.10 | -0.30 | .765 | -0.03 | [-0.24, 0.17] | -0.02 | [-0.15, 0.11] |

**Supplementary Table 5a**

**Supplementary Table 5a.** SEM results for T2D – Stroke Subanalysis for MR-proANP. Explained variance R^2^=0.04 for ∆MR-proANP and R^2^=0.39 for MR-proANP. *MR-proANP* refers to the natural log-transformed fasting peptide concentration. *∆MR-proANP* refers to the change in log-transformed concentrations (ln(postprandial) - ln(fasting)).

| **Outcome** | **Predictor** | **SE** | **Z** | **p** | **b** | **95% CI (b)** | **b*** | **95% CI (b*)** |
| --- | --- | --- | --- | --- | --- | --- | --- | --- |
| ∆MR-proANP | MR-proANP | 0.03 | -2.36 | .018* | -0.06 | [-0.12, -0.01] | -0.22 | [-0.41, -0.04] |
| ∆MR-proANP | diabetes | 0.04 | -0.00 | .999 | -0.00 | [-0.08, 0.08] | -0.00 | [-0.17, 0.17] |
| ∆MR-proANP | age | 0.01 | 0.84 | .399 | 0.01 | [-0.02, 0.04] | 0.08 | [-0.11, 0.27] |
| ∆MR-proANP | sex | 0.03 | 0.73 | .465 | 0.02 | [-0.04, 0.08] | 0.06 | [-0.09, 0.20] |
| ∆MR-proANP | BMI | 0.00 | -0.77 | .443 | -0.00 | [-0.01, 0.00] | -0.06 | [-0.23, 0.10] |
| MR-proANP | diabetes | 0.12 | 0.36 | .718 | 0.04 | [-0.19, 0.27] | 0.02 | [-0.11, 0.16] |
| MR-proANP | age | 0.03 | 9.87 | < .001*** | 0.31 | [0.25, 0.38] | 0.61 | [0.52, 0.70] |
| MR-proANP | sex | 0.08 | 0.86 | .392 | 0.07 | [-0.09, 0.24] | 0.05 | [-0.07, 0.17] |
| MR-proANP | BMI | 0.01 | -0.14 | .888 | -0.00 | [-0.02, 0.02] | -0.01 | [-0.14, 0.12] |

**Supplementary Table 5b**

**Supplementary Table 5b.** SEM results for T2D – Stroke Subanalysis for NT-proBNP. Explained variance R^2^=0.08 for ∆NT-proBNP and R^2^=0.33 for NT-proBNP. *NT-proBNP* refers to the natural log-transformed fasting peptide concentration. *∆NT-proBNP* refers to the change in log-transformed concentrations (ln(postprandial) - ln(fasting)).

| **Outcome** | **Predictor** | **SE** | **Z** | **p** | **b** | **95% CI (b)** | **b*** | **95% CI (b*)** |
| --- | --- | --- | --- | --- | --- | --- | --- | --- |
| ∆NT-proBNP | NT-proBNP | 0.01 | -2.05 | .041* | -0.02 | [-0.03, -0.00] | -0.18 | [-0.35, -0.01] |
| ∆NT-proBNP | diabetes | 0.03 | 0.22 | .829 | 0.01 | [-0.05, 0.06] | 0.02 | [-0.15, 0.18] |
| ∆NT-proBNP | age | 0.01 | 3.39 | .001*** | 0.03 | [0.01, 0.05] | 0.30 | [0.13, 0.46] |
| ∆NT-proBNP | sex | 0.02 | -1.35 | .178 | -0.03 | [-0.07, 0.01] | -0.10 | [-0.25, 0.04] |
| ∆NT-proBNP | BMI | 0.00 | 0.83 | .407 | 0.00 | [-0.00, 0.01] | 0.07 | [-0.09, 0.23] |
| NT-proBNP | diabetes | 0.25 | 1.90 | .057 | 0.47 | [-0.01, 0.96] | 0.13 | [-0.00, 0.27] |
| NT-proBNP | age | 0.07 | 7.84 | < .001*** | 0.53 | [0.40, 0.67] | 0.50 | [0.40, 0.61] |
| NT-proBNP | sex | 0.18 | 2.54 | .011* | 0.46 | [0.10, 0.81] | 0.16 | [0.04, 0.28] |
| NT-proBNP | BMI | 0.02 | -0.62 | .533 | -0.01 | [-0.05, 0.03] | -0.04 | [-0.18, 0.09] |

**Supplementary Table 5c**

**Supplementary Table 5c.** SEM results for T2D – Stroke Subanalysis for Copeptin. Explained variance R^2^=0.16 for ∆Copeptin and R^2^=0.22 for Copeptin. *Copeptin* refers to the natural log-transformed fasting peptide concentration. *∆Copeptin* refers to the change in log-transformed concentrations (ln(postprandial) - ln(fasting)).

| **Outcome** | **Predictor** | **SE** | **Z** | **p** | **b** | **95% CI (b)** | **b*** | **95% CI (b*)** |
| --- | --- | --- | --- | --- | --- | --- | --- | --- |
| ∆Copeptin | Copeptin | 0.04 | -5.43 | < .001*** | -0.20 | [-0.27, -0.13] | -0.43 | [-0.58, -0.29] |
| ∆Copeptin | diabetes | 0.07 | 1.12 | .261 | 0.07 | [-0.06, 0.20] | 0.09 | [-0.07, 0.25] |
| ∆Copeptin | age | 0.02 | 0.89 | .372 | 0.02 | [-0.02, 0.05] | 0.07 | [-0.08, 0.21] |
| ∆Copeptin | sex | 0.05 | -0.29 | .769 | -0.01 | [-0.11, 0.08] | -0.02 | [-0.17, 0.13] |
| ∆Copeptin | BMI | 0.01 | 1.68 | .094 | 0.01 | [-0.00, 0.02] | 0.13 | [-0.02, 0.29] |
| Copeptin | diabetes | 0.13 | 2.40 | .016* | 0.32 | [0.06, 0.59] | 0.19 | [0.04, 0.34] |
| Copeptin | age | 0.04 | 2.60 | .009** | 0.10 | [0.02, 0.17] | 0.18 | [0.05, 0.32] |
| Copeptin | sex | 0.10 | -4.52 | < .001*** | -0.45 | [-0.64, -0.25] | -0.31 | [-0.43, -0.18] |
| Copeptin | BMI | 0.01 | 1.69 | .091 | 0.02 | [-0.00, 0.04] | 0.13 | [-0.02, 0.28] |

**Supplementary Table 6a**

**Supplementary Table 6a.** SEM results for T2D – ACS Subanalysis for MR-proANP. Explained variance R^2^=0.09 for ∆MR-proANP and R^2^=0.37 for MR-proANP. *MR-proANP* refers to the natural log-transformed fasting peptide concentration. *∆MR-proANP* refers to the change in log-transformed concentrations (ln(postprandial) - ln(fasting)).

| **Outcome** | **Predictor** | **SE** | **Z** | **p** | **b** | **95% CI (b)** | **b*** | **95% CI (b*)** |
| --- | --- | --- | --- | --- | --- | --- | --- | --- |
| ∆MR-proANP | MR-proANP | 0.04 | -2.30 | .021* | -0.10 | [-0.19, -0.01] | -0.32 | [-0.59, -0.06] |
| ∆MR-proANP | diabetes | 0.05 | 0.03 | .978 | 0.00 | [-0.09, 0.09] | 0.00 | [-0.24, 0.25] |
| ∆MR-proANP | age | 0.02 | 1.18 | .237 | 0.03 | [-0.02, 0.08] | 0.18 | [-0.11, 0.47] |
| ∆MR-proANP | sex | 0.05 | 1.11 | .267 | 0.06 | [-0.04, 0.16] | 0.13 | [-0.10, 0.35] |
| ∆MR-proANP | BMI | 0.00 | -0.43 | .668 | -0.00 | [-0.01, 0.01] | -0.05 | [-0.30, 0.19] |
| MR-proANP | diabetes | 0.13 | -1.50 | .135 | -0.19 | [-0.43, 0.06] | -0.15 | [-0.35, 0.05] |
| MR-proANP | age | 0.05 | 6.44 | < .001*** | 0.33 | [0.23, 0.43] | 0.65 | [0.51, 0.79] |
| MR-proANP | sex | 0.14 | 0.12 | .908 | 0.02 | [-0.26, 0.29] | 0.01 | [-0.18, 0.20] |
| MR-proANP | BMI | 0.01 | 1.15 | .252 | 0.01 | [-0.01, 0.04] | 0.12 | [-0.08, 0.32] |

**Supplementary Table 6b**

**Supplementary Table 6b.** SEM results for T2D – ACS Subanalysis for NT-proBNP. Explained variance R^2^=0.19 for ∆NT-proBNP and R^2^=0.17 for NT-proBNP. *NT-proBNP* refers to the natural log-transformed fasting peptide concentration. *∆NT-proBNP* refers to the change in log-transformed concentrations (ln(postprandial) - ln(fasting)).

| **Outcome** | **Predictor** | **SE** | **Z** | **p** | **b** | **95% CI (b)** | **b*** | **95% CI (b*)** |
| --- | --- | --- | --- | --- | --- | --- | --- | --- |
| ∆NT-proBNP | NT-proBNP | 0.01 | -0.91 | .360 | -0.01 | [-0.04, 0.02] | -0.11 | [-0.33, 0.12] |
| ∆NT-proBNP | diabetes | 0.04 | -1.36 | .172 | -0.05 | [-0.12, 0.02] | -0.16 | [-0.38, 0.07] |
| ∆NT-proBNP | age | 0.02 | 3.63 | < .001*** | 0.06 | [0.03, 0.09] | 0.46 | [0.24, 0.68] |
| ∆NT-proBNP | sex | 0.04 | -0.01 | .991 | -0.00 | [-0.08, 0.08] | -0.00 | [-0.21, 0.21] |
| ∆NT-proBNP | BMI | 0.00 | -0.19 | .849 | -0.00 | [-0.01, 0.01] | -0.02 | [-0.25, 0.21] |
| NT-proBNP | diabetes | 0.28 | -0.88 | .379 | -0.25 | [-0.79, 0.30] | -0.10 | [-0.33, 0.12] |
| NT-proBNP | age | 0.11 | 3.90 | < .001*** | 0.45 | [0.22, 0.67] | 0.45 | [0.25, 0.65] |
| NT-proBNP | sex | 0.31 | -0.43 | .666 | -0.13 | [-0.75, 0.48] | -0.05 | [-0.26, 0.16] |
| NT-proBNP | BMI | 0.03 | 1.54 | .124 | 0.04 | [-0.01, 0.09] | 0.18 | [-0.04, 0.40] |

**Supplementary Table 6c**

**Supplementary Table 6c.** SEM results for T2D – ACS Subanalysis for Copeptin. Explained variance R^2^=0.09 for ∆Copeptin and R^2^=0.08 for Copeptin. *Copeptin* refers to the natural log-transformed fasting peptide concentration. *∆Copeptin* refers to the change in log-transformed concentrations (ln(postprandial) - ln(fasting)).

| **Outcome** | **Predictor** | **SE** | **Z** | **p** | **b** | **95% CI (b)** | **b*** | **95% CI (b*)** |
| --- | --- | --- | --- | --- | --- | --- | --- | --- |
| ∆Copeptin | Copeptin | 0.05 | -2.04 | .041* | -0.10 | [-0.19, -0.00] | -0.25 | [-0.48, -0.02] |
| ∆Copeptin | diabetes | 0.08 | 0.27 | .790 | 0.02 | [-0.13, 0.17] | 0.03 | [-0.22, 0.29] |
| ∆Copeptin | age | 0.03 | 0.16 | .873 | 0.01 | [-0.06, 0.07] | 0.02 | [-0.23, 0.27] |
| ∆Copeptin | sex | 0.09 | 1.13 | .259 | 0.10 | [-0.07, 0.27] | 0.14 | [-0.10, 0.37] |
| ∆Copeptin | BMI | 0.01 | 0.89 | .371 | 0.01 | [-0.01, 0.02] | 0.12 | [-0.14, 0.38] |
| Copeptin | diabetes | 0.20 | -1.00 | .317 | -0.20 | [-0.59, 0.19] | -0.13 | [-0.38, 0.12] |
| Copeptin | age | 0.08 | 0.84 | .400 | 0.07 | [-0.09, 0.24] | 0.11 | [-0.14, 0.35] |
| Copeptin | sex | 0.23 | -1.49 | .137 | -0.34 | [-0.78, 0.11] | -0.18 | [-0.41, 0.05] |
| Copeptin | BMI | 0.02 | 2.05 | .041* | 0.04 | [0.00, 0.08] | 0.26 | [0.02, 0.51] |

**Supplementary Table 7a**

**Supplementary Table 7a.** SEM results for HOMA-IR – Combined model for MR-proANP. Explained variance R^2^=0.07 for ∆MR-proANP and R^2^=0.39 for MR-proANP. *MR-proANP* refers to the natural log-transformed fasting peptide concentration. *∆MR-proANP* refers to the change in log-transformed concentrations (ln(postprandial) - ln(fasting)).

| **Outcome** | **Predictor** | **SE** | **Z** | **p** | **b** | **95% CI (b)** | **b*** | **95% CI (b*)** |
| --- | --- | --- | --- | --- | --- | --- | --- | --- |
| ∆MR-proANP | MR-proANP | 0.02 | -3.71 | < .001*** | -0.09 | [-0.13, -0.04] | -0.29 | [-0.44, -0.14] |
| ∆MR-proANP | HOMA-IR | 0.02 | -2.43 | .015* | -0.05 | [-0.09, -0.01] | -0.18 | [-0.33, -0.04] |
| ∆MR-proANP | age | 0.01 | 1.87 | .062 | 0.02 | [-0.00, 0.05] | 0.15 | [-0.01, 0.30] |
| ∆MR-proANP | sex | 0.03 | 1.05 | .294 | 0.03 | [-0.02, 0.08] | 0.07 | [-0.06, 0.19] |
| ∆MR-proANP | BMI | 0.00 | 0.48 | .632 | 0.00 | [-0.00, 0.01] | 0.04 | [-0.11, 0.18] |
| MR-proANP | HOMA-IR | 0.06 | -3.12 | .002** | -0.17 | [-0.28, -0.06] | -0.19 | [-0.30, -0.07] |
| MR-proANP | age | 0.03 | 12.50 | < .001*** | 0.32 | [0.27, 0.37] | 0.63 | [0.56, 0.70] |
| MR-proANP | sex | 0.07 | 0.57 | .569 | 0.04 | [-0.10, 0.18] | 0.03 | [-0.07, 0.13] |
| MR-proANP | BMI | 0.01 | 2.21 | .027* | 0.02 | [0.00, 0.03] | 0.13 | [0.02, 0.25] |

**Supplementary Table 7b**

**Supplementary Table 7b.** SEM results for HOMA-IR – Combined model for NT-proBNP. Explained variance R^2^=0.10 for ∆NT-proBNP and R^2^=0.27 for NT-proBNP. *NT-proBNP* refers to the natural log-transformed fasting peptide concentration. *∆NT-proBNP* refers to the change in log-transformed concentrations (ln(postprandial) - ln(fasting)).

| **Outcome** | **Predictor** | **SE** | **Z** | **p** | **b** | **95% CI (b)** | **b*** | **95% CI (b*)** |
| --- | --- | --- | --- | --- | --- | --- | --- | --- |
| ∆NT-proBNP | NT-proBNP | 0.01 | -2.83 | .005** | -0.02 | [-0.03, -0.01] | -0.20 | [-0.34, -0.06] |
| ∆NT-proBNP | HOMA-IR | 0.01 | -1.30 | .195 | -0.02 | [-0.05, 0.01] | -0.09 | [-0.24, 0.05] |
| ∆NT-proBNP | age | 0.01 | 5.07 | < .001*** | 0.04 | [0.02, 0.05] | 0.36 | [0.23, 0.49] |
| ∆NT-proBNP | sex | 0.02 | -1.15 | .250 | -0.02 | [-0.06, 0.01] | -0.07 | [-0.19, 0.05] |
| ∆NT-proBNP | BMI | 0.00 | 0.91 | .365 | 0.00 | [-0.00, 0.01] | 0.07 | [-0.08, 0.21] |
| NT-proBNP | HOMA-IR | 0.12 | -2.34 | .020* | -0.29 | [-0.53, -0.05] | -0.15 | [-0.28, -0.03] |
| NT-proBNP | age | 0.06 | 9.35 | < .001*** | 0.54 | [0.43, 0.65] | 0.51 | [0.43, 0.60] |
| NT-proBNP | sex | 0.16 | 1.57 | .116 | 0.25 | [-0.06, 0.56] | 0.09 | [-0.02, 0.19] |
| NT-proBNP | BMI | 0.02 | 2.42 | .016* | 0.04 | [0.01, 0.08] | 0.16 | [0.03, 0.28] |

**Supplementary Table 7c**

**Supplementary Table 7c.** SEM results for HOMA-IR – Combined model for Copeptin. R^2^=0.13 for ∆Copeptin and R^2^=0.16 for Copeptin. *Copeptin* refers to the natural log-transformed fasting peptide concentration. *∆Copeptin* refers to the change in log-transformed concentrations (ln(postprandial) - ln(fasting)).

| **Outcome** | **Predictor** | **SE** | **Z** | **p** | **b** | **95% CI (b)** | **b*** | **95% CI (b*)** |
| --- | --- | --- | --- | --- | --- | --- | --- | --- |
| ∆Copeptin | Copeptin | 0.03 | -5.42 | < .001*** | -0.16 | [-0.22, -0.10] | -0.36 | [-0.48, -0.24] |
| ∆Copeptin | HOMA-IR | 0.03 | 0.14 | .887 | 0.00 | [-0.06, 0.07] | 0.01 | [-0.13, 0.15] |
| ∆Copeptin | age | 0.02 | 0.93 | .353 | 0.01 | [-0.02, 0.05] | 0.06 | [-0.06, 0.18] |
| ∆Copeptin | sex | 0.04 | 0.30 | .768 | 0.01 | [-0.07, 0.10] | 0.02 | [-0.11, 0.14] |
| ∆Copeptin | BMI | 0.00 | 2.14 | .032* | 0.01 | [0.00, 0.02] | 0.16 | [0.02, 0.30] |
| Copeptin | HOMA-IR | 0.07 | 1.48 | .139 | 0.11 | [-0.04, 0.25] | 0.11 | [-0.03, 0.24] |
| Copeptin | age | 0.03 | 2.77 | .006** | 0.10 | [0.03, 0.16] | 0.17 | [0.05, 0.28] |
| Copeptin | sex | 0.09 | -4.58 | < .001*** | -0.42 | [-0.61, -0.24] | -0.27 | [-0.38, -0.16] |
| Copeptin | BMI | 0.01 | 2.33 | .020* | 0.02 | [0.00, 0.04] | 0.17 | [0.03, 0.30] |

**Supplementary Table 7d**

**Supplementary Table 7d.** SEM results for HOMA-IR (sensitivity analysis with adjustment for AF and GFR) – Combined model for MR-proANP. Explained variance R^2^=0.08 for ∆MR-proANP and R^2^=0.54 for MR-proANP. *MR-proANP* refers to the natural log-transformed fasting peptide concentration. *∆MR-proANP* refers to the change in log-transformed concentrations (ln(postprandial) - ln(fasting)).

| **Outcome** | **Predictor** | **SE** | **Z** | **p** | **b** | **95% CI (b)** | **b*** | **95% CI (b*)** |
| --- | --- | --- | --- | --- | --- | --- | --- | --- |
| ∆MR-proANP | MR-proANP | 0.02 | -3.09 | .002** | -0.07 | [-0.12, -0.03] | -0.28 | [-0.45, -0.11] |
| ∆MR-proANP | HOMA-IR | 0.02 | -2.90 | .004** | -0.05 | [-0.09, -0.02] | -0.22 | [-0.36, -0.07] |
| ∆MR-proANP | age | 0.01 | -0.12 | .901 | -0.00 | [-0.02, 0.02] | -0.01 | [-0.18, 0.16] |
| ∆MR-proANP | sex | 0.02 | 1.15 | .250 | 0.03 | [-0.02, 0.07] | 0.07 | [-0.05, 0.19] |
| ∆MR-proANP | BMI | 0.00 | 0.87 | .383 | 0.00 | [-0.00, 0.01] | 0.06 | [-0.08, 0.21] |
| ∆MR-proANP | GFR | 0.00 | -2.06 | .039* | -0.00 | [-0.00, -0.00] | -0.17 | [-0.33, -0.01] |
| ∆MR-proANP | AF | 0.03 | -1.37 | .172 | -0.04 | [-0.09, 0.02] | -0.09 | [-0.22, 0.04] |
| MR-proANP | HOMA-IR | 0.05 | -3.61 | < .001*** | -0.17 | [-0.27, -0.08] | -0.19 | [-0.29, -0.09] |
| MR-proANP | age | 0.03 | 7.59 | < .001*** | 0.21 | [0.16, 0.26] | 0.41 | [0.31, 0.51] |
| MR-proANP | sex | 0.06 | -0.57 | .568 | -0.04 | [-0.16, 0.09] | -0.03 | [-0.11, 0.06] |
| MR-proANP | BMI | 0.01 | 1.75 | .081 | 0.01 | [-0.00, 0.03] | 0.09 | [-0.01, 0.19] |
| MR-proANP | GFR | 0.00 | -5.56 | < .001*** | -0.01 | [-0.01, -0.01] | -0.30 | [-0.41, -0.20] |
| MR-proANP | AF | 0.07 | -5.07 | < .001*** | -0.35 | [-0.48, -0.21] | -0.23 | [-0.32, -0.14] |

**Supplementary Table 7e**

**Supplementary Table 7e.** SEM results for HOMA-IR (sensitivity analysis with adjustment for AF and GFR) – Combined model for NT-proBNP. Explained variance R^2^=0.11 for ∆NT-proBNP and R^2^=0.45 for NT-proBNP. *NT-proBNP* refers to the natural log-transformed fasting peptide concentration. *∆NT-proBNP* refers to the change in log-transformed concentrations (ln(postprandial) - ln(fasting)).

| **Outcome** | **Predictor** | ***SE*** | ***Z*** | ***p*** | ***b*** | **95% CI (b)** | ***b**** | **95% CI (b*)** |
| --- | --- | --- | --- | --- | --- | --- | --- | --- |
| ∆NT-proBNP | NT-proBNP | 0.01 | -1.64 | .101 | -0.01 | [-0.03, 0.00] | -0.13 | [-0.29, 0.03] |
| ∆NT-proBNP | HOMA-IR | 0.01 | -1.16 | .244 | -0.02 | [-0.04, 0.01] | -0.09 | [-0.23, 0.06] |
| ∆NT-proBNP | age | 0.01 | 4.92 | < .001*** | 0.04 | [0.03, 0.06] | 0.39 | [0.24, 0.53] |
| ∆NT-proBNP | sex | 0.02 | -1.09 | .276 | -0.02 | [-0.06, 0.02] | -0.07 | [-0.19, 0.05] |
| ∆NT-proBNP | BMI | 0.00 | 0.84 | .401 | 0.00 | [-0.00, 0.01] | 0.06 | [-0.08, 0.20] |
| ∆NT-proBNP | GFR | 0.00 | 0.71 | .480 | 0.00 | [-0.00, 0.00] | 0.05 | [-0.10, 0.21] |
| ∆NT-proBNP | AF | 0.02 | 1.60 | .109 | 0.04 | [-0.01, 0.08] | 0.11 | [-0.02, 0.25] |
| NT-proBNP | HOMA-IR | 0.11 | -2.56 | .010* | -0.28 | [-0.49, -0.07] | -0.15 | [-0.26, -0.04] |
| NT-proBNP | age | 0.06 | 4.98 | < .001*** | 0.31 | [0.19, 0.43] | 0.29 | [0.18, 0.41] |
| NT-proBNP | sex | 0.14 | 0.89 | .375 | 0.12 | [-0.15, 0.40] | 0.04 | [-0.05, 0.14] |
| NT-proBNP | BMI | 0.02 | 2.14 | .032* | 0.03 | [0.00, 0.06] | 0.12 | [0.01, 0.23] |
| NT-proBNP | GFR | 0.00 | -3.59 | < .001*** | -0.01 | [-0.02, -0.01] | -0.21 | [-0.33, -0.10] |
| NT-proBNP | AF | 0.16 | -7.65 | < .001*** | -1.19 | [-1.49, -0.88] | -0.38 | [-0.46, -0.29] |

**Supplementary Table 7f**

**Supplementary Table 7f.** SEM results for HOMA-IR (sensitivity analysis with adjustment for AF and GFR) – Combined model for Copeptin. Explained variance R^2^=0.15 for ∆Copeptin and R^2^=0.31 for Copeptin. *Copeptin* refers to the natural log-transformed fasting peptide concentration. *∆Copeptin* refers to the change in log-transformed concentrations (ln(postprandial) - ln(fasting)).

| **Outcome** | **Predictor** | ***SE*** | ***Z*** | ***p*** | ***b*** | **95% CI (b)** | ***b**** | **95% CI (b*)** |
| --- | --- | --- | --- | --- | --- | --- | --- | --- |
| ∆Copeptin | Copeptin | 0.03 | -5.80 | < .001*** | -0.17 | [-0.23, -0.11] | -0.42 | [-0.55, -0.29] |
| ∆Copeptin | HOMA-IR | 0.03 | 0.33 | .744 | 0.01 | [-0.05, 0.07] | 0.02 | [-0.12, 0.17] |
| ∆Copeptin | age | 0.02 | 0.78 | .438 | 0.01 | [-0.02, 0.05] | 0.06 | [-0.09, 0.21] |
| ∆Copeptin | sex | 0.04 | -0.21 | .834 | -0.01 | [-0.09, 0.07] | -0.01 | [-0.14, 0.12] |
| ∆Copeptin | BMI | 0.00 | 1.91 | .055 | 0.01 | [-0.00, 0.02] | 0.14 | [-0.00, 0.28] |
| ∆Copeptin | GFR | 0.00 | -0.58 | .559 | -0.00 | [-0.00, 0.00] | -0.05 | [-0.21, 0.11] |
| ∆Copeptin | AF | 0.04 | -0.08 | .940 | -0.00 | [-0.09, 0.08] | -0.00 | [-0.13, 0.12] |
| Copeptin | HOMA-IR | 0.07 | 1.39 | .166 | 0.09 | [-0.04, 0.23] | 0.09 | [-0.04, 0.22] |
| Copeptin | age | 0.04 | -1.53 | .126 | -0.06 | [-0.14, 0.02] | -0.10 | [-0.24, 0.03] |
| Copeptin | sex | 0.09 | -6.20 | < .001*** | -0.53 | [-0.70, -0.36] | -0.34 | [-0.44, -0.24] |
| Copeptin | BMI | 0.01 | 2.22 | .027* | 0.02 | [0.00, 0.04] | 0.14 | [0.02, 0.27] |
| Copeptin | GFR | 0.00 | -6.79 | < .001*** | -0.02 | [-0.02, -0.01] | -0.46 | [-0.59, -0.34] |
| Copeptin | AF | 0.09 | -0.51 | .611 | -0.05 | [-0.23, 0.14] | -0.03 | [-0.14, 0.08] |

**Supplementary Table 7g**

**Supplementary Table 7g.** SEM results for HOMA-IR (after exclusion of participants receiving insulin therapy) – Combined model for MR-proANP. Explained variance R^2^=0.08 for ∆MR-proANP and R^2^=0.4 for MR-proANP. *MR-proANP* refers to the natural log-transformed fasting peptide concentration. *∆MR-proANP* refers to the change in log-transformed concentrations (ln(postprandial) - ln(fasting)).

| **Outcome** | **Predictor** | **SE** | **Z** | **p** | **b** | **95% CI (b)** | **b*** | **95% CI (b*)** |
| --- | --- | --- | --- | --- | --- | --- | --- | --- |
| ∆MR-proANP | MR-proANP | 0.02 | -3.72 | < .001*** | -0.09 | [-0.13, -0.04] | -0.30 | [-0.46, -0.15] |
| ∆MR-proANP | HOMA-IR | 0.02 | -2.25 | .025* | -0.05 | [-0.09, -0.01] | -0.17 | [-0.31, -0.02] |
| ∆MR-proANP | age | 0.01 | 1.87 | .062 | 0.02 | [-0.00, 0.05] | 0.15 | [-0.01, 0.31] |
| ∆MR-proANP | sex | 0.03 | 1.05 | .295 | 0.03 | [-0.02, 0.08] | 0.07 | [-0.06, 0.19] |
| ∆MR-proANP | BMI | 0.00 | 0.06 | .951 | 0.00 | [-0.01, 0.01] | 0.00 | [-0.14, 0.15] |
| MR-proANP | HOMA-IR | 0.06 | -2.71 | .007** | -0.16 | [-0.28, -0.04] | -0.16 | [-0.28, -0.05] |
| MR-proANP | age | 0.03 | 12.29 | < .001*** | 0.32 | [0.27, 0.38] | 0.63 | [0.56, 0.70] |
| MR-proANP | sex | 0.07 | 0.79 | .428 | 0.06 | [-0.09, 0.20] | 0.04 | [-0.06, 0.14] |
| MR-proANP | BMI | 0.01 | 1.70 | .090 | 0.01 | [-0.00, 0.03] | 0.10 | [-0.02, 0.22] |
|  |  |  |  |  |  |  |  |  |
|  |  |  |  |  |  |  |  |  |
|  |  |  |  |  |  |  |  |  |
|  |  |  |  |  |  |  |  |  |

**Supplementary Table 7h**

**Supplementary Table 7h.** SEM results for HOMA-IR (after exclusion of participants receiving insulin therapy) – Combined model for NT-proBNP. Explained variance R^2^=0.11 for ∆NT-proBNP and R^2^=0.28 for NT-proBNP. *NT-proBNP* refers to the natural log-transformed fasting peptide concentration. *∆NT-proBNP* refers to the change in log-transformed concentrations (ln(postprandial) - ln(fasting)).

| **Outcome** | **Predictor** | ***SE*** | ***Z*** | ***p*** | ***b*** | **95% CI (b)** | ***b**** | **95% CI (b*)** |
| --- | --- | --- | --- | --- | --- | --- | --- | --- |
| ∆NT-proBNP | NT-proBNP | 0.01 | -2.76 | .006** | -0.02 | [-0.03, -0.01] | -0.20 | [-0.34, -0.06] |
| ∆NT-proBNP | HOMA-IR | 0.01 | -1.38 | .169 | -0.02 | [-0.05, 0.01] | -0.10 | [-0.24, 0.04] |
| ∆NT-proBNP | age | 0.01 | 5.05 | < .001*** | 0.04 | [0.02, 0.05] | 0.37 | [0.24, 0.50] |
| ∆NT-proBNP | sex | 0.02 | -1.05 | .294 | -0.02 | [-0.06, 0.02] | -0.07 | [-0.19, 0.06] |
| ∆NT-proBNP | BMI | 0.00 | 0.51 | .612 | 0.00 | [-0.00, 0.01] | 0.04 | [-0.11, 0.18] |
| NT-proBNP | HOMA-IR | 0.13 | -1.79 | .073 | -0.24 | [-0.49, 0.02] | -0.12 | [-0.24, 0.01] |
| NT-proBNP | age | 0.06 | 9.35 | < .001*** | 0.55 | [0.43, 0.66] | 0.52 | [0.44, 0.61] |
| NT-proBNP | sex | 0.16 | 1.78 | .076 | 0.29 | [-0.03, 0.61] | 0.10 | [-0.01, 0.21] |
| NT-proBNP | BMI | 0.02 | 1.79 | .074 | 0.03 | [-0.00, 0.07] | 0.12 | [-0.01, 0.24] |

**Supplementary Table 7i**

**Supplementary Table 7i.** SEM results for HOMA-IR (after exclusion of participants receiving insulin therapy) – Combined model for Copeptin. Explained variance R^2^=0.15 for ∆Copeptin and R^2^=0.14 for Copeptin. *Copeptin* refers to the natural log-transformed fasting peptide concentration. *∆Copeptin* refers to the change in log-transformed concentrations (ln(postprandial) - ln(fasting)).

| **Outcome** | **Predictor** | ***SE*** | ***Z*** | ***p*** | ***b*** | **95% CI (b)** | ***b**** | **95% CI (b*)** |
| --- | --- | --- | --- | --- | --- | --- | --- | --- |
| ∆Copeptin | Copeptin | 0.03 | -5.68 | < .001*** | -0.18 | [-0.24, -0.11] | -0.38 | [-0.50, -0.26] |
| ∆Copeptin | HOMA-IR | 0.04 | 0.01 | .996 | 0.00 | [-0.07, 0.07] | 0.00 | [-0.14, 0.14] |
| ∆Copeptin | age | 0.02 | 0.86 | .387 | 0.01 | [-0.02, 0.04] | 0.06 | [-0.07, 0.18] |
| ∆Copeptin | sex | 0.05 | -0.01 | .990 | -0.00 | [-0.09, 0.09] | -0.00 | [-0.13, 0.13] |
| ∆Copeptin | BMI | 0.01 | 2.14 | .032* | 0.01 | [0.00, 0.02] | 0.16 | [0.02, 0.30] |
| Copeptin | HOMA-IR | 0.08 | 0.44 | .663 | 0.03 | [-0.12, 0.18] | 0.03 | [-0.11, 0.17] |
| Copeptin | age | 0.03 | 2.62 | .009** | 0.09 | [0.02, 0.16] | 0.16 | [0.04, 0.28] |
| Copeptin | sex | 0.09 | -4.85 | < .001*** | -0.45 | [-0.63, -0.27] | -0.30 | [-0.41, -0.19] |
| Copeptin | BMI | 0.01 | 2.30 | .021* | 0.02 | [0.00, 0.05] | 0.17 | [0.03, 0.31] |
|  |  |  |  |  |  |  |  |  |
|  |  |  |  |  |  |  |  |  |
|  |  |  |  |  |  |  |  |  |
|  |  |  |  |  |  |  |  |  |

**Supplementary Table 7j**

**Supplementary Table 7j.** SEM results for HOMA-IR (sensitivity analysis with adjustment for SGLT2i) – Combined model for MR-proANP. Explained variance R^2^=0.08 for ∆MR-proANP and R^2^=0.4 for MR-proANP. *MR-proANP* refers to the natural log-transformed fasting peptide concentration. *∆MR-proANP* refers to the change in log-transformed concentrations (ln(postprandial) - ln(fasting)).

| **Outcome** | **Predictor** | **SE** | **Z** | **p** | **b** | **95% CI (b)** | **b*** | **95% CI (b*)** |
| --- | --- | --- | --- | --- | --- | --- | --- | --- |
| ∆MR-proANP | MR-proANP | 0.02 | -3.71 | < .001*** | -0.09 | [-0.13, -0.04] | -0.29 | [-0.44, -0.14] |
| ∆MR-proANP | HOMA-IR | 0.02 | -2.43 | .015* | -0.05 | [-0.09, -0.01] | -0.18 | [-0.33, -0.04] |
| ∆MR-proANP | age | 0.01 | 1.87 | 0.062 | 0.02 | [-0.00, 0.05] | 0.15 | [-0.01, 0.31] |
| ∆MR-proANP | sex | 0.03 | 1.05 | 0.296 | 0.03 | [-0.02, 0.08] | 0.06 | [-0.06, 0.19] |
| ∆MR-proANP | BMI | 0 | 0.48 | 0.630 | 0 | [-0.00, 0.01] | 0.04 | [-0.11, 0.18] |
| ∆MR-proANP | SGLT2i | 0.07 | -0.07 | 0.944 | 0 | [-0.14, 0.13] | 0 | [-0.13, 0.12] |
| MR-proANP | HOMA-IR | 0.06 | -3.07 | .002** | -0.17 | [-0.28, -0.06] | -0.18 | [-0.30, -0.07] |
| MR-proANP | age | 0.03 | 12.52 | < .001*** | 0.32 | [0.27, 0.37] | 0.63 | [0.56, 0.70] |
| MR-proANP | sex | 0.07 | 0.54 | 0.589 | 0.04 | [-0.10, 0.18] | 0.03 | [-0.07, 0.12] |
| MR-proANP | BMI | 0.01 | 2.24 | .025* | 0.02 | [0.00, 0.03] | 0.13 | [0.02, 0.25] |
| MR-proANP | SGLT2i | 0.19 | -0.63 | 0.532 | -0.12 | [-0.50, 0.26] | -0.03 | [-0.13, 0.07] |

**Supplementary Table 7k**

**Supplementary Table 7k.** SEM results for HOMA-IR (sensitivity analysis with adjustment for SGLT2i) – Combined model for NT-proBNP. Explained variance R^2^=0.13 for ∆NT-proBNP and R^2^=0.28 for NT-proBNP. *NT-proBNP* refers to the natural log-transformed fasting peptide concentration. *∆NT-proBNP* refers to the change in log-transformed concentrations (ln(postprandial) - ln(fasting)).

| **Outcome** | **Predictor** | **SE** | **Z** | **p** | **b** | **95% CI (b)** | **b*** | **95% CI (b*)** |
| --- | --- | --- | --- | --- | --- | --- | --- | --- |
| ∆NT-proBNP | NT-proBNP | 0.01 | -2.81 | .005** | -0.02 | [-0.03, -0.01] | -0.2 | [-0.33, -0.06] |
| ∆NT-proBNP | HOMA-IR | 0.01 | -1.48 | 0.139 | -0.02 | [-0.05, 0.01] | -0.11 | [-0.25, 0.03] |
| ∆NT-proBNP | age | 0.01 | 4.88 | < .001*** | 0.04 | [0.02, 0.05] | 0.34 | [0.21, 0.47] |
| ∆NT-proBNP | sex | 0.02 | -1.04 | 0.296 | -0.02 | [-0.05, 0.02] | -0.06 | [-0.18, 0.05] |
| ∆NT-proBNP | BMI | 0 | 0.76 | 0.446 | 0 | [-0.00, 0.01] | 0.06 | [-0.09, 0.20] |
| ∆NT-proBNP | SGLT2i | 0.05 | 2.8 | .005** | 0.14 | [0.04, 0.23] | 0.17 | [0.05, 0.28] |
| NT-proBNP | HOMA-IR | 0.12 | -2.31 | .021* | -0.29 | [-0.53, -0.04] | -0.15 | [-0.28, -0.02] |
| NT-proBNP | age | 0.06 | 9.35 | < .001*** | 0.54 | [0.43, 0.66] | 0.52 | [0.43, 0.60] |
| NT-proBNP | sex | 0.16 | 1.55 | 0.120 | 0.25 | [-0.06, 0.56] | 0.08 | [-0.02, 0.19] |
| NT-proBNP | BMI | 0.02 | 2.43 | .015* | 0.04 | [0.01, 0.08] | 0.16 | [0.03, 0.29] |
| NT-proBNP | SGLT2i | 0.43 | -0.35 | 0.727 | -0.15 | [-1.00, 0.70] | -0.02 | [-0.13, 0.09] |

**Supplementary Table 7l**

**Supplementary Table 7l.** SEM results for HOMA-IR (sensitivity analysis with adjustment for SGLT2i) – Combined model for Copeptin. Explained variance R^2^=0.13 for ∆Copeptin and R^2^=0.17 for Copeptin. *Copeptin* refers to the natural log-transformed fasting peptide concentration. *∆Copeptin* refers to the change in log-transformed concentrations (ln(postprandial) - ln(fasting)).

| **Outcome** | **Predictor** | **SE** | **Z** | **p** | **b** | **95% CI (b)** | **b*** | **95% CI (b*)** |
| --- | --- | --- | --- | --- | --- | --- | --- | --- |
| ∆Copeptin | Copeptin | 0.03 | -5.42 | < .001*** | -0.16 | [-0.22, -0.10] | -0.36 | [-0.48, -0.24] |
| ∆Copeptin | HOMA-IR | 0.03 | 0.15 | 0.885 | 0 | [-0.06, 0.07] | 0.01 | [-0.13, 0.15] |
| ∆Copeptin | age | 0.02 | 0.93 | 0.352 | 0.01 | [-0.02, 0.05] | 0.06 | [-0.06, 0.18] |
| ∆Copeptin | sex | 0.04 | 0.29 | 0.769 | 0.01 | [-0.07, 0.10] | 0.02 | [-0.11, 0.14] |
| ∆Copeptin | BMI | 0 | 2.14 | .032* | 0.01 | [0.00, 0.02] | 0.16 | [0.02, 0.30] |
| ∆Copeptin | SGLT2i | 0.11 | -0.05 | 0.960 | -0.01 | [-0.23, 0.21] | 0 | [-0.12, 0.12] |
| Copeptin | HOMA-IR | 0.07 | 1.45 | 0.148 | 0.11 | [-0.04, 0.25] | 0.1 | [-0.04, 0.24] |
| Copeptin | age | 0.03 | 2.73 | .006** | 0.09 | [0.03, 0.16] | 0.17 | [0.05, 0.28] |
| Copeptin | sex | 0.09 | -4.55 | < .001*** | -0.42 | [-0.60, -0.24] | -0.27 | [-0.38, -0.16] |
| Copeptin | BMI | 0.01 | 2.3 | .021* | 0.02 | [0.00, 0.04] | 0.16 | [0.03, 0.30] |
| Copeptin | SGLT2i | 0.25 | 0.44 | 0.658 | 0.11 | [-0.38, 0.60] | 0.03 | [-0.09, 0.14] |

**Supplementary Table 8a**

**Supplementary Table 8a.** SEM results for HOMA-IR – Stroke Subanalysis for MR-proANP. Explained variance R^2^=0.05 for ∆MR-proANP and R^2^=0.42 for MR-proANP. *MR-proANP* refers to the natural log-transformed fasting peptide concentration. *∆MR-proANP* refers to the change in log-transformed concentrations (ln(postprandial) - ln(fasting)).

| **Outcome** | **Predictor** | **SE** | **Z** | **p** | **b** | **95% CI (b)** | **b*** | **95% CI (b*)** |
| --- | --- | --- | --- | --- | --- | --- | --- | --- |
| ∆MR-proANP | MR-proANP | 0.03 | -2.66 | .008** | -0.07 | [-0.13, -0.02] | -0.26 | [-0.45, -0.07] |
| ∆MR-proANP | HOMA-IR | 0.03 | -1.58 | .113 | -0.04 | [-0.09, 0.01] | -0.15 | [-0.32, 0.03] |
| ∆MR-proANP | age | 0.01 | 1.29 | .196 | 0.02 | [-0.01, 0.05] | 0.13 | [-0.06, 0.32] |
| ∆MR-proANP | sex | 0.03 | 0.64 | .519 | 0.02 | [-0.04, 0.08] | 0.05 | [-0.10, 0.20] |
| ∆MR-proANP | BMI | 0.00 | 0.14 | .885 | 0.00 | [-0.01, 0.01] | 0.01 | [-0.16, 0.19] |
| MR-proANP | HOMA-IR | 0.07 | -3.25 | .001** | -0.22 | [-0.36, -0.09] | -0.23 | [-0.36, -0.09] |
| MR-proANP | age | 0.03 | 11.04 | < .001*** | 0.33 | [0.27, 0.39] | 0.65 | [0.57, 0.73] |
| MR-proANP | sex | 0.08 | 0.75 | .454 | 0.06 | [-0.10, 0.22] | 0.04 | [-0.07, 0.16] |
| MR-proANP | BMI | 0.01 | 1.80 | .072 | 0.02 | [-0.00, 0.04] | 0.12 | [-0.01, 0.26] |

**Supplementary Table 8b**

**Supplementary Table 8b.** SEM results for HOMA-IR – Stroke Subanalysis for NT-proBNP. Explained variance R^2^=0.08 for ∆NT-proBNP and R^2^=0.34 for NT-proBNP. *NT-proBNP* refers to the natural log-transformed fasting peptide concentration. *∆NT-proBNP* refers to the change in log-transformed concentrations (ln(postprandial) - ln(fasting)).

| **Outcome** | **Predictor** | **SE** | **Z** | **p** | **b** | **95% CI (b)** | **b*** | **95% CI (b*)** |
| --- | --- | --- | --- | --- | --- | --- | --- | --- |
| ∆NT-proBNP | NT-proBNP | 0.01 | -2.15 | .032* | -0.02 | [-0.04, -0.00] | -0.19 | [-0.37, -0.02] |
| ∆NT-proBNP | HOMA-IR | 0.02 | -0.73 | .468 | -0.01 | [-0.05, 0.02] | -0.06 | [-0.24, 0.11] |
| ∆NT-proBNP | age | 0.01 | 3.53 | < .001*** | 0.03 | [0.01, 0.05] | 0.32 | [0.15, 0.48] |
| ∆NT-proBNP | sex | 0.02 | -1.37 | .172 | -0.03 | [-0.07, 0.01] | -0.10 | [-0.25, 0.04] |
| ∆NT-proBNP | BMI | 0.00 | 1.27 | .205 | 0.00 | [-0.00, 0.01] | 0.11 | [-0.06, 0.28] |
| NT-proBNP | HOMA-IR | 0.15 | -2.31 | .021* | -0.35 | [-0.65, -0.05] | -0.17 | [-0.31, -0.03] |
| NT-proBNP | age | 0.07 | 8.88 | < .001*** | 0.59 | [0.46, 0.72] | 0.56 | [0.46, 0.66] |
| NT-proBNP | sex | 0.18 | 2.37 | .018* | 0.42 | [0.07, 0.78] | 0.15 | [0.03, 0.27] |
| NT-proBNP | BMI | 0.02 | 1.48 | .140 | 0.03 | [-0.01, 0.07] | 0.11 | [-0.03, 0.25] |

**Supplementary Table 8c**

**Supplementary Table 8c.** SEM results for HOMA-IR – Stroke Subanalysis for Copeptin. Explained variance R^2^=0.16 for ∆Copeptin and R^2^=0.20 for Copeptin. *Copeptin* refers to the natural log-transformed fasting peptide concentration. *∆Copeptin* refers to the change in log-transformed concentrations (ln(postprandial) - ln(fasting)).

| **Outcome** | **Predictor** | **SE** | **Z** | **p** | **b** | **95% CI (b)** | **b*** | **95% CI (b*)** |
| --- | --- | --- | --- | --- | --- | --- | --- | --- |
| ∆Copeptin | Copeptin | 0.04 | -5.30 | < .001*** | -0.19 | [-0.27, -0.12] | -0.42 | [-0.56, -0.28] |
| ∆Copeptin | HOMA-IR | 0.04 | -0.20 | .838 | -0.01 | [-0.09, 0.07] | -0.02 | [-0.18, 0.15] |
| ∆Copeptin | age | 0.02 | 1.20 | .232 | 0.02 | [-0.01, 0.06] | 0.09 | [-0.06, 0.23] |
| ∆Copeptin | sex | 0.05 | -0.37 | .710 | -0.02 | [-0.12, 0.08] | -0.03 | [-0.18, 0.12] |
| ∆Copeptin | BMI | 0.01 | 2.10 | .036* | 0.01 | [0.00, 0.02] | 0.18 | [0.01, 0.34] |
| Copeptin | HOMA-IR | 0.09 | 0.97 | .333 | 0.08 | [-0.08, 0.25] | 0.08 | [-0.08, 0.24] |
| Copeptin | age | 0.04 | 2.99 | .003** | 0.11 | [0.04, 0.19] | 0.21 | [0.08, 0.35] |
| Copeptin | sex | 0.10 | -4.63 | < .001*** | -0.47 | [-0.66, -0.27] | -0.32 | [-0.45, -0.20] |
| Copeptin | BMI | 0.01 | 2.04 | .041* | 0.02 | [0.00, 0.05] | 0.17 | [0.01, 0.32] |

**Supplementary Table 9a**

**Supplementary Table 9a.** SEM results for HOMA-IR – ACS Subanalysis for MR-proANP. Explained variance R^2^=0.13 for ∆MR-proANP and R^2^=0.37 for MR-proANP. *MR-proANP* refers to the natural log-transformed fasting peptide concentration. *∆MR-proANP* refers to the change in log-transformed concentrations (ln(postprandial) - ln(fasting)).

| **Outcome** | **Predictor** | **SE** | **Z** | **p** | **b** | **95% CI (b)** | **b*** | **95% CI (b*)** |
| --- | --- | --- | --- | --- | --- | --- | --- | --- |
| ∆MR-proANP | MR-proANP | 0.04 | -2.73 | .006** | -0.12 | [-0.20, -0.03] | -0.38 | [-0.63, -0.12] |
| ∆MR-proANP | HOMA-IR | 0.03 | -1.98 | .048* | -0.07 | [-0.14, -0.00] | -0.26 | [-0.52, -0.01] |
| ∆MR-proANP | age | 0.02 | 1.36 | .174 | 0.03 | [-0.01, 0.07] | 0.19 | [-0.08, 0.46] |
| ∆MR-proANP | sex | 0.05 | 1.00 | .319 | 0.05 | [-0.05, 0.15] | 0.11 | [-0.10, 0.32] |
| ∆MR-proANP | BMI | 0.00 | 0.67 | .506 | 0.00 | [-0.01, 0.01] | 0.09 | [-0.17, 0.35] |
| MR-proANP | HOMA-IR | 0.09 | -1.61 | .107 | -0.15 | [-0.33, 0.03] | -0.18 | [-0.39, 0.04] |
| MR-proANP | age | 0.05 | 6.11 | < .001*** | 0.30 | [0.20, 0.40] | 0.59 | [0.45, 0.74] |
| MR-proANP | sex | 0.14 | 0.29 | .772 | 0.04 | [-0.23, 0.31] | 0.03 | [-0.16, 0.21] |
| MR-proANP | BMI | 0.01 | 1.41 | .158 | 0.02 | [-0.01, 0.04] | 0.16 | [-0.06, 0.38] |

**Supplementary Table 9b**

**Supplementary Table 9b.** SEM results for HOMA-IR – ACS Subanalysis for NT-proBNP. Explained variance R^2^=0.17 for ∆NT-proBNP and R^2^=0.23 for NT-proBNP. *NT-proBNP* refers to the natural log-transformed fasting peptide concentration. *∆NT-proBNP* refers to the change in log-transformed concentrations (ln(postprandial) - ln(fasting)).

| **Outcome** | **Predictor** | **SE** | **Z** | **p** | **b** | **95% CI (b)** | **b*** | **95% CI (b*)** |
| --- | --- | --- | --- | --- | --- | --- | --- | --- |
| ∆NT-proBNP | NT-proBNP | 0.02 | -0.85 | .398 | -0.01 | [-0.04, 0.02] | -0.10 | [-0.34, 0.13] |
| ∆NT-proBNP | HOMA-IR | 0.03 | -0.38 | .708 | -0.01 | [-0.07, 0.04] | -0.05 | [-0.31, 0.21] |
| ∆NT-proBNP | age | 0.02 | 3.34 | .001*** | 0.05 | [0.02, 0.08] | 0.41 | [0.19, 0.62] |
| ∆NT-proBNP | sex | 0.04 | 0.22 | .824 | 0.01 | [-0.07, 0.09] | 0.02 | [-0.19, 0.23] |
| ∆NT-proBNP | BMI | 0.00 | -0.36 | .722 | -0.00 | [-0.01, 0.01] | -0.05 | [-0.31, 0.22] |
| NT-proBNP | HOMA-IR | 0.20 | -2.57 | .010* | -0.52 | [-0.91, -0.12] | -0.32 | [-0.55, -0.09] |
| NT-proBNP | age | 0.11 | 3.66 | < .001*** | 0.39 | [0.18, 0.60] | 0.39 | [0.20, 0.58] |
| NT-proBNP | sex | 0.30 | -0.51 | .607 | -0.15 | [-0.73, 0.43] | -0.05 | [-0.25, 0.15] |
| NT-proBNP | BMI | 0.03 | 2.53 | .012* | 0.07 | [0.02, 0.13] | 0.32 | [0.08, 0.55] |

**Supplementary Table 9c**

**Supplementary Table 9c.** SEM results for HOMA-IR – ACS Subanalysis for Copeptin. Explained variance R^2^=0.10 for ∆Copeptin and R^2^=0.08 for Copeptin. *Copeptin* refers to the natural log-transformed fasting peptide concentration. *∆Copeptin* refers to the change in log-transformed concentrations (ln(postprandial) - ln(fasting)).

| **Outcome** | **Predictor** | **SE** | **Z** | **p** | **b** | **95% CI (b)** | **b*** | **95% CI (b*)** |
| --- | --- | --- | --- | --- | --- | --- | --- | --- |
| ∆Copeptin | Copeptin | 0.05 | -2.12 | .034* | -0.10 | [-0.19, -0.01] | -0.26 | [-0.49, -0.03] |
| ∆Copeptin | HOMA-IR | 0.06 | 0.38 | .706 | 0.02 | [-0.09, 0.14] | 0.05 | [-0.22, 0.33] |
| ∆Copeptin | age | 0.03 | 0.26 | .796 | 0.01 | [-0.05, 0.07] | 0.03 | [-0.21, 0.27] |
| ∆Copeptin | sex | 0.09 | 1.12 | .263 | 0.10 | [-0.07, 0.27] | 0.13 | [-0.10, 0.36] |
| ∆Copeptin | BMI | 0.01 | 0.72 | .470 | 0.01 | [-0.01, 0.02] | 0.10 | [-0.18, 0.38] |
| Copeptin | HOMA-IR | 0.15 | 0.86 | .392 | 0.13 | [-0.17, 0.43] | 0.12 | [-0.15, 0.40] |
| Copeptin | age | 0.08 | 0.67 | .504 | 0.05 | [-0.11, 0.22] | 0.08 | [-0.16, 0.32] |
| Copeptin | sex | 0.22 | -1.23 | .219 | -0.27 | [-0.71, 0.16] | -0.15 | [-0.38, 0.08] |
| Copeptin | BMI | 0.02 | 1.07 | .285 | 0.02 | [-0.02, 0.06] | 0.15 | [-0.12, 0.43] |

**Supplementary Table 10**

**Supplementary Table 10.** Index event-visit interval and MR-proANP, NT-proBNP and copeptin. Association between the interval from the index event to the deep phenotyping visit in days and fasting concentrations and postprandial responses of natriuretic peptides and copeptin. Analyses were restricted to the pooled acute event groups (ACS and stroke). Values are standardized coefficients with 95% CI for the interval. Estimates were obtained by entering the interval as an additional covariate into the same structural equation models as the corresponding main analyses, adjusted for age, sex and BMI. *Peptide* refers to the natural log-transformed fasting peptide concentration. *∆Peptide* refers to the change in log-transformed concentrations (ln(postprandial) - ln(fasting)).

| **Outcome** | **Model for T2D** | **Model for HOMA-IR** |
| --- | --- | --- |
| Fasting MR-proANP | 0.01 [-0.09, 0.11] | 0.01 [-0.09, 0.10] |
| ΔMR-proANP | -0.06 [-0.18, 0.06] | −0.06 [-0.18, 0.06] |
| Fasting NT-proBNP | -0.06 [-0.16, 0.05] | -0.07 [-0.17, 0.04] |
| ΔNT-proBNP | -0.01 [-0.13, 0.11] | -0.01 [-0.13, 0.11] |
| Fasting copeptin | -0.02 [-0.14, 0.10] | -0.02 [-0.14, 0.10] |
| ΔCopeptin | 0.13 [0.01, 0.25] | 0.12 [0.01, 0.24] |

**Supplementary Table 11a**

**Supplementary Table 11a.** SEM results for antidiabetic medication use within the HR group for MR-proANP. Explained variance R²=0.36 for MR-proANP and R²=0.22 for ΔMR-proANP. MR-proANP refers to the natural log-transformed fasting peptide concentration. ∆MR-proANP refers to the change in log-transformed concentrations (ln(postprandial) - ln(fasting)).

| **Outcome** | **Predictor** | | **SE** | **Z** | **p** | **b** | **95% CI (b)** | **b*** | **95% CI (b*)** |
| --- | --- | --- | --- | --- | --- | --- | --- | --- | --- |
| ΔMR-proANP | | MR-proANP | 0.02 | -3.04 | .002** | -0.07 | [-0.12, -0.03] | -0.34 | [-0.55, -0.13] |
| ΔMR-proANP | | age | 0.02 | -0.06 | 0.95 | 0 | [-0.03, 0.03] | -0.01 | [-0.23, 0.21] |
| ΔMR-proANP | | sex | 0.03 | 0.82 | 0.41 | 0.02 | [-0.03, 0.08] | 0.08 | [-0.11, 0.26] |
| ΔMR-proANP | | BMI | 0 | -2.33 | .020* | 0 | [-0.01, -0.00] | -0.22 | [-0.41, -0.04] |
| ΔMR-proANP | | GLP-1 RA | 0.03 | -2.08 | .037* | -0.07 | [-0.13, -0.00] | -0.19 | [-0.37, -0.02] |
| ΔMR-proANP | | SGLT2i | 0.03 | 1.25 | 0.212 | 0.04 | [-0.02, 0.10] | 0.12 | [-0.06, 0.30] |
| MR-proANP | | age | 0.05 | 6.74 | < .001*** | 0.36 | [0.25, 0.46] | 0.57 | [0.44, 0.70] |
| MR-proANP | | sex | 0.12 | -0.98 | 0.326 | -0.12 | [-0.35, 0.12] | -0.08 | [-0.25, 0.08] |
| MR-proANP | | BMI | 0.01 | -0.21 | 0.836 | 0 | [-0.02, 0.02] | -0.02 | [-0.19, 0.15] |
| MR-proANP | | GLP-1 RA | 0.14 | 0.38 | 0.701 | 0.05 | [-0.21, 0.32] | 0.03 | [-0.13, 0.19] |
| MR-proANP | | SGLT2i | 0.12 | -1.78 | 0.075 | -0.22 | [-0.47, 0.02] | -0.15 | [-0.31, 0.01] |

**Supplementary Table 11b**

**Supplementary Table 11b.** SEM results for antidiabetic medication use within the HR group for NT-proBNP. Explained variance R²=0.27 for NT-proBNP and R²=0.11 for ΔNT-proBNP. NT-proBNP refers to the natural log-transformed fasting peptide concentration. ∆NT-proBNP refers to the change in log-transformed concentrations (ln(postprandial) - ln(fasting)).

| **Outcome** | **Predictor** | **SE** | **Z** | **p** | **b** | **95% CI (b)** | **b*** | **95% CI (b*)** |
| --- | --- | --- | --- | --- | --- | --- | --- | --- |
| ΔNT-proBNP | NT-proBNP | 0.01 | -0.78 | 0.433 | -0.01 | [-0.03, 0.01] | -0.09 | [-0.30, 0.13] |
| ΔNT-proBNP | age | 0.01 | 1.63 | 0.104 | 0.02 | [-0.00, 0.05] | 0.18 | [-0.03, 0.40] |
| ΔNT-proBNP | sex | 0.03 | 0.36 | 0.715 | 0.01 | [-0.05, 0.07] | 0.04 | [-0.16, 0.23] |
| ΔNT-proBNP | BMI | 0 | 0.26 | 0.791 | 0 | [-0.00, 0.00] | 0.03 | [-0.17, 0.23] |
| ΔNT-proBNP | GLP-1 RA | 0.03 | 2.33 | .020* | 0.08 | [0.01, 0.14] | 0.23 | [0.04, 0.41] |
| ΔNT-proBNP | SGLT2i | 0.03 | 1.83 | 0.067 | 0.06 | [-0.00, 0.11] | 0.18 | [-0.01, 0.37] |
| NT-proBNP | age | 0.11 | 5.33 | < .001*** | 0.56 | [0.35, 0.77] | 0.48 | [0.33, 0.63] |
| NT-proBNP | sex | 0.24 | 0.05 | 0.959 | 0.01 | [-0.45, 0.48] | 0 | [-0.17, 0.18] |
| NT-proBNP | BMI | 0.02 | -0.39 | 0.698 | -0.01 | [-0.04, 0.03] | -0.04 | [-0.22, 0.15] |
| NT-proBNP | GLP-1 RA | 0.27 | -0.2 | 0.839 | -0.05 | [-0.58, 0.47] | -0.02 | [-0.19, 0.16] |
| NT-proBNP | SGLT2i | 0.25 | -1.27 | 0.203 | -0.31 | [-0.80, 0.17] | -0.11 | [-0.29, 0.06] |

**Supplementary Table 11c**

**Supplementary Table 11c.** SEM results for antidiabetic medication use within the HR group for Copeptin. Explained variance R²=0.18 for Copeptin and R²=0.15 for ΔCopeptin. Copeptin refers to the natural log-transformed fasting peptide concentration. ∆Copeptin refers to the change in log-transformed concentrations (ln(postprandial) - ln(fasting)).

| **Outcome** | **Predictor** | **SE** | **Z** | **p** | **b** | **95% CI (b)** | **b*** | **95% CI (b*)** |
| --- | --- | --- | --- | --- | --- | --- | --- | --- |
| ΔCopeptin | Copeptin | 0.06 | -2.37 | .018* | -0.14 | [-0.25, -0.02] | -0.25 | [-0.45, -0.05] |
| ΔCopeptin | age | 0.03 | 2.22 | .026* | 0.08 | [0.01, 0.14] | 0.22 | [0.03, 0.41] |
| ΔCopeptin | sex | 0.08 | 1.21 | 0.225 | 0.1 | [-0.06, 0.26] | 0.13 | [-0.08, 0.34] |
| ΔCopeptin | BMI | 0.01 | 1.09 | 0.276 | 0.01 | [-0.00, 0.02] | 0.11 | [-0.09, 0.32] |
| ΔCopeptin | GLP-1 RA | 0.09 | -0.77 | 0.443 | -0.07 | [-0.23, 0.10] | -0.07 | [-0.26, 0.12] |
| ΔCopeptin | SGLT2i | 0.08 | 0.82 | 0.415 | 0.07 | [-0.09, 0.23] | 0.08 | [-0.11, 0.28] |
| Copeptin | age | 0.06 | 1.71 | 0.088 | 0.1 | [-0.02, 0.22] | 0.16 | [-0.02, 0.35] |
| Copeptin | sex | 0.14 | -3.58 | < .001*** | -0.49 | [-0.76, -0.22] | -0.35 | [-0.53, -0.18] |
| Copeptin | BMI | 0.01 | 1.41 | 0.159 | 0.01 | [-0.01, 0.03] | 0.14 | [-0.05, 0.34] |
| Copeptin | GLP-1 RA | 0.15 | 0.16 | 0.871 | 0.02 | [-0.27, 0.32] | 0.02 | [-0.17, 0.20] |
| Copeptin | SGLT2i | 0.14 | 1.99 | .047* | 0.28 | [0.00, 0.56] | 0.19 | [0.01, 0.37] |

**Supplementary Table 12**

**Supplementary Table 12.** Clinical characteristics of the stroke cohort. Data are presented as n (%) or median and interquartile range. Percentages for the total stroke cohort refer to n = 186, percentages within the ischemic stroke subgroup refer to n = 145. Percentages for categorical severity scores are calculated among participants with available data. The NIHSS and the TOAST aetiological classification are recorded only in participants with ischemic cerebral infarction according to the BeLOVE study protocol. The modified Rankin Scale was assessed at hospital admission for the index event.

| **Characteristic** | **n (%) or median [IQR]** |
| --- | --- |
| **Total stroke cohort (n = 186)** |  |
| **Type of cerebrovascular event** |  |
| Ischemic stroke | 145 (78.0) |
| Transient ischemic attack | 26 (14.0) |
| Central retinal artery occlusion | 8 (4.3) |
| Amaurosis fugax | 1 (0.5) |
| Intracerebral haemorrhage | 6 (3.2) |
| **Modified Rankin Scale at admission** |  |
| Median [IQR] | 1 [0; 2] |
| mRS 0-2 | 138 (77.5) |
| mRS > 2 | 40 (22.5) |
| Missing | 8 |
| **Ischemic stroke subgroup (n = 145)** |  |
| **Aetiology (TOAST classification)** |  |
| Cardioembolic | 40 (27.6) |
| Macroangiopathic (large-artery atherosclerosis) | 16 (11.0) |
| Microangiopathic (small-vessel occlusion) | 13 (9.0) |
| Other determined aetiology | 3 (2.1) |
| Undetermined aetiology | 73 (50.3) |
| **NIHSS at admission** |  |
| Median [IQR] | 2 [0; 3] |
| NIHSS 0-5 | 126 (88.7) |
| NIHSS > 5-10 | 11 (7.7) |
| NIHSS > 10 | 5 (3.5) |
| Missing | 3 |
| **Modified Rankin Scale at admission** |  |
| Median [IQR] | 2 [1; 3] |
| mRS 0-2 | 98 (71.0) |
| mRS > 2 | 40 (29.0) |
| Missing | 7 |

IQR, interquartile range; mRS, modified Rankin Scale; NIHSS, National Institutes of Health Stroke Scale; TOAST, Trial of Org 10172 in Acute Stroke Treatment

**Supplementary Table 13**

**Supplementary Table 13.** Contrast estimates comparing ACS and stroke. All contrasts are defined as ACS minus stroke. For the acute CV event models, values are the contrast between the ACS and stroke coefficients (each relative to the HR group); for the T2D and HOMA-IR models, values are the exposure × event type interaction term (T2D coded as yes vs. no [reference]; HOMA-IR modeled continuously). Unstandardized coefficients with 95% CI.

| **Exposure** | **Peptide** | **Fasting** | **Δ** |
| --- | --- | --- | --- |
| Acute CV event (ACS vs. stroke) | MR-proANP | -0.002 [-0.276; 0.272] | -0.016 [-0.083; 0.052] |
|  | NT-proBNP | 0.050 [-0.526; 0.625] | -0.051 [-0.121; 0.018] |
|  | Copeptin | -0.290 [-0.622; 0.042] | 0.012 [-0.151; 0.176] |
| T2D × event type | MR-proANP | -0.156 [-0.458; 0.145] | 0.004 [-0.106; 0.114] |
|  | NT-proBNP | -0.488 [-1.160; 0.185] | -0.053 [-0.135; 0.029] |
|  | Copeptin | -0.480 [-0.909; -0.050] | -0.062 [-0.243; 0.120] |
| HOMA-IR × event type | MR-proANP | 0.085 [-0.106; 0.277] | -0.014 [-0.084; 0.056] |
|  | NT-proBNP | 0.067 [-0.356; 0.490] | -0.024 [-0.077; 0.029] |
|  | Copeptin | 0.046 [-0.242; 0.334] | 0.024 [-0.094; 0.143] |

**Supplementary Table 14**

**Supplementary Table 14.** Missing data per SEM. Values are participants excluded due to missing data / eligible participants (%). In the acute event models, analyses were restricted to participants with T2D in all groups.

| **Exposure** | **Peptide** | **Group** | **Eligible, n** | **Missing, n (%)** |
| --- | --- | --- | --- | --- |
| Acute CV event (ACS, stroke) | MR-proANP | HR | 106 | 8 (7.5) |
|  | MR-proANP | ACS | 26 | 1 (3.8) |
|  | MR-proANP | Stroke | 32 | 0 (0.0) |
|  | MR-proANP | Total | 164 | 9 (5.5) |
|  |  |  |  |  |
|  | NT-proBNP | HR | 106 | 8 (7.5) |
|  | NT-proBNP | ACS | 26 | 1 (3.8) |
|  | NT-proBNP | Stroke | 32 | 0 (0.0) |
|  | NT-proBNP | Total | 164 | 9 (5.5) |
|  |  |  |  |  |
|  | Copeptin | HR | 106 | 12 (11.3) |
|  | Copeptin | ACS | 26 | 2 (7.7) |
|  | Copeptin | Stroke | 32 | 0 (0.0) |
|  | Copeptin | Total | 164 | 14 (8.5) |
|  |  |  |  |  |
| T2D in participants with acute CV event | MR-proANP | ACS | 81 | 8 (9.9) |
|  | MR-proANP | Stroke | 186 | 13 (7.0) |
|  | MR-proANP | Total | 267 | 21 (7.9) |
|  |  |  |  |  |
|  | NT-proBNP | ACS | 81 | 7 (8.6) |
|  | NT-proBNP | Stroke | 186 | 11 (5.9) |
|  | NT-proBNP | Total | 267 | 18 (6.7) |
|  |  |  |  |  |
|  | Copeptin | ACS | 81 | 14 (17.3) |
|  | Copeptin | Stroke | 186 | 17 (9.1) |
|  | Copeptin | Total | 267 | 31 (11.6) |
|  |  |  |  |  |
| Insulin resistance in participants with acute CV event | MR-proANP | ACS | 81 | 8 (9.9) |
|  | MR-proANP | Stroke | 186 | 14 (7.5) |
|  | MR-proANP | Total | 267 | 22 (8.2) |
|  |  |  |  |  |
|  | NT-proBNP | ACS | 81 | 7 (8.6) |
|  | NT-proBNP | Stroke | 186 | 12 (6.5) |
|  | NT-proBNP | Total | 267 | 19 (7.1) |
|  |  |  |  |  |
|  | Copeptin | ACS | 81 | 14 (17.3) |
|  | Copeptin | Stroke | 186 | 18 (9.7) |
|  | Copeptin | Total | 267 | 32 (12.0) |

**Supplementary Table 15**

**Supplementary Table 15.** Comparison of complete-case (CC) and full information maximum likelihood (FIML) estimates. Values are unstandardized regression coefficients (b). All models were adjusted for age, sex and BMI.

|  |  | **Exposure → Δ** | | | **Exposure → fasting** | | | **Fasting → Δ** | | |
| --- | --- | --- | --- | --- | --- | --- | --- | --- | --- | --- |
| **Exposure** | **Peptide** | CC | FIML | Diff. | CC | FIML | Diff. | CC | FIML | Diff. |
| Acute event  vs. HR | MR-proANP | 0.02 | 0.02 | 0.00 | 0.31 | 0.31 | 0.00 | -0.06 | -0.06 | 0.00 |
|  | NT-proBNP | 0.03 | 0.03 | 0.00 | 0.80 | 0.80 | 0.00 | -0.02 | -0.02 | 0.00 |
|  | Copeptin | -0.09 | -0.09 | 0.00 | 0.06 | 0.07 | 0.01 | -0.13 | -0.13 | 0.00 |
| T2D | MR-proANP | -0.00 | -0.00 | 0.00 | -0.03 | -0.03 | 0.00 | -0.07 | -0.07 | 0.00 |
|  | NT-proBNP | -0.02 | -0.02 | 0.00 | 0.25 | 0.25 | 0.00 | -0.02 | -0.02 | 0.00 |
|  | Copeptin | 0.04 | 0.04 | 0.00 | 0.12 | 0.11 | -0.01 | -0.16 | -0.16 | 0.00 |
| HOMA-IR | MR-proANP | -0.05 | -0.05 | 0.00 | -0.17 | -0.19 | -0.02 | -0.09 | -0.08 | 0.01 |
|  | NT-proBNP | -0.02 | -0.02 | 0.00 | -0.29 | -0.30 | -0.01 | -0.02 | -0.02 | 0.00 |
|  | Copeptin | 0.00 | 0.00 | 0.00 | 0.11 | 0.12 | 0.01 | -0.16 | -0.16 | 0.00 |
